# Selective interaction of SMCHD1 with chromatin is governed by LRIF1 and SMCHD1 ATPase activity

**DOI:** 10.1038/s41467-026-74427-9

**Published:** 2026-06-16

**Authors:** Flavia Constantinescu, Aleksander T. Szczurek, Tatyana B. Nesterova, Guifeng Wei, Mafalda Almeida, Jessica R. Kelley, Victoria Pustygina, Stephan Uphoff, Adam D. Cawte, Neil Brockdorff

**Affiliations:** 1 Developmental Epigenetics, Dept of Biochemistry University of Oxford, South Parks Road, Oxford, UK; 2 Epigenetic regulation of chromatin function, Dept of Biochemistry University of Oxford, South Parks Road, Oxford, UK; 3 Transposon lab, Dept of Biochemistry University of Oxford, South, Parks Road, Oxford, UK; 4 Chromatin Dynamics, Dept of Physics, University of Oxford, Parks Road, Oxford, UK; 5 Bacterial DNA repair and mutagenesis, Dept of Biochemistry University of Oxford, South Parks Road, Oxford, UK

**Keywords:** Dosage compensation, Gene silencing

## Abstract

The chromosomal protein SMCHD1 is a GHKL ATPase with important roles in epigenetic silencing, including on the inactive X chromosome (Xi). Mutations of SMCHD1 have been linked to facioscapulohumeral muscular dystrophy (FSHD) and Bosma arrhinia microphthalmia syndrome (BAMS). Here, we use live-cell and single-molecule imaging to investigate SMCHD1 interactions with chromatin and its function in epigenetic silencing. We show that chromatin binding of SMCHD1 genome-wide, including on the Xi, is critically dependent on the protein LRIF1 that mediates interaction with H3K9me2/3-modified nucleosomes. Engineered mutations in the GHKL ATPase domain demonstrate that ATP hydrolysis is required for selective enrichment of SMCHD1 at specific chromatin regions, which is critical for X-linked gene silencing. Conversely, gain-of-function BAMS-associated mutations are linked to accelerated Xi recruitment and gene silencing, or increased Xi compaction. Together, our findings advance understanding of SMCHD1 recruitment and function on the Xi and at other target sites in the genome.

## Abstract (web summary)

SMCHD1 is a key player in X chromosome inactivation in mammals and has been linked to two human disorders. Here, the authors present insight into SMCHD1’s mode of interaction and functionality at its target sites.

## Introduction

X-chromosome inactivation (XCI) evolved in mammals to ensure dosage compensation of X-linked genes in XX females relative to XY males^1^. The master regulator of this process is the X-inactive specific transcript (Xist), a 15–18 kb long noncoding RNA (lncRNA) that accumulates *in cis* over the future inactive X (Xi) chromosome^2–5^. Xist mediates stepwise recruitment of factors which modify the underlying chromatin and ultimately lead to chromosome-wide silencing that is then maintained through all subsequent cell divisions. One of these factors is the epigenetic modifier structural maintenance of chromosome hinge domain containing 1 (SMCHD1), whose absence causes female-specific mid-gestation embryonic lethality^6,7^. SMCHD1 has two characteristic conserved domains, a C-terminal SMC hinge domain, found also in condensin, cohesin, and other proteins belonging to the SMC family of chromosomal proteins^8^, and an N-terminal GHKL ATPase domain, also found in a range of ATPases including DNA gyrase and the MORC family of epigenetic repressors^9^.

SMCHD1 is recruited to Xi at a relatively late stage following initiation of XCI^10,11^, leading to suggestions that it is principally involved in stable maintenance of the inactive state. However, more recent work has determined that SMCHD1 contributes to the silencing of a significant subset of X-linked genes, referred to as SMCHD1-dependent and SMCHD1-partially dependent genes respectively^11–14^. Additionally, SMCHD1 function is required for DNA methylation of CpG-islands of most X-linked genes, a hallmark of stable long-term silencing, and for establishing the unique long-range 3D architecture of the Xi^10,13–17^. The mechanism of SMCHD1 function in X-linked gene silencing is not fully understood, although protein architecture similarities

with the MORC ATPase chromatin proteins suggest a role in chromatin compaction^18–20^. Consistent with this idea, there is evidence suggesting that SMCHD1 contributes to the compacted structure of the Xi chromosome^21^. Similarly, the mechanism for SMCHD1 recruitment to Xi is not well understood. Direct interaction with Xist RNA has been invoked^22^, but a recent study using stringent RNA-protein crosslinking did not support this conclusion^23^. Instead, evidence was obtained implying interaction of SMCHD1 with the Polycomb repressive complex 1 (PRC1)-mediated histone modification H2AK119ub1, either directly or via an intermediate factor^23^.

In addition to its role in XCI, SMCHD1 functions in the epigenetic repression of certain autosomal loci^15,16,24,25^. A notable case is the gene encoding DUX4 in humans, abnormal expression of which results in FSHD. Here SMCHD1 plays a role in DUX4 repression via the D4Z4 macrosatellite array, and loss-of-function mutations in SMCHD1 are causative in a proportion of FSHD2 patients^26–28^. An FSHD2 mutation has also been found in a second factor, LRIF1, which, together with proteins of the HP1 family, directs SMCHD1 to target sites marked by the histone modification H3K9me3^21,29,30^. Furthermore, SMCHD1 gain-of-function mutations in the GHKL ATPase domain have been linked to the rare disorder BAMS^27,28,31,32^ although one such mutation was also found in an FSHD2 patient^33^.

Structural analysis of SMCHD1 has shown that it forms a stable homodimer with an extended rod-like structure^30^. Dimerisation is mediated by the SMC hinge and the GHKL ATPase domains^30,34–36^. The functional importance of the GHKL ATPase is underscored by its requirement for SMCHD1 association with the Xi^30,37^, and the occurrence of GHKL ATPase mutations in FSHD2 and BAMS patients. However, the role of the GHKL ATPase in chromosomal targeting and/or the function of SMCHD1 in epigenetic repression is not well understood.

Here we employ live-cell imaging and single-particle tracking (SPT) to study the dynamic behaviour of SMCHD1 and, thus, shed light on the molecular mechanisms underlying its interaction with chromatin and functions in XCI. We show that LRIF1 plays an important role in loading SMCHD1 at target sites, including pericentric heterochromatin (PCH) and Xi. Furthermore, we demonstrate that SMCHD1 exhibits Xi-specific dynamic behaviour which is altered by mutations affecting the GHKL ATPase activity of the protein, including loss- and gain-of-function mutations associated with BAMS and FSHD2. Importantly, these mutations also affect SMCHD1 function in X-linked gene silencing and chromosome compaction.

## Results

### An mESC model for analysing SMCHD1 dynamic behaviour

**Fig. 1:**
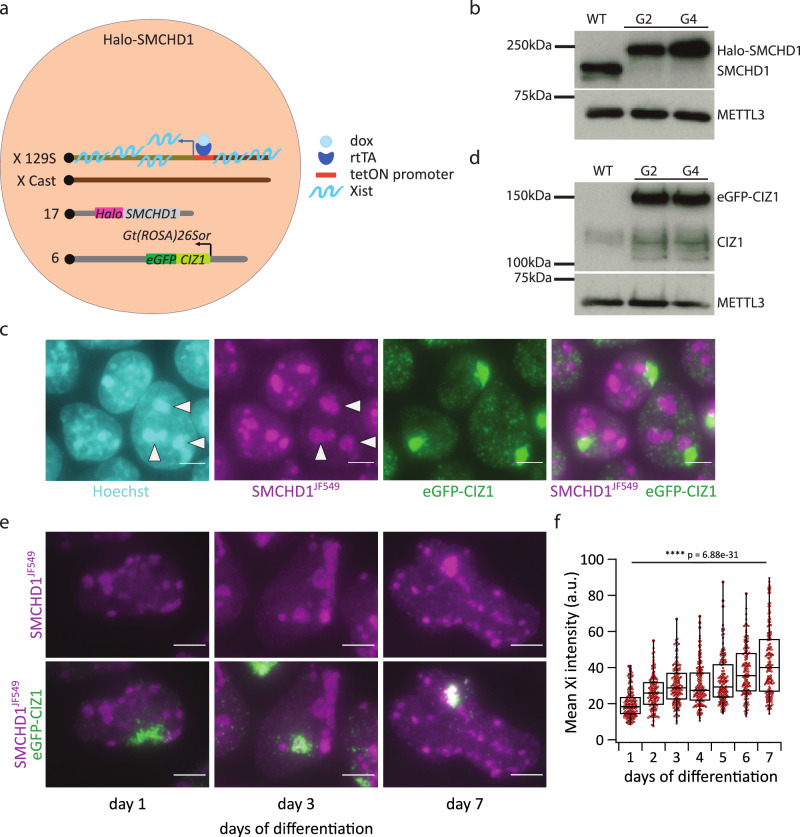
A model system to analyse dynamic interactions of SMCHD1 with chromatin. **a** Schematic of the Halo-SMCHD1 XX mESC line generated in the iXist-ChrX mESC line. **b** Western Blot showing the level of expression of Halo-SMCHD1 in two independent clones. Clone G2 was used for further experiments. METTL3 loading control **c** Representative live-cell images of Halo-SMCHD1 mESC line, taken after 24 h doxycycline addition. Arrowheads indicate selected PCH domains. Images are maximum-intensity projections, scale bar - 5 µm. (three independent replicates) **d** Western blot showing the expression of eGFP-tagged CIZ1 in two independent clones. METTL3 loading control **e** Representative live-cell images showing gradual accumulation of SMCHD1 over the Xi domain marked by eGFP-CIZ1 during mESC differentiation. Images are maximum-intensity projections, scale bar - 5 µm. **f** Quantification of SMCHD1 signal underlying the CIZ1 domain at each day during a 7-day live-cell imaging differentiation timecourse. Each dot represents a single cell, *n* = 136-159. Boxplots: centre lines indicate the median, box limits indicate the first and third quartiles, and whiskers extend to min/max data points. Statistical analysis was performed by a two-sided unpaired t-test. Source data are provided as a Source Data file.

To investigate SMCHD1 association with Xi we adapted iXist-ChrX, a previously established interspecific XX mESC line with the TetOn promoter driving doxycyline inducible Xist RNA expression on the 129S strain-derived X chromosome^38^. We first engineered a Halo-tag^39^ on the native SMCHD1 protein (Fig. 1a, b). Prior experiments (using a SNAP-tag^40^), indicated that C-terminal tagging affects SMCHD1 stability (Supplementary Fig. 1a), but an N-terminal Halo-tag had no detectable effect on the protein level (Fig. 1b). Furthermore, allelic transcription analysis using ChrRNA-seq^38^ affirmed that addition of the N-terminal Halo-tag had no effect on SMCHD1 function in XCI (Supplementary Fig. 1b).

Labelling of endogenously tagged SMCHD1 with the fluorescent Halo ligand JF549 revealed nuclear localised protein as expected. Interestingly, the majority of cells exhibited enrichment of SMCHD1 at pericentric heterochromatin (PCH) regions as determined by staining with Hoechst 33342 (Fig. 1c), contrasting with our previous analysis of SMCHD1 localisation by immunofluorescence (IF) in fixed mESCs^30^. Further investigation of this finding is described below.

To identify the Xi domain in live-cell imaging we introduced a transgene encoding eGFP-CIZ1 in the *Gt(ROSA)26Sor* locus (Fig. 1a). Expression of eGFP-CIZ1 was confirmed by western blot (Fig. 1d). CIZ1 binds to the E-repeat element of Xist RNA from the onset of XCI and continuously thereafter^41,42^, and consistent with this, eGFP-CIZ1 Xi domains were detected in the majority of cells following Xist RNA induction in undifferentiated mESCs (Fig. 1c, and Supplementary Fig. 1c). RNA-FISH analysis in mESCs confirmed the colocalization of eGFP-CIZ1 and Xist RNA (Supplementary Fig. 1d).

In previous work using IF in fixed cells we observed enrichment of SMCHD1 over the Xi only after several days of Xist RNA induction coupled to cellular differentiation^11^. To investigate Xi localisation of SMCHD1 by live-cell imaging we used a monolayer differentiation protocol^43^. We then quantified Halo-SMCHD1 enrichment over the Xi (eGFP-CIZ1 domains) in a live-cell imaging timecourse over 7 days of differentiation (Fig. 1e, f). Strong accumulation of SMCHD1 over the Xi was most clearly evident at late differentiation stages (Fig. 1e), consistent with prior studies^10,11^. However, quantitative analysis demonstrated a more graded accumulation of SMCHD1, with significant enrichment underlying CIZ1 domains detectable as early as 48 h after Xist RNA induction (Fig. 1f).

### SMCHD1 localisation to PCH is LRIF1 dependent

Prior analysis of SMCHD1 in mESCs using IF revealed enrichment at PCH regions in only a small proportion of cells^30^. Similarly, IF in the mESC line analysed herein does not show PCH accumulation of SMCHD1 in most cells (Supplementary Fig. 2a). However, Halo-tag labelling followed by formaldehyde fixation displays the same population-wide pattern observed in live-cell imaging (Supplementary Fig. 2b), suggesting that absence of this localisation pattern using IF is not attributable to fixation but likely due to epitope masking when the protein is bound to PCH regions. We note that these observations support findings from a proteomic analysis that defined SMCHD1 amongst proteins enriched at PCH in mESCs^44^.

**Fig. 2:**
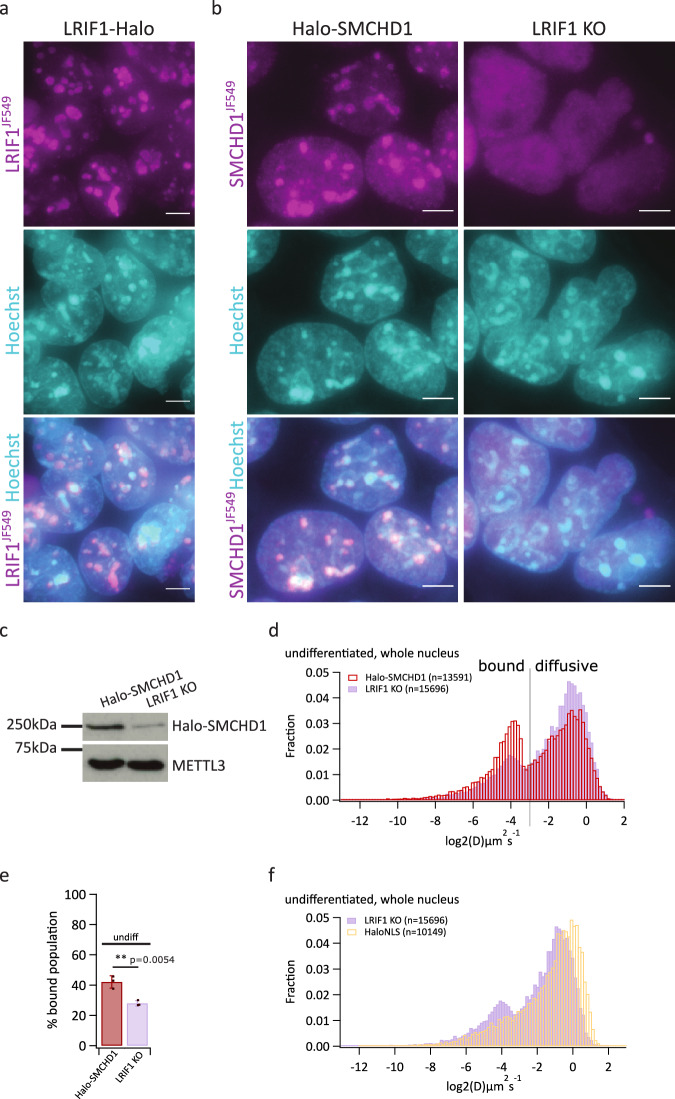
LRIF1 is required for SMCHD1 binding to chromatin in mESCs. **a** Representative live-cell images of LRIF1-Halo mESC line, taken 24 h after doxycycline addition. (two independent replicates) **b** Representative live images of Halo-SMCHD1 and Lrif1^-/-^ (LRIF KO) mESC lines. (three independent replicates) **c** Western blot showing SMCHD1 protein levels in the presence and absence of LRIF1 (three independent replicates). **d** Normalised histogram of diffusion coefficients of tracks from across the whole nucleus. Cells were undifferentiated, kept for 24 h with doxycycline. **e** Bar plots showing the average percentage of bound molecules of SMCHD1 (3 independent replicates each). Error bars represent the STDEV. Statistical analysis was by two-sided unpaired t-test. **f** In purple, same as in 2 d. In yellow, normalised histogram of diffusion coefficients of tracks from across the whole nucleus; cells are undifferentiated Halo-3xNLS mESCs, kept for 24 h with doxycycline. All images are maximum-intensity projections. Scale bars correspond to 5 µm. undiff = undifferentiated. Source data are provided as a Source Data file.

PCH accumulation of SMCHD1 could be explained by its reported interaction with LRIF1 and HP1 family proteins to mediate association with the chromatin mark H3K9me3^21,30^. To explore this hypothesis, we used CRISPR/Cas9 genome engineering to generate iXist-ChrX mESCs with a Halo-tag on the C-terminus of LRIF1 (tagging all three known isoforms) (Supplementary Fig. 2c), and then performed live-cell imaging after labelling cells with fluorescent JF549 Halo ligand. As shown in Fig. 2a, LRIF1-Halo is strongly enriched over PCH domains in a pattern that is indistinguishable from that seen for Halo-SMCHD1. Localisation of LRIF1 to PCH domains has been reported previously^45^, but was seen in only a small proportion of cells in a population, probably due to the use of antibody-based staining rather than live-cell imaging.

To test if LRIF1 is required for SMCHD1 localisation at PCH domains we generated CRISPR/Cas9-mediated knock-out of the gene encoding LRIF1 in the Halo-SMCHD1 mESC line described above (Supplementary Fig. 2c, d). The engineered gene knock-out was designed to ensure loss of all three LRIF1 isoforms. The *Lrif1*^*-/-*^ mESCs exhibit a marked loss of SMCHD1 enrichment at PCH regions in most cells, even though the regions are still clearly present as highlighted by staining with Hoechst 33342 dye (Fig. 2b). This observation confirms that LRIF1 plays a central role in the observed recruitment of SMCHD1 to PCH. SMCHD1 localisation to PCH regions remains detectable in a small proportion of cells (Supplementary Fig. 2e). Of note, whilst absence of LRIF1 also leads to a marked decrease in the protein level of SMCHD1 (Fig. 2c), an analysis of SMCHD1 depletion using Halo-PROTAC-E demonstrates that this effect does not underlie observed changes in SMCHD1 localisation (Supplementary Fig. 3).

To assess changes in the dynamic behaviour of SMCHD1, we made use of a photoactivable Halo-tag ligand (PA-JF549), and highly inclined and laminated optical sheet (HILO) microscopy with high temporal resolution (15 ms frames)^46^. This allowed us to perform Single Particle Tracking (SPT) analysis to record the localisations, movement, and diffusion coefficients of individual Halo-SMCHD1 molecules in live cells. In wild-type cells, a histogram plot of the diffusion coefficients shows two populations, an immobile, chromatin bound population with a Log_2_ diffusion coefficient peak around −4 (0.0625 µm^2^/s) and a fast, freely diffusing, population with a peak at around −1 (0.5 µm^2^/s) (Fig. 2d). The bound population is defined herein as the percentage of tracks whose Log2 diffusion coefficient is smaller than −3 (Fig. 2e). The threshold value −3 was chosen as it sits, across the various conditions analysed in this study, close to the minimum between the peaks of bound and unbound populations. Importantly, this analysis method captures differences between biological contexts with a similar efficiency as the methods relying on Gaussian fitting or kinetic modelling in SPOT-ON, but does not require large datasets^47,48^ (Supplementary Fig. 2f).

In *Lrif1*^*-/-*^ mESCs, the bound population is significantly reduced (Fig. 2d-e), exhibiting similar dynamic behaviour to Halo-tag protein with a nuclear localisation signal (Halo-3xNLS) which is not expected to interact with chromatin (Fig. 2f). These observations are consistent with subcellular fractionation experiments which reported loss of SMCHD1 from the chromatin bound fraction in cells lacking LRIF1^30^. We speculate that loss of chromatin binding increases SMCHD1 protein turnover, accounting for reduced levels of the protein in *Lrif1*^*-/-*^ mESCs (Fig. 2c).

### SMCHD1 exhibits enhanced binding over the Xi territory

**Fig. 3:**
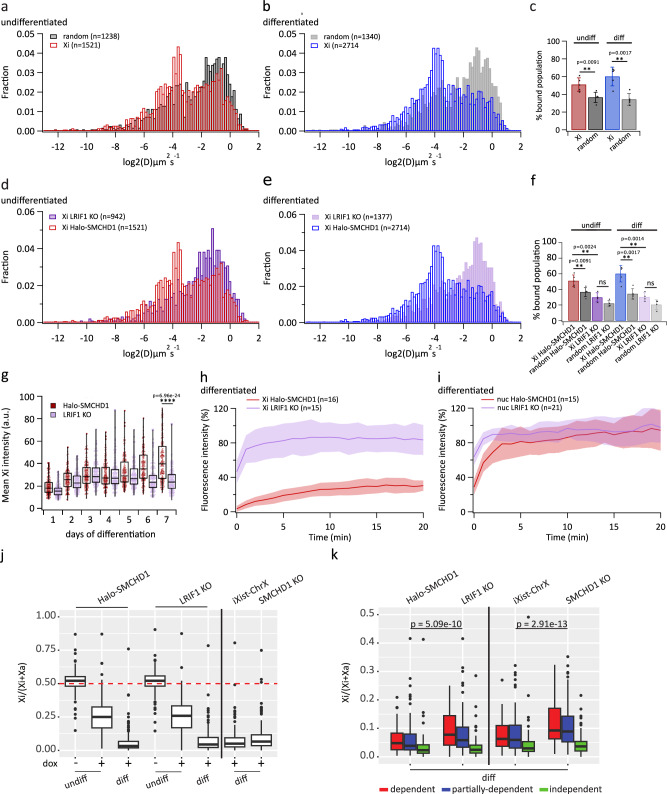
LRIF1 is required for SMCHD1 binding to Xi chromatin in differentiating mESCs. **a** Normalised histogram of diffusion coefficients of tracks from Xi (red) or a random region (black) in undifferentiated Halo-SMCHD1 mESCs treated for 24 h with doxycycline. **b** Normalised histogram of diffusion coefficients of tracks from Xi (blue) or a random region in the nucleus (grey) in Halo-SMCHD1 mESCs differentiated for 7 days. **c** Bar plot showing the average percentage of bound molecules of SMCHD1 (5 independent replicates each). Error bars represent the STDEV (two-sided unpaired t-test). **d** In red, same as in 3a. In dark purple, normalised histogram of diffusion coefficients of tracks from Xi in undifferentiated Lrif1^-/-^ mESCs treated 24 h with doxycycline. **e** In blue, same as in 3b. In light purple, normalised histogram of diffusion coefficients of tracks from Xi in Lrif1^-/-^ mESCs differentiated for 7 days. **f** Bar plot showing the average percentage of bound molecules of SMCHD1 (5 or 4 independent replicates for Halo-SMCHD1 and Lrif1^-/-^ mESCs, respectively). Error bars represent the STDEV (two-sided unpaired t-test). **g** Quantification of SMCHD1 signal underlying CIZ1 domains during a 7-day differentiation timecourse comparing Halo-SMCHD1 (red) and Lrif1^-/-^ (purple) mESCs. Each dot represents a cell, *n* = 136-200. Boxplots: centre lines indicate the median, box limits indicate the first and third quartiles and whiskers extend to min/max data points. Statistical analysis was by two-sided unpaired t-test. **h**, **i** Average fluorescence recovery curves over Xi (**h**) or a random nucleoplasmic region (**i**) comparing Halo-SMCHD1 and Lrif1^-/-^ mESCs differentiated for 7 days. Shading represents the STDEV. **j** Box plot showing allelic X-linked gene (*n* = 184) expression in indicated mESCs with and without doxycycline (dox) and 7 day differentiation. iXist-ChrX is the matched parental line for SMCHD1 KO mESCs. Samples represent one (undiff conditions) or an average of two (diff LRIF1 KO, iXist-ChrX, SMCHD1 KO) or three (diff Halo-SMCHD1) replicates. **k** Box plot showing allelic chrRNA-seq analysis of transcription of X-linked genes (*n* = 184) in indicated cell lines differentiated for 7 days. Genes are split in three categories according to their dependency on SMCHD1 for silencing^13^. The samples represent an average of two (LRIF1 KO, iXist-ChrX, SMCHD1 KO) or three (Halo-SMCHD1) replicates. Statistical analysis was two-sided paired t-test. undiff = undifferentiated, diff = differentiated. Boxplots **j**, **k** centre lines indicate the median, box limits indicate the first and third quartiles and whiskers indicate 1.5× the interquartile range (IQR). Source data are provided as a Source Data file.

We went on to analyse the dynamic behaviour of SMCHD1 in the context of XCI and cellular differentiation. We established an analysis pipeline to define SMCHD1 diffusion behaviour underlying the eGFP-CIZ1-marked location of the Xi domain (Supplementary Fig. 4a, b). As a control, for each analysed Xi territory we used a randomly selected, equal-sized nuclear region (Supplementary Fig. 4b). Using this approach we observed Xi-specific dynamic behaviour of SMCHD1 in both undifferentiated (Fig. 3a, c) and differentiated (Fig. 3b, c) mESCs. In both cases there is a more prominent bound population over the Xi compared to random regions, where SMCHD1 dynamics are, as expected, similar to those observed nucleus-wide (Fig. 2d). Notably, the profile of SMCHD1 diffusion coefficients over the Xi region in differentiated cells strongly resembles that of Halo-tagged histone H2B (Supplementary Fig. 4c). Increased binding of SMCHD1 over the Xi territory was confirmed by fluorescence recovery after photobleaching (FRAP) analysis, which revealed enhanced residency and immobile populations compared to the nucleoplasm (Supplementary Fig. 4d–f).

Previous studies found that LRIF1 depletion in fully differentiated XX somatic cells does not impair enrichment of SMCHD1 over the Xi domain^21,30^, although IF analysis demonstrated that LRIF1 protein does localise to the Xi. To revisit this issue using live-cell imaging we made use of the LRIF1-Halo line described above, which also includes eGFP-CIZ1 integrated into the *Gt(ROSA)26Sor* locus. This analysis indicated strong enrichment of LRIF1-Halo within the Xi territory in 7 day differentiated cells, although not at an earlier stage, 24 h Xist induction (Supplementary Fig. 5a, b). These observations prompted us to investigate if there is a role for LRIF1 in establishing SMCHD1 enrichment at the onset of XCI. Thus, we analysed Halo-SMCHD1 in the *Lrif1*^*-/-*^ mESCs described above. We observed a strong reduction of bound SMCHD1 and a prominent fast diffusing population over the Xi, both in undifferentiated and differentiated cells (Fig. 3d–f). This is accompanied by a clear reduction in Xi associated SMCHD1 (Fig. 3g, and Supplementary Fig. 5c). As for PCH regions, this reduction is not due to lower levels of SMCHD1 in this cell line (Supplementary Fig. 3).

Importantly, the elevated bound population of SMCHD1 on the Xi is lost in the *Lrif1*^*-/-*^ mESCs, with the population distributions within the Xi and a random region being similar, both in undifferentiated and differentiated mESCs (Supplementary Fig. 5d-e, Fig. 3f). These observations were corroborated by FRAP analysis in *Lrif1*^*-/-*^ cells showing SMCHD1 has a similar recovery behaviour within the Xi territory and the nucleoplasm, with a faster and more complete recovery level compared to wild-type cells (Fig. 3h, i, and Supplementary Fig. 5f–i). Consistent with these findings, we observed impaired X-linked gene silencing at day 7 of differentiation in *Lrif1*^*-/-*^ cells, when normally X-linked gene repression is almost complete (Fig. 3j). Impaired silencing was most evident for SMCHD1-dependent and partially dependent genes (as categorised in ref. ^13^) and is comparable to what is observed in *SmcHD1* KO mESCs (Fig. 3k).

### GHKL ATPase mutants define distinct steps of chromatin association

**Fig. 4:**
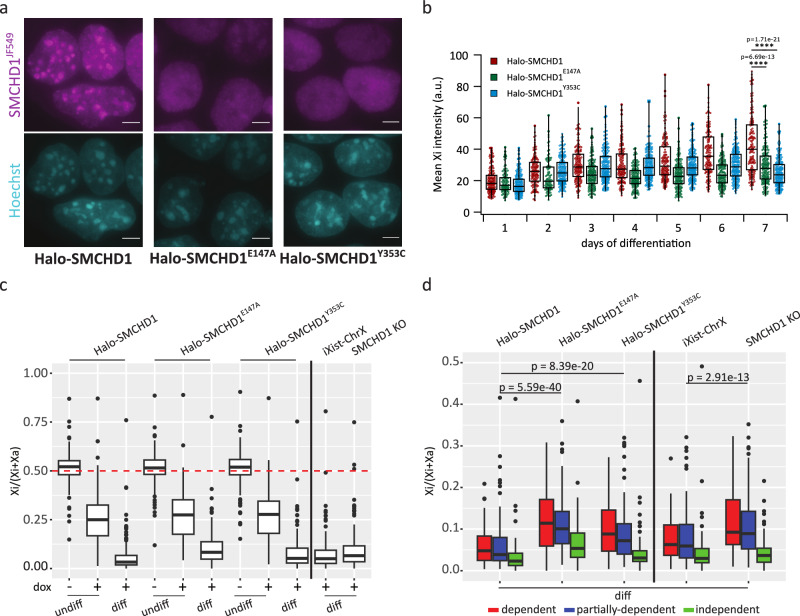
SMCHD1 GHKL ATPase mutations abrogate Xi localisation and silencing function. **a** Representative live-cell images of indicated mESCs undifferentiated, kept for 24 h with doxycyline. Images are maximum-intensity projections. Scale bar − 5 µm. **b** Quantification of SMCHD1 signal underlying CIZ1 domains in indicated cell lines during a 7-day differentiation timecourse. Each dot represents a single cell, *n* = 102–206 (day 7 – Halo-SMCHD1 (151), E147A (185), Y353C (194)). Boxplots: centre lines indicate the median, box limits indicate the first and third quartiles and whiskers extend to min/max data points. Statistical analysis was by two-sided unpaired t-test. **c** Box plot showing allelic ChrRNA-seq analysis of transcription of X-linked genes (*n* = 184) in indicated cell lines with and without doxycycline (dox) and 7 day differentiation. The boxes represent one (undiff conditions) or an average from two (diff E147A, Y353C, iXist-ChrX, SMCHD1 KO) or three (Halo-SMCHD1) replicates **d** Box plot showing allelic ChrRNA-seq analysis of transcription of X-linked genes (*n* = 184) in mESC lines which were differentiated for 7 days, with genes split into three categories according to their dependency on SMCHD1 for silencing^13^. An average of two (E147A, Y353C, iXist-ChrX, SMCHD1 KO) or three (Halo-SMCHD1) replicates was used for this plot. Statistical analysis – two-sided paired t test. undiff = undifferentiated, diff = differentiated. Boxplots (**c**, **d**) centre lines indicate the median, box limits indicate the first and third quartiles and whiskers indicate 1.5× the interquartile range (IQR). Source data are provided as a Source Data file.

In previous work, we reported that E147A mutation of the SMCHD1 GHKL ATPase domain abrogates Xi accumulation in XX somatic cells^30^. In vitro analysis has shown that E147A completely abolishes ATP hydrolysis, although ATP is still able to bind^30,35^. We set out to investigate how this mutation affects the dynamic behaviour of SMCHD1 on the Xi and genome-wide. In parallel we tested a second mutation Y353C, identified in FSHD2 patients, that has been shown to strongly abrogate SMCHD1 ATPase activity without affecting folding/protein stability^28,31^. Using CRISPR/Cas9-mediated homologous recombination, we introduced each of these mutations, biallelically at the endogenous *SMCHD1* locus in the wild-type cell line, Halo-SMCHD1 (Supplementary Fig. 6a, b). Western blot analysis indicated that both mutations result in a small reduction in levels of SMCHD1 (Supplementary Fig. 6c, d). Both mutant proteins show loss of enrichment at PCH regions, as seen in *Lrif1*^*-/-*^ cells (Fig. 4a), and over the Xi during a 7-day differentiation time-course (Fig. 4b, and Supplementary Fig. 6e, f). Consistent with these observations, ChrRNA-seq revealed that SMCHD1^E147A^ and SMCHD1^Y353C^ show abrogated Xi silencing (Fig. 4c), particularly affecting SMCHD1-dependent genes and partially-dependent genes (Fig. 4d). Abrogated silencing was equivalent to that seen in *Smchd1*^-/-^ XX mESCs (Fig. 4c, d).

**Fig. 5:**
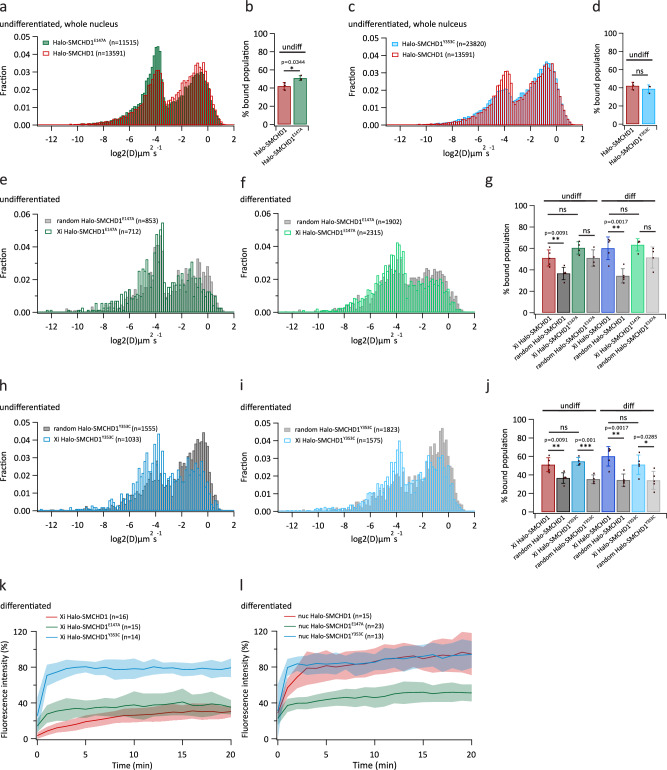
SMCHD1 GHKL ATPase loss-of-function mutations alter chromatin binding behaviour. **a** In red, same as Fig. 2d. In green, normalised histogram of diffusion coefficients of tracks from across the whole nucleus in undifferentiated Halo-SMCHD1^E147A^ mESCs, kept for 24 h with doxycycline. **b** Bar plots showing the average percentage of bound molecules of SMCHD1 (3 independent replicates each). Error bars represent the STDEV (two-sided unpaired t-test). **c** In red, same as Fig. 2d. In turquoise, normalised histogram of diffusion coefficients of tracks from across the whole nucleus in undifferentiated Halo-SMCHD1^Y353C^ mESCs, kept for 24 h with doxycycline. **d** Bar plots showing the average percentage of bound molecules of SMCHD1 (3 independent replicates each). Error bars represent the STDEV (two-sided unpaired t-test). **e** Normalised histogram of diffusion coefficients of tracks from Xi (dark green) or a random region (dark grey) in undifferentiated Halo-SMCHD1^E147A^ mESCs, kept for 24 h with doxycycline. **f** Normalised histogram of diffusion coefficients of analysed tracks from Xi (light green) or a random region (light grey) in Halo-SMCHD1^E147A^ mESC differentiated for 7 days. **g** Bar plot showing the average percentage of bound molecules of SMCHD1 (5 or 4 independent replicates for Halo-SMCHD1 and Halo-SMCHD1^E147A^, respectively) mESCs. Error bars represent the STDEV (two-sided unpaired t-test). **h** Normalised histogram of diffusion coefficients of tracks from the Xi (turquoise) or a random region (dark grey) in undifferentiated Halo-SMCHD1^Y353C^ mESCs, kept for 24 h with doxycycline. **i** Normalised histogram of diffusion coefficients of tracks from Xi (light blue) or a random region (light grey) in Halo-SMCHD1^Y353C^ mESCs differentiated for 7 days. **j** Bar plot showing the average percentage of bound molecules of SMCHD1 (5 independent replicates each). Error bars represent the STDEV (two-sided unpaired t-test). **k**, **l** Average fluorescence recovery curves over the Xi domain (**k**) or a random nucleoplasmic region (**l**) in Halo-SMCHD1, Halo-SMCHD1^E147A^ and Halo-SMCHD1^Y353C^ mESCs differentiated for 7 days. The shading represents the STDEV. undiff = undifferentiated, diff = differentiated. Source data are provided as a Source Data file.

We went on to analyse the dynamic behaviour of the mutant proteins using SPT (Fig. 5a–j, and Supplementary Fig.7a–h) and FRAP (Fig. 5k, l, Supplementary Fig.8a-e). SPT analysis revealed that SMCHD1^E147A^ has a larger bound population compared to the wild-type protein, both in undifferentiated and differentiated cells (Fig. 5a, b, and Supplementary Fig. 7a, b). SMCHD1^Y353C^ on the other hand has a distribution profile similar to wild-type (Fig. 5c, d, and Supplementary Fig. 7c, d). These results highlight an interesting contrast between the *Lrif1*^*-/-*^, SMCHD1^E147A^ and SMCHD1^Y353C^ cells, all of which show loss of PCH accumulation of SMCHD1, whilst exhibiting different dynamic characteristics.

Looking at the effect of the introduced mutations on SMCHD1 dynamic behaviour across the Xi domain, for SMCHD1^E147A^, we observed little difference between the population distribution profiles of the mutant and wild-type proteins over the Xi, both in undifferentiated and differentiated cells (Supplementary Fig. 7e, f). This may seem counterintuitive given the absence of enrichment of the mutant protein over the Xi (Fig. 4b, and Supplementary Fig. 6e), but it is key to note that the bound population of SMCHD1^E147A^ in random regions is very similar to that seen at the Xi, both in undifferentiated and differentiated cells (Fig. 5e–g). These observations imply that the mutation retains the ability to engage with chromatin but that there is no specific preferred association and, consequently, accumulation at target sites (Xi and PCH). Consistently, FRAP analysis revealed a highly similar recovery behaviour for SMCHD1^E147A^ within the Xi domain and the nucleoplasm, with the Xi immobile fraction being equivalent to wild-type protein, but with a markedly reduced residence time (Fig. 5k, l, Supplementary Fig. 8a, c–e).

The SMCHD1^Y353C^ mutation resulted in very different changes in dynamic behaviour relative to SMCHD1^E147A^. Binding over the Xi and random region is unchanged compared to wild-type, both in undifferentiated and differentiated cells and, hence, the mutant protein continues to associate preferentially with Xi (Fig. 5h–j, Supplementary Fig. 7g, h). However, in FRAP experiments SMCHD1^Y353C^ recovers to almost pre-bleach levels within the Xi domain and the nucleoplasm, and, importantly, has a much faster turnover compared to the wild-type protein (Fig. 5k, l, and Supplementary Fig. 8b–e). This striking decrease in residency on chromatin likely underlies the changes in localisation of SMCHD1^Y353C^ at PCH and Xi, given that the population distribution profile assessed by SPT is unchanged.

### Gain-of-function GHKL ATPase mutations enhance Xi recruitment and silencing

**Fig. 6:**
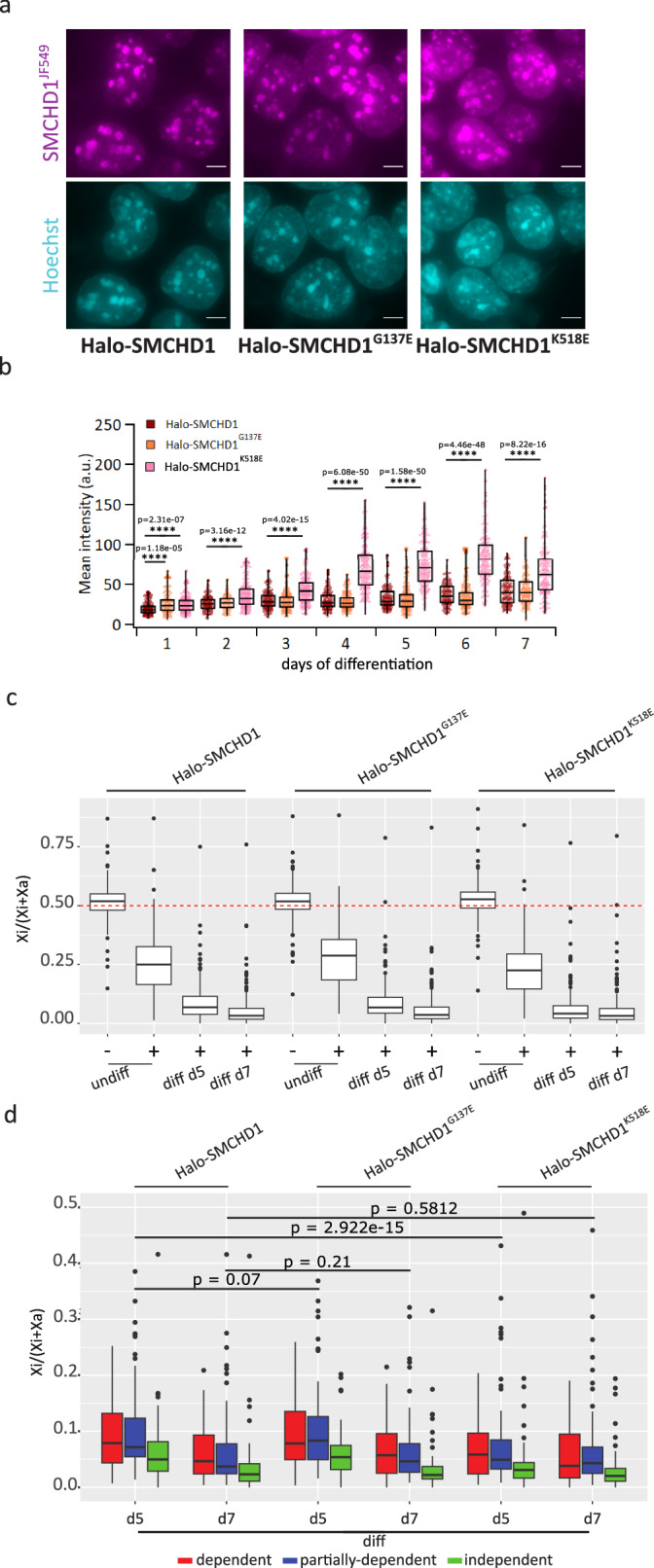
Gain-of-function mutations in the SMCHD1 GHKL ATPase can enhance Xi recruitment and X-linked gene silencing. **a** Representative live-cell images of indicated mESCs undifferentiated, kept for 24 h with doxycycline. Images are maximum-intensity projections, scale bar − 5 µm. **b** Quantification of SMCHD1 signal underlying CIZ1 domains in indicated cell lines during 7-day differentiation timecourse. Each dot represents a cell, *n* = 83-210 (Halo-SMCHD1 (159, 136, 148, 145, 148, 141, 151), K518E (180, 210, 198, 209, 207, 200, 200) for day 1-7, G137E (109) for day 1). Boxplots: centre lines indicate the median, box limits indicate the first and third quartiles and whiskers extend to min/max data points. Statistical analysis was by two-sided unpaired t-test. **c** Box plot showing allelic gene expression for X-linked genes (*n* = 181) in indicated cell lines; uninduced, induced for 24 h with doxycycline or differentiated for 5 or 7 days. The boxes represent one (undiff conditions) or an average of two (Halo-SMCHD1 d5, G137E d5, K518E d5 and d7) or three (Halo-SMCHD1 d7, G137E d7) replicates **d** Box plot showing allelic X-linked gene (*n* = 181) expression in indicated cell lines differentiated for 5 or 7 days, with genes split in three categories according to their dependency on SMCHD1 for silencing^13^. The boxes represent an average of two (Halo-SMCHD1 d5, G137E d5, K518E d5 and d7) or three (Halo-SMCHD1 d7, G137E d7) replicates. Statistical analysis – two-sided paired t test. Boxplots (**c**, **d**) centre lines indicate the median, box limits indicate the first and third quartiles and whiskers indicate 1.5× the interquartile range (IQR). Source data are provided as a Source Data file.

We went on to investigate additional mutations in the SMCHD1 GHKL ATPase domain, G137E and K518E, both of which result in increased ATPase activity in vitro^28,35^. It is worth noting that, as for the loss-of-function mutants, the ATPase activity has been assessed using constructs that contain only the ATPase domain, so the extent to which these mutations affect the hydrolysis reaction in the context of the full-length protein may differ. G137E was identified in a BAMS patient and an FSHD2 patient and has been suggested to give rise to a gain-of-function phenotype^32,33^. K518E was found uniquely in association with BAMS^31^. We introduced each mutation biallelically at the native *SmcHD1* locus in the wild-type cell line, Halo-SMCHD1, as described for E147A and Y353C (Supplementary Fig. 9a, b). The mutations did not significantly alter SMCHD1 protein levels (Supplementary Fig. 9c, d), and, in contrast to the loss-of-function mutations, G137E and K518E accumulated at PCH (Fig. 6a). Similarly, localisation to Xi during cellular differentiation is retained in both cases (Supplementary Fig. 9e, f). Interestingly, quantitative analysis of Xi-associated SMCHD1 intensity revealed SMCHD1^G137E^ is increased relative to wild-type 1 day following Xist induction, whilst SMCHD1^K518E^ shows a clear increase across the entire differentiation timecourse (Fig. 6b, and Supplementary Fig. 9f). Consistent with these observations K518E results in markedly accelerated X-linked gene silencing, with no measurable effect in the case of G137E (Fig. 6c, d).

**Fig. 7:**
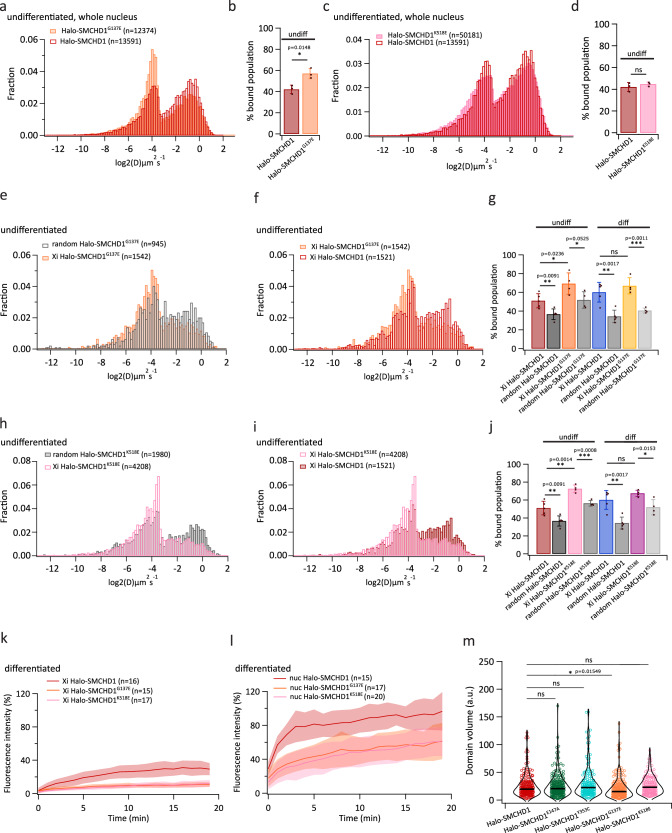
SMCHD1 GHKL ATPase gain-of-function mutations alter chromatin binding behaviour. **a** In red, same as Fig. 2d. In orange, normalised histogram of diffusion coefficients of tracks from across the whole nucleus in undifferentiated Halo-SMCHD1^G137E^ mESCs, kept for 24 h with doxycycline. **b** Bar plots showing the average percentage of bound molecules of SMCHD1 (3 independent replicates each). Error bars represent the STDEV (two-sided unpaired t-test). **c** In red, same as Fig. 2d. In pink, normalised histogram of diffusion coefficients of tracks from across the whole nucleus in undifferentiated Halo-SMCHD1^K518E^ mESCs, kept for 24 h with doxycycline. **d** Bar plots showing the average percentage of bound molecules of SMCHD1 (3 independent replicates each). Error bars represent the STDEV (two-sided unpaired t-test). **e** Normalised histogram of diffusion coefficients of tracks over Xi (orange) or a random region (dark grey) in undifferentiated Halo-SMCHD1^G137E^ mESCs, kept for 24 h with doxycycline. **f** In red, same as in 3a. In orange, same as in 7e. **g** Bar plot showing the average percentage of bound molecules of SMCHD1 (5 or 4 independent replicates for Halo-SMCHD1 and Halo-SMCHD1^G137E^ mESCs, respectively). Error bars represent the STDEV (two-sided unpaired t-test). **h** Normalised histogram of diffusion coefficients of tracks over Xi (pink) or a random region (dark grey) in undifferentiated Halo-SMCHD1^K518E^ mESCs, kept for 24 h with doxycycline. **i** In red, same as in 3a. In pink, same as in 7 h. **j** Bar plot showing the average percentage of bound molecules of SMCHD1 (5 or 4 independent replicates for Halo-SMCHD1 and Halo-SMCHD1^K518E^ mESCs, respectively). Error bars represent the STDEV (two-sided unpaired t-test). **k**, **l** Average fluorescence recovery curves over the Xi domain (**k**) or a random nucleoplasmic region (**l**) in indicated cell lines differentiated for 7 days. The shading represents the STDEV. **m** Violin plot showing the volume of the H3K27me3 domain in the indicated cell lines differentiated for 7 days. Each dot represents a cell. *n* = 207 (Halo-SMCHD1), 239 (Halo-SMCHD1^E147A^), 177 (Halo-SMCHD1^Y353C^), 258 (Halo-SMCHD1^G137E^), 174 (Halo-SMCHD1^K518E^) (two-sided unpaired t-test). undiff = undifferentiated, diff = differentiated. Source data are provided as a Source Data file.

In line with enhanced functionality in XCI, analysis of dynamic behaviour in undifferentiated and differentiated cells using SPT (Fig. 7a–j, and Supplementary Fig. 10a–h) and FRAP (Fig. 7k, l, and Supplementary Fig. 11a–e) demonstrates enhanced chromatin association of both mutant proteins, albeit with some interesting differences. Thus, SPT analysis shows that across the nucleus, SMCHD1^G137E^ but not SMCHD1^K518E^ has an increased bound population (Fig. 7a–d, and Supplementary Fig. 10a-d). Over Xi domains, both mutant proteins have larger and more prominent bound populations in undifferentiated cells (Fig. 7e–j), in accordance with the observed increases in Xi accumulation (Fig. 6b). A small increase in the bound proportion in differentiated cells is not statistically significant (Fig. 7g,j, Supplementary Fig. 10e-h). In FRAP experiments, SMCHD1^G137E^ and SMCHD1^K518E^ recover more slowly and to a lesser extent compared to wild-type SMCHD1, both in the Xi territory and the nucleoplasm (Fig. 7k, l, Supplementary Fig. 11c–e). Importantly, the difference in dynamic behaviour between the two regions observed in wild-type cells is retained.

SMCHD1 has been reported to promote Xi compaction in human cells^21^, and in a final series of experiments, we tested whether the different ATPase domain mutations affect Xi volume. To achieve this, we used the Xi-associated histone modification H3K27me3 as a proxy for Xi territory volume, as described previously^49^. No differences in Xi volume were observed for the ATPase loss-of-function mutations SMCHD1^E147A^ and SMCHD1^Y353C^, nor for the gain-of-function mutation SMCHD1^K518E^. However, there was a significant reduction in Xi volume at day 7 of differentiation with SMCHD1^G137E^ (Fig. 7m and Supplementary Fig. 11f). This result links ATPase activity and Xi compaction, which contextualises the important role for SMCHD1 in XCI.

## Discussion

Our newly developed XX mESC model for live-cell imaging and SPT during establishment of XCI has provided valuable insights into SMCHD1 target site selectivity and its functional interaction with chromatin, including the demonstration that SMCHD1 associates with PCH domains in most cells, and the critical role of LRIF1 in chromatin loading, at both PCH and the Xi. The observation that the majority of SMCHD1 molecules are diffusive in the *Lrif1*^-/-^ cells, unlike in wild-type cells where almost half are chromatin-bound, suggests that the nuclear-wide bound population corresponds in large part to SMCHD1 interacting with H3K9me3-marked heterochromatic regions. H3K9me2-marked heterochromatin may also be a target, given that the HP1 chromodomain also binds to this histone mark^50,51^. Whilst LRIF1 function accounts for the majority of bound SMCHD1 in mESCs, some PCH domains retain SMCHD1 enrichment in a small proportion of *Lrif1*^*-/-*^ cells, indicating an alternative mechanism for PCH localisation. The basis for this effect is not known, but it could indicate a pathway that functions at a specific cell cycle stage or a second adaptor protein that is present only in a small subset of cells.

Involvement of LRIF1 in SMCHD1 recruitment to Xi was surprising, given prior evidence that LRIF1 is not required to maintain SMCHD1 on Xi in differentiated somatic cells^21,30^, and moreover, evidence demonstrating a critical role for PRC1-mediated H2AK119ub1 and progression of cellular differentiation for Xi recruitment^11,23^. LRIF1, in contrast, functions as an adaptor linking SMCHD1 to H3K9me3-modified chromatin^21,30^. Accordingly, we speculate that a baseline level of chromatin-bound SMCHD1 recruited via LRIF1-mediated recognition of H3K9me3 at constitutive heterochromatic regions (PCH and interstitial chromosome arm regions) facilitates stable accumulation of additional SMCHD1 driven by PRC1-mediated H2AK119ub1. This could result from interaction between prebound and newly recruited SMCHD1, or alternatively, reflect an indirect effect resulting from the activity of pre-bound SMCHD1. This interpretation also helps to explain our finding using live-cell imaging that Xi loading of SMCHD1 occurs at the outset of XCI, and as a progressive continuum thereafter, rather than at a specific differentiation stage as previously thought^10,11^.

**Fig. 8:**
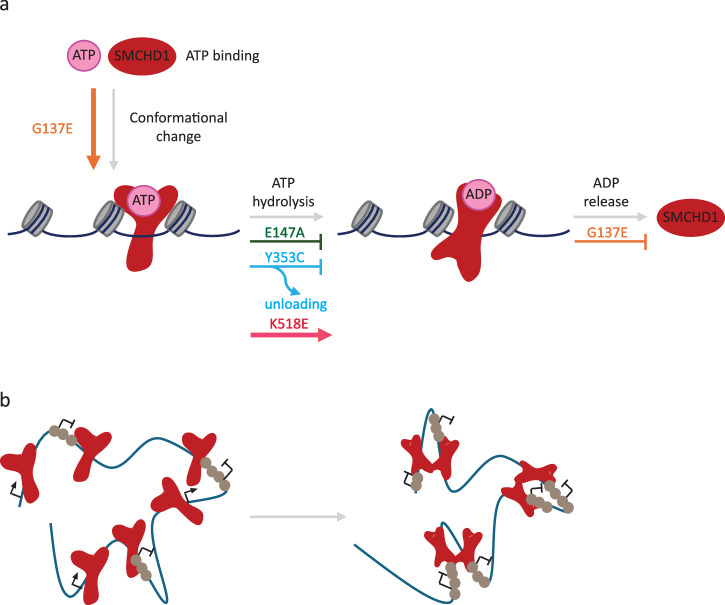
Models for the role of SMCHD1 GHKL ATPase in governing selective chromatin interactions. **a** The model invokes that ATP binding to SMCHD1 leads to a conformational change that includes dimerisation of the N-terminal GHKL ATPase domains, favours ATP hydrolysis and enables initial loading onto chromatin. Subsequently, ATP hydrolysis takes places, leading to a new protein conformation which favours stable and specific association with chromatin and, potentially, allows distant DNA-bound SMCHD1 molecules to associate with one another (as shown in **b**), forming long range chromatin interactions and selective enrichment at specific chromatin regions. Once ADP is released, SMCHD1 unloads from chromatin. The G137E mutation favours ATP binding and, hence, SMCHD1 loading onto chromatin and may also disfavour ADP release and, thus, SMCHD1 dissociation from chromatin. The K518E mutation favours ATP hydrolysis and, hence, stable engagement with chromatin. The E147A mutation prevents ATP hydrolysis and locks the protein in the less stably chromatin associated ATP-bound state. The Y353C mutation blocks the catalytic reaction and causes SMCHD1 to unload from chromatin. **b** In the context of the Xi, the formation of SMCHD1 mediated long-range chromatin interactions via cycles of ATP binding and hydrolysis may enable spreading of heterochromatic epigenetic marks and silencing factors and, consequently, gene silencing.

It has been proposed that SMCHD1 functions similarly to other GHKL ATPases via a molecular clamp mechanism mediated by the ATP binding-hydrolysis-release cycle^9,35,36^. In vitro analysis has shown SMCHD1 has a weak ATPase activity, which is probably only able to drive conformational changes within the protein itself, as in other GHKL ATPases, rather than power a motor function such as the cohesin-driven loop extrusion process^36,52^. With these considerations in mind, together with our analysis of the effects of different mutations in GHKL ATPase domain, we propose that there are sequential chromatin engagement and consolidation steps for SMCHD1 loading and function at target sites (Fig. 8a). In this model, chromatin engagement occurs as a result of ATP binding, whilst consolidation is the consequence of conformational changes/head engagement resulting from ATP hydrolysis. Such a conformational change may allow the chromatin-bound protein homodimer to simultaneously bind to a different genomic location and, thus, form long-range chromatin interactions. Consequently, different genomic locations may be brought into close proximity (Fig. 8b). Preferential loading at target loci (e.g., PCH and Xi) is suggested to occur as a result of the probabilistic increase in chromatin proximal SMCHD1 linked to interactions with specific chromatin features (e.g., H3K9me3), and/or loading factors (e.g., LRIF1) promoting the consolidation step.

In the context of the aforementioned model we interpret the effect of different GHKL ATPase mutations as follows: The E147A mutation, which affects a core catalytic residue such that the mutant can bind but not hydrolyse ATP^30,35^ (Supplementary Fig. 12a-c), is held in a state that allows engagement but prohibits the consolidation step required for target site accumulation and downstream functions, evidenced as reduced residency on chromatin. Increased chromatin binding of the engagement locked SMCHD1^E147A^ molecules at sites genome-wide may also be a factor in the loss of target specificity. Our analysis of the Y353C mutation, which also abrogates ATPase activity^28,31^, indicates a more marked effect than SMCHD1^E147A^ in terms of loss of stable chromatin association. A possible explanation is that the conformational change induced by ATP binding results in the mutant protein disengaging from chromatin rapidly following initial binding. Y353 is a large residue which forms multiple contacts within the ATPase domain (Supplementary Fig. 12d). Substitution to cysteine may affect the binding kinetics of ATP and/or the wider conformation changes associated with ATP binding, precluding ATP hydrolysis. The gain-of-function mutations G137E and K518E result in increased ADP production^28^. G137E is also characterised by enhanced dimerisation capacity in vitro^35^, probably due to formation of new salt bridge interactions at the dimer interface (Supplementary Fig. 12e), suggesting the mutation may facilitate a default conformation which favours ATP binding. This consideration could explain the increased bound population of SMCHD1 that we observe, both genome-wide and at target sites. Bound SMCHD1^G137E^ has an enhanced residence time on chromatin, potentially due to the G137E-induced dimer conformation requiring more energy to unfold and, thus, disfavouring ADP release and consequently SMCHD1 unloading. SMCHD1^G137E^ is uniquely associated with greater compaction of Xi chromatin. We suggest that this outcome could result from an increased frequency of bound sites, which then associate with one another to induce compaction. K518E promotes ATP hydrolysis, probably by stabilising the active site (Supplementary Fig. 12f) and, hence, the consolidation step of chromatin association. We infer that this results in a highly stable conformation which favours increased accumulation, residence and functionality of SMCHD1^K518E^ at target sites. In the case of Xi we suggest these features account for the accelerated rate of X-linked gene silencing that we observe. It should be noted that whilst we infer that the ATPase mutants directly affect interaction between SMCHD1 and chromatin, we cannot rule out a contribution to interactions with LRIF1 or other unknown proteins.

In summary, our findings highlight the key role played by the GHKL ATPase domain for chromatin association and functionality of SMCHD1. In the future, analysis of other mutants coupled to in vitro structural and functional investigations of full-length SMCHD1 should provide a more comprehensive understanding. This is key not only to elucidating SMCHD1 function in XCI, but also to understanding and developing therapeutic strategies for BAMS and FSHD2 patients.

## Methods

### Cell lines

All mouse embryonic stem cells (mESCs) were grown at 37 °C, 5% CO_2_ in ES medium: Dulbecco’s Modified Eagle Medium (DMEM; Life Technologies) supplemented with 10% Foetal Calf Serum (ThermoFisher), 0.1 mM non-essential amino acids, 2mM L-glutamine, 50 μM β-mercaptoethanol, 100 U/ml Penicillin/100 μg/ml Streptomycin (all from Life Technologies) and 1000 U/mL Leukaemia Inhibitory Factor (LIF) (made in-house). The cells were grown on gelatinised plates in feeder-dependent conditions. Feeders were generated from mitomycin-inactivated (Sigma Aldrich) SNLPs (STO mouse fibroblasts expressing Neomycin, Puromycin resistance and Lif genes). The main parental cell line for all subsequently derived cell lines is the interspecific (129/sv - Cast/Ei) iXist-ChrX XX mESC line previously derived and described in ref. ^38^. Briefly, these cells include a tetracyline-inducible promoter (TetON) on the *M.m. domesticus* (129/sv) Xist allele and a reverse tetracycline-controlled transactivator (rtTA) expressed from the *Tigre* locus. For Xist induction, cells were treated with 1 μg/ml doxycycline (Sigma).

### Cloning of plasmids for CRISPR-Cas9 targeting

For the homology vectors (HVs), homology arms of ~500–600 bp flanking the insertion site were amplified by PCR from mESC genomic DNA using Fast-Start High Fidelity enzyme (Merk Life Science). The Halo-, Snap- and eGFP-tags were amplified from previously generated vectors containing these sequences. The fragments were then ligated together with the backbone plasmid by Gibson Assembly ligation using Gibson Assembly Master Mix (NEB) according to the manufacturer’s guidance. For most HVs, the primers for Gibson Assembly were designed so that they introduced mutations to the PAM recognition sequence of the corresponding sgRNAs and/or mutations to create restriction enzyme (RE) sites required for screening by PCR followed by RE digestion. When introduction of such mutations was not possible through the Gibson Assembly primers, the plasmids obtained were subsequently mutagenized using QuikChange Lightning Site-Directed Mutagenesis kit (Agilent).

Selection of the sgRNAs required for recognition and subsequent double-strand break generation at the target loci by CRISPR-Cas9 was done using the online tool CRISPOR^53^. Annealed dsDNA oligos matching the chosen sgRNA were cloned into the PX459 V2.0 backbone (Addgene plasmid #62988) following the single-step digestion-ligation Zhang lab protocol^54^. The sgRNA sequences used are listed in Table S1.

The ligated products were transformed into XL10-Gold ultracompetent bacteria (Agilent) and DNA was isolated from single bacterial colonies using the Miniprep kit (Qiagen). The plasmid sequences were checked via Sanger sequencing (SS) (Source BioScience).

### Generation of stable cell lines

A day before transfection, 250,000 cells/well were seeded in 6-well plates on feeders. Cells were transfected using Lipofectamine 2000 (ThermoFisher) according to the manufacturer’s protocol. The HVs were co-transfected with the Cas9-sgRNA plasmids at a molar ratio of 6:1 (2.4 µg HV and ~1 µg Cas9-sgRNA plasmid). For the LRIF1 KO cell line, two Cas9-sgRNA plasmids targeting exons 1 and 4 were transfected, each at 1 µg. Transfected cells were passaged 24 h post-transfection onto gelatinised 90 mm Petri dishes with feeders and, 48 h post-transfection, puromycin (3–5 µg/ml) selection was applied. Cells were kept under selection for 48 h followed by exchanging for fresh mESC media every day for a further 7–9 days until individual colonies were picked. The clones were then expanded in 96-well plates for screening. A summary of the cell lines generated is shown in Table S2 and a list of the primers used for screening is shown in Table S3.

### Monolayer (ML) differentiation

For in vitro differentiation, the cells were pre-plated for 30−40 min in non-gelatinised plates to remove feeder cells, due to feeders faster attachment relative to mESCs. The collected cells in suspension, which are primarily mESCs, were counted and plated at a density of 2000 cells/cm^2^ in gelatinised plates. The differentiating cells were grown for up to 7 days. From the pre-plating step onwards, cells were kept in EC10 + dox medium: Dulbecco’s Modified Eagle Medium (DMEM; Life Technologies) supplemented with 10% Fetal Calf Serum (ThermoFisher), 0.1 mM non-essential amino acids, 2mM L-glutamine, 50 μM β-mercaptoethanol, 100 U/ml Penicillin/100 μg/ml Streptomycin (all from Life Technologies) and 1 μg/ml doxycycline (Sigma).

### Single particle tracking (SPT)

Cells were plated on gelatinised 35 mm µ-dishes containing a #1.5 polymer coverslip (Ibidi) two days before imaging in the case of differentiated cells or one day before imaging for undifferentiated cells. In the latter case, after plating, 1 μg/ml doxycycline was added to the dish, and the cells were imaged 24 h later. Halo-PA-JF549 ligand (HHMI Janelia)^55^ was used for labelling at 100 nM for 15 min at 37 °C. Cells were then washed twice with PBS (Sigma) and incubated for 30 min in Fluorobrite DMEM (ThermoFisher) supplemented with 10% Fetal Calf Serum (ThermoFisher) and 1 μg/ml doxycycline. After three more washes with PBS, cells were imaged in Fluorobrite DMEM supplement as above and additionally with 30 mM HEPES pH 7.4 to maintain the pH of the sample medium.

SPT experiments were carried out with a custom TIRF/HILO microscope, described previously^56^, with an EMCCD camera (Andor), laser module (iChrome MLE MultiLaser engine, Toptica Photonics), 100×1.4NA objective (Olympus) and translational module (ASI), which allows for adjustment of the angle of the excitation beam between epi- and HILO-illumination to achieve a high signal-to-noise ratio.

To visualise both diffusing and bound molecules, movies of 4000 frames, with 15 ms/frame, were acquired with continuous 561 nm laser excitation at 22 mW intensity and 405 nm laser excitation at varied intensities to maintain a low density of fluorescent particles. Importantly, for the Xi-focused acquisitions, an image of the Xi region(s) was taken before and after the movie acquisition with 488 nm laser excitation at 2 mW and 20 ms camera exposure. For whole nucleus datasets, at least 20 movies were taken, each containing about three cells, and at least three replicates were performed for each timepoint studied. For Xi-focused datasets, 50 movies were taken, each containing at least one Xi domain, and at least four replicates were performed for each timepoint studied. The precise total number of movies/Xi/random regions analysed for each timepoint is shown in Table S4.

### SPT data analysis

Single particle tracking datasets were analysed predominantly in MATLAB (MathWorks) using Stormtracker software^57^, unless otherwise specified. Localisation of single particles in each frame of the acquired movies was achieved with a precision of 25 nm by fitting an elliptical Gaussian point spread function to the detected molecules. The detection of the molecules was done based on an intensity threshold fixed for all movies taken within the same acquisition session but adjusted as needed from session to session after manually checking the detection efficiency. Once localised, the molecules found within a radius of 768 nm in consecutive frames of the movie were linked to form tracks; single frame gaps were allowed to take into account for spontaneous fluorophore blinking. The tracks which contained a minimum of four frames were then used to calculate their apparent diffusion coefficient D^*^.

1 $${D}^{*}={MSD}/(4\triangle t)-{\sigma }_{{loc}}^{2}/\triangle t$$

where Δt = 15.26 msec, σ_loc_ = 40 nm and MSD is the mean-squared displacement:

2 $${{{\rm{MSD}}}}=1/(N-1){\sum}_{i=1}^{N-1}{({x}_{i+1}-{x}_{i})}^{2}+{({y}_{i+1}-{y}_{i})}^{2}$$

where N ≥ 5, as only tracks with at least four frames are considered.

The log2 values corresponding to all the tracks found across all movies and all replicates for each timepoint analysed were displayed as a histogram using Igor Pro 9 (WaveMetrics); the histograms were normalised to the number of tracks analysed in each timepoint.

Tracks with a log2(D*) smaller than -3 were considered to represent bound molecules, and the bound population was calculated as a percentage of bound tracks relative to the total analysed tracks for that timepoint. For Supplementary Fig. 2f, Gaussian fitting and SPOT-ON kinetic modelling were done as previously described^47,48^.

For the Xi-focused datasets, a filtering step was added to attribute the localised molecules found within the Xi territory prior to linking these localised molecules into tracks. To do this, firstly, the two images of the Xi region, taken before and after the movie acquisition, were merged using ImageJ/FiJi. If the region was found to have significantly moved during the acquisition, that particular field of view was discarded from further analysis. A custom ImageJ/FiJi script was used to manually select the Xi region in each of the merged images, as well as a random region of the same size as the Xi region but drawn somewhere else in the nucleus. The random region can include PCH regions, as their location is unknown in this setup. The script outputs files containing the xy coordinates of these regions. These files were inputted in a second custom-made R script used to filter the localised single molecules, keeping only the molecules present in each of the selected regions. The filtered molecules were then merged into tracks, which were analysed as described above.

### Live cell imaging

Cells were plated in gelatinised µ-slide 8 wells or 35 mm µ-dishes with a polymer coverslip (Ibidi) at least four hours before imaging. Before imaging, cells were labelled with Halo-JF549 ligand (Promega) at 100 nM for 30 min at 37 °C. Cells were then incubated for 30 min in growing media and subsequently washed twice with PBS (Sigma). Cells were imaged in Fluorobrite DMEM (ThermoFisher) supplemented with 10% Fetal Calf Serum (ThermoFisher) and, if needed, 1 μg/ml doxycycline and/or 0.1 µg/ml Hoechst (ThermoFisher). If needed, prior to labelling with Halo-JF519, cells were treated with HaloPROTAC-E (Aobious) at the required concentration (diluted in growing media) for 1.5 h at 37 °C. Cells were then labelled with Halo-JF549 (Promega) at 100 nM for 15 min at 37 °C, washed twice with PBS (Sigma) and incubated for 30 min in growing media supplemented with HaloPROTAC-E (Aobious) at the required concentration. After two washes with PBS (Sigma), cells were imaged in Fluorobrite DMEM (ThermoFisher) supplemented with 10% Fetal Calf Serum (ThermoFisher) and HaloPROTAC-E (Aobious) at the required concentration.

Live cell imaging was performed using an Olympus IX83 microscope equipped with a 63 × 1.4NA objective, a 1200 × 1200 px^2^ sCMOS camera (Photometrics 95B) and a humidified chamber with carbon dioxide atmosphere at 37°C. The microscope was operated via CellSens software. When needed, an additional magnifying 1.6x or 2x lens was used in front of the camera leading to a final pixel size of 114.4 nm or 91.5 nm, respectively. z-stacks were captured every 0.5 µm, using 10–250 ms exposure at 3-50% laser power depending on the channel. All acquired images were analysed and processed using ImageJ/Fiji.

### Time course setup and analysis

For live imaging of the 7-day ML differentiation timecourse, all analysed cells were seeded for differentiation at the same time. Each day of the timecourse, cells were plated on 8 well µ-slides with a polymer coverslip (Ibidi) four hours before imaging. The images were acquired with the Olympus IX83 microscope using the 2x lens in front of the camera. The analysis was performed in ImageJ/FiJi using maximum-intensity projections of the acquired images. A binary mask of the Xi domains was created by thresholding the green channel images representing eGFP-CIZ1 signal. Each red channel image representing Halo-JF549-SMCHD1 signal was multiplied by its corresponding binary mask and then the Analyse Particles function was used to determine the mean SMCHD1 signal intensity in each analysed Xi domain. The values for each day were plotted as a box plot using Igor Pro 9.

### Fluorescence recovery after photobleaching (FRAP)

Differentiating cells were plated on gelatinised 35 mm µ-dishes with a polymer coverslip (Ibidi) two days before imaging. Trolox 1 mM (Sigma-Aldrich) was added to the media 24 h before imaging to reduce phototoxicity as a result of prolonged imaging. Before imaging, cells were labelled as described above with Halo-JF549 ligand and imaged in Fluorobrite DMEM supplemented with 10% Fetal Calf Serum and 1 µg/ml doxycycline. Live-cell time-course imaging was performed using an Olympus SoRa spinning disc confocal equipped with a 63×1.4NA objective, sCMOS camera (Photometrics), 405 nm FRAP module and a humidified chamber with carbon dioxide atmosphere at 37 °C. The microscope was operated using the CellSens software. The ROIs, either Xi or nucleoplasmic regions, were manually selected using the two-point circle measuring tool and photobleached with one bleach pulse of 40% laser intensity, following acquisition of two pre-bleached images with a delay of 1 minute between them. After photobleaching, images were taken every minute for a total of 20 min. Each image was taken with 100 ms camera exposure with 10% 568 nm laser intensity and consisted of a z-stack of 33 slices captured every 0.5 µm. The acquisition was performed on three different days for each cell line analysed.

### FRAP analysis

Maximum intensity z-projections were acquired and the fluorescence loss due to photobleaching across the timecourse was corrected for using the bleach correction plugin in ImageJ/FiJi, fitting to an exponential decay. The fluorescence intensity measured within the bleached region was normalised to the background intensity, measured outside of the cell, and the fluorescence recovery was then represented as a percentage relative to the fluorescence intensity pre-bleaching. Curves showing the averaged recovery of all cells analysed for each condition as a function of time were plotted using Igor Pro 9. The curves were then fitted with a single exponential function in Igor Pro 9 in order to determine the immobile population and residency time.

3 $$f\left(t\right)=\,{y}_{0}+A{e}^{\frac{-(t-{t}_{0})}{\tau }}$$

### Immunofluorescence

Cells were plated on gelatinised 35 mm µ-dishes two days before fixation. After two washes with PBS, cells were fixed in 2% paraformaldehyde for 15 min at RT and permeabilized in 0.4% Triton X−100 for 5 min at RT. Cells were briefly washed with PBS, blocked for three periods of 2 min with 0.2% fish gelatine in PBS and incubated with primary antibody (anti-SMCHD1 1:1000 (made in-house), anti-H3K27me3 1:1000 (Active Motif, # 61017) diluted in fish gelatine solution with 5% normal goat serum for 2 h at RT in a humid chamber. Samples were washed three times with fish gelatine solution and incubated for 1 h at RT with secondary antibody (Alexa Fluor 568 goat anti-mouse IgG, Alexa Fluor 488 goat anti-rabbit IgG) 1:500 diluted in fish gelatine solution. Following two washes with fish gelatine solution and one wash with PBS, Vectashield with DAPI was added, and the µ-dish lid was locked with parafilm. Cells were analysed on the Olympus IX83 microscope.

### H3K27me3 domain size analysis

Z-stack images taken with the Olympus IX83 microscope were analysed using the 3D Objects Counter function in Fiji/ImageJ. For each image, a threshold was set in order to highlight the H3K27me3 focus in each cell and the volume of each focus was chosen as an output. The object map was cross-checked against the original image to make sure the foci have been correctly identified. For each cell line, the volume values were plotted as a violin plot using Igor Pro 9.

### Chromatin-associated RNA extraction and sequencing (ChrRNA-seq)

mESCs growing without feeders or ML-differentiated cells were pelleted from confluent 90 mm dishes, washed once with PBS and snap-frozen for storage at -80 °C. The pellets were resuspended in HLBN buffer (0.01 M NaCl, 2.5 mM MgCl_2_, 10 mM Tris pH 7.5, 0.1% NP-40). Then the cells were underlaid with HLBN buffer supplemented with 24% sucrose, and nuclei were isolated through centrifugation (1000 g 5 min at 4 °C). Nuclei were resuspended in NUN1 (75 mM NaCl, 20 mM Tris pH 7.9, 0.5 mM EDTA, 50% glycerol, 0.1 mM DTT) and then lysed with NUN2 (0.3 M NaCl, 20 mM HEPES pH 7.6, 7.5 mM MgCl_2_, 0.2 mM EDTA, 1 M urea, 1% NP-40, 0.1 mM DTT) by incubating 15 min on ice and occasionally vortexing. The chromatin was isolated by centrifugation (15000 rpm 10 min at 4 °C) and resuspended in HSB buffer (0.5 M NaCl, 10 mM Tris pH 7.5, 10 mM MgCl_2_). The samples were DNAse (LifeTech) treated at 37 °C, shaking (1400 rpm) until the chromatin pellet dissolved. Samples were then treated with 0.2% SDS and 0.4 mg/ml proteinase K for 30 min at 37 °C, shaking (1400 rpm). To obtain the chromatin-associated RNA, two rounds of standard TRIzol- (Invitrogen) chloroform extraction with overnight isopropanol precipitation were performed. Between the two rounds, a second DNAse treatment was performed for 30 min at 37 °C, shaking (1400 rpm). After the second round, the RNA pellets were resuspended in RNase-free water and their concentration was measured by NanoDrop (ThermoFisher). 100-500 ng of RNA was used for library preparation with the Illumina TruSeq stranded total RNA kit (RS−122-2301). The pooled libraries were sequenced by 2 × 81 paired-end sequencing using Illumina NextSeq500 (FC-404-2002).

### ChrRNA-seq analysis

The analysis of the ChrRNA-seq datasets was done as previously described^38^. Briefly, raw fastq files were mapped using bowtie2^58^ (v2.3.2) to rRNA, and the rRNA-mapped reads were discarded. Subsequently, the unmapped reads were aligned to an N-masked mm10 genome with STAR^59^ (v2.4.2a or v2.7.9a) with parameters “*--twopassMode Basic --outSAMstrandField intronMotif --outSAMattributes All --outFilterMultimapNmax 1 --outFilterMismatchNoverReadLmax 0.06 --alignEndsType EndToEnd*”. Unique alignments were then split into the two alleles, Cast and 129S, using SNPsplit^60^ (v0.4.0dev). featureCounts programme^61^, with parameters “*-t transcript -g gene_id -s 2*”) was used to count the allelic and unsplit reads that overlap with genes. Samtools^62^ (v1.16.1) was used to filter and sort the alignments. Only genes with at least 10 SNP-covering reads across samples for comparisons were used to calculate the allelic ratio Xi/(Xi+Xa) (Xi represents the inactive allele which here is the 129 one, while Xa represents the active allele which here is the Cast one). The genes were split into three categories according to their dependency on SMCHD1 for silencing as previously defined in ref. ^13^. Boxplots were generated using R package ggplot2 and p-values were calculated using Paired *t* test. The designed mutations (i.e., G137E, E147A, Y353C, K518E) for SMCHD1 are confirmed by the ChrRNA-seq reads and are visualised using IGV.

### Whole cell lysates, RIPA extracts, nuclear extracts and western blot

Whole cell lysates were obtained from mESCs growing without feeders, by washing the cells with PBS and then lysing them with SMASH buffer (0.125 M Tris pH 6.8, 11.6% glycerol, 4% SDS, 0.002% bromophenol blue, 10% β-mercaptoethanol). The lysates were snap-frozen and stored at -80 °C until use.

For nuclear extracts, mESCs growing without feeders were pelleted, washed with PBS, snap-frozen on dry ice and stored at -80 °C. The pellets were resuspended in hypotonic buffer A (10 mM HEPES pH 7.9, 1.5 mM MgCl_2_, 10 mM KCl) supplemented with 0.5 mM DTT, 0.5 mM PMSF and 1x complete protease inhibitors (Roche) and incubated on ice for 10 min. Cells recovered by centrifugation were lysed in buffer A with 0.1% NP-40 supplemented with 5 mM DTT, 0.5 mM PMSF and 1x complete protease inhibitors and incubated on ice for 10 min. The nuclei were then isolated by centrifugation, washed once with PBS supplemented with 1x complete protease inhibitors, and resuspended in high salt buffer C (5 mM HEPES pH 7.9, 26% glycerol, 1.5 mM MgCl_2_, 0.2 mM EDTA, 350 mM NaCl) supplemented with 0.5 mM DTT and 1x complete protease inhibitors. The extraction was done on ice for 1 h, and the nuclei were then pelleted with the supernatant kept as the nuclear extract, which was snap-frozen and stored at -80°C until use. The concentration of the extract was determined using the Bio-Rad Bradford assay according to the manufacturer’s protocol. To analyse the chromatin insoluble fraction, the last nuclear pellet obtained was resuspended in SMASH buffer, snap-frozen and stored at -80°C until use.

The extracts obtained through one of the methods above were separated on a home-made SDS-PAGE separating gel with a stacking gel or a commercial NuPAGE gel (ThermoFisher). The proteins were transferred onto a nitrocellulose membrane (Bio-Rad) using the Bio-Rad Trans-Blot Turbo Transfer system, with High MW or Mixed MW preprogrammed protocols depending on the protein size. Membranes were blocked for 1 h at RT in PBST (PBS with 0.1% Tween 20) with 5% milk and incubated with primary antibodies (summarised in Table S5) diluted in blocking solution, overnight shaking at 4 °C. Following washes with PBST, membranes were incubated for 1 h at RT with secondary antibodies conjugated to horseradish peroxidase (Amersham) diluted 1:2000 in blocking solution. Membranes were washed with PBST, and the bands were revealed using enhanced chemiluminescence (ECL) solution (ThermoFisher) and developed on chemiluminescent sensitive photographic film (Amersham). Uncropped scans are provided as Source Data.

### AlphaFold modelling

SMCHD1 structure predictions were performed using AlphaFold3^63^ via the AlphaFold server. The input sequence was two copies of full-length mouse SMCHD1 (WT or mutant sequence) with two ATP molecules and two Mg2+ ions. Five models were generated for each mutant, and these all agreed on the structure of the head domains. The modelled structures were visualised using PyMol molecular graphics system v 3.0.3. PAE plots were generated using PAEViewer^64^.

### Satistics & reproducibility

No statistical methods were used to predetermine sample sizes. Samples sizes are similar to those used in previous publications. No data were excluded for the analysis. The number of biological replicates are indicated in the figure legend or method section. Statistical tests were performed with R (v 4.2.1), Igor Pro 9 (WaveMetrics). For tissue culture based experiments, all wells in each biological replicate were split from the same batch of cells and randomly divided for each type of experiment. All the imaging experiments were performed and analysed by a single person and therefore were not blinded. For chRNA-seq, analysis was all performed by software programmes/algorithms.

### Reporting summary

Further information on research design is available in the Nature Portfolio Reporting Summary linked to this article.

## Supplementary information

Supplementary Information

Reporting Summary

Transparent Peer Review file

## Source data

Source Data

## Data Availability

High-throughput raw sequencing data (ChrRNA-seq) as well as key processed data generated in this study are deposited to the National Centre for Biotechnology Information’s Gene Expression Omnibus with accession number GSE295772 (https://www.ncbi.nlm.nih.gov/geo/query/acc.cgi?acc=GSE295772). Source data are provided with this paper.

## Code availability

Codes used for the Xi-specific SPT analysis are available via GitHub at (https://github.com/aleks-szczure/Constantinescu-et-al.-2026-NATCOM/).
