# Supplementary material for: Selective interaction of SMCHD1 with chromatin is governed by LRIF1 and SMCHD1 ATPase activity
Source: Nat Commun. 2026 Jun 16;17:7636. doi: 10.1038/s41467-026-74427-9 (PMC13434421; doi:10.1038/s41467-026-74427-9)
Supplement: Supplementary file 1 [file 41467_2026_74427_MOESM1_ESM.pdf]

Selective interaction of SMCHD1 with chromatin is governed by LRIF1 and SMCHD1 ATPase activity

Supplementary Figures

Authors;

Flavia Constantinescu[1], Aleksander T Szczurek[2], Tatyana B Nesterova[1], Guifeng Wei[1], Mafalda Almeida[3], Jessica R. Kelley[4], Victoria Pustygina[1], Stephan Uphoff[5], Adam D Cawte[1] and Neil Brockdorff[1,*]

Affiliations.

1. Developmental Epigenetics, Dept of Biochemistry University of Oxford, South Parks Road, Oxford, OX1 3QU, UK

2. Epigenetic regulation of chromatin function, Dept of Biochemistry University of Oxford, South Parks Road, Oxford, OX1 3QU, UK

3. Transposon lab, Dept of Biochemistry University of Oxford, South Parks Road, Oxford, OX1 3QU, UK

4. Chromatin Dynamics, Dept of Physics, Parks Road, Oxford, OX1 3PU, UK

5. Bacterial DNA repair and mutagenesis, Dept of Biochemistry University of Oxford, South Parks Road, Oxford, OX1 3QU, UK

* Correspondence; neil.brockdorff@bioch.ox.ac.uk

Supplementary Figure 1

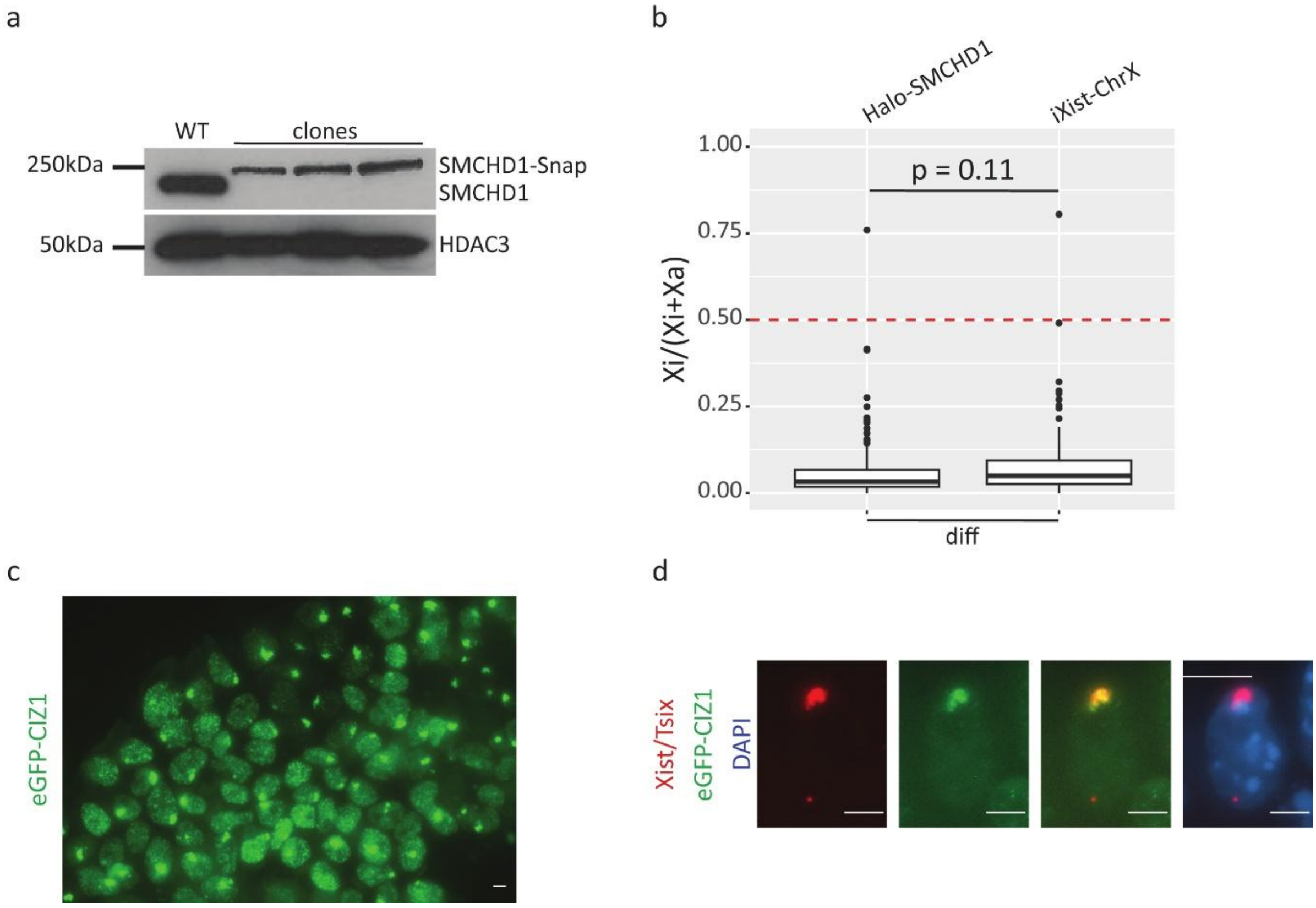

Supplementary Fig. 1 – Validation of a model system used to study the dynamic behaviour of SMCHD1

a. Western Blot showing the level of expression of SMCHD1-Snap in three independent clones. b. Box plot showing allelic ChrRNA-seq analysis of X-linked gene (n=184) silencing in Halo-SMCHD1 clone G2 and iXist-ChrX mESCs. Boxes are averaged from two (for iXist-ChrX) or three (for Halo-SMCHD1) replicate differentiation experiments. Statistical analysis - two-sided paired t-test. Boxplots: centre lines indicate the median, box limits indicate the first and third quartiles and whiskers indicate 1.5× the interquartile range (IQR). c. Representative image of the proportion of Halo-SMCHD1 mESCs showing CIZ1-marked Xi domain following 24h doxycycline treatment. Image is a maximum-intensity projection, scale bar - 5μm. d. Representative images of Xist FISH and eGFP-CIZ1 signal in fixed samples of Halo-SMCHD1 mESC line following 24h doxycycline addition. Images are maximum-intensity projections, scale bar - 5μm. Source data are provided as a Source Data file.

Supplementary Figure 2

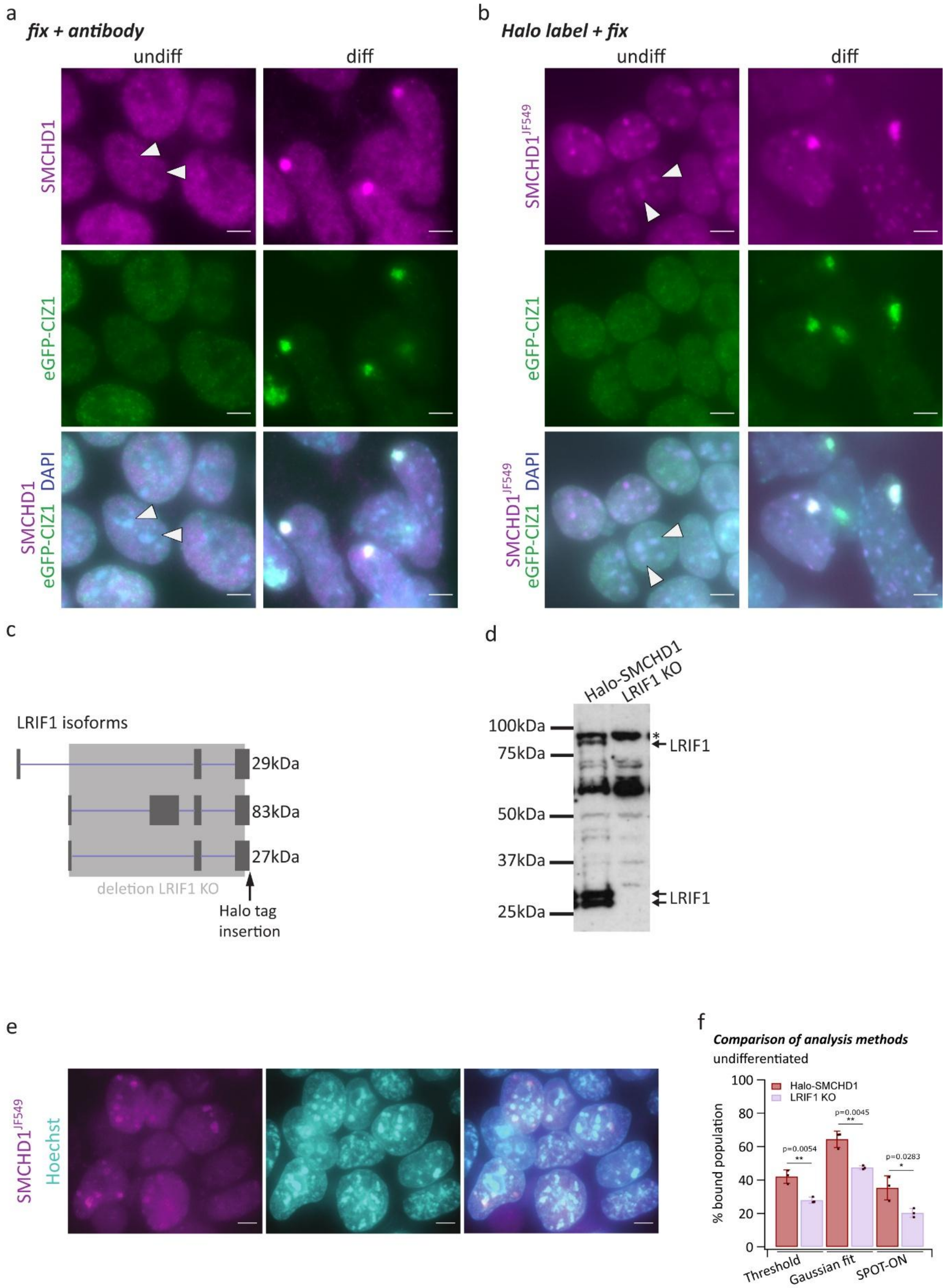

Supplementary Fig. 2 – Characteristics of SMCHD1 enrichment over PCH domains

a. Representative images of SMCHD1 immunostaining in fixed samples from Halo-SMCHD1 mESCs undifferentiated or differentiated for 7 days. Arrowheads point towards examples of PCH domains. b. Representative images of Halo labelling prior to sample fixation from Halo-SMCHD1 mESCs undifferentiated or differentiated for 7 days. Arrowheads point towards examples of PCH domains. c. Schematic showing the three *LRIF1* isoforms and the engineering strategies for KO and C-terminal tagging. d. Western Blot showing the expression of the three LRIF1 isoforms in Halo-SMCHD1 and *Lrif1*$^{-/-}$ mESCs. * indicates non-specific band. e. Representative live-cell image of Lrif1$^{-/-}$ mESCs showing retention of SMCHD1 at some PCH regions. All images are maximum-intensity projections, scale bar - 5µm. f. Bar plots showing the average percentage of bound molecules of SMCHD1 the indicated mESC lines (3 independent replicates each), using three different analysis methods. Error bars represent the STDEV (two-sided unpaired t-test). Source data are provided as a Source Data file.

Supplementary Figure 3

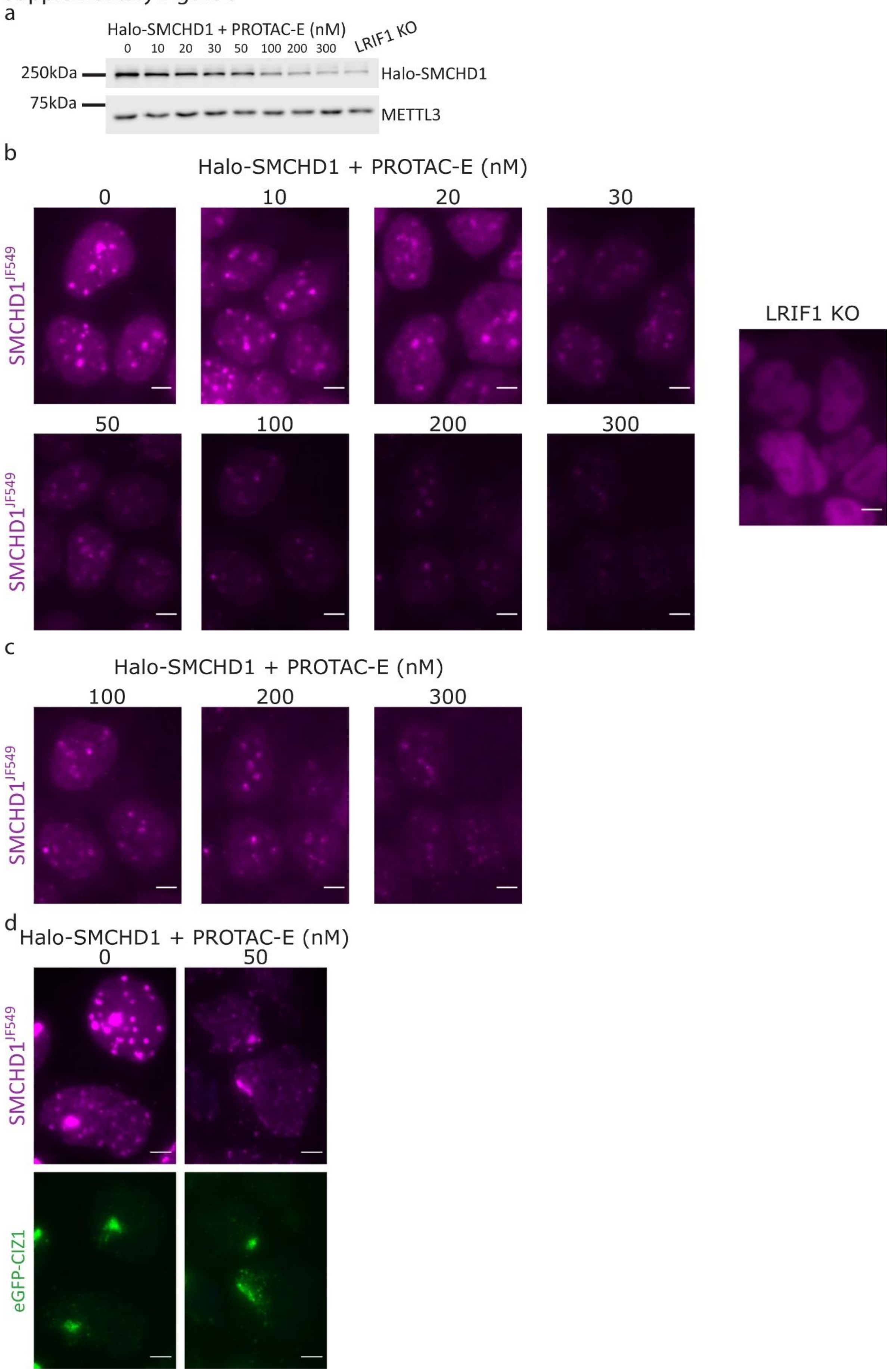

Supplementary Fig. 3 – SMCHD1 Localization patterns after reducing protein levels using Halo-PROTAC-E

a. Western Blot showing reduction in SMCHD1 protein levels after treatment with increasing concentrations of Halo-PROTAC-E. b. Representative live-cell images of Halo-SMCHD1 mESC line treated with increasing concentrations of Halo-PROTAC-E ligand prior to labelling with JF549. Image of $Lrif^{-/-}$ mESC line is shown for comparison. c. Images from b. shown with enhanced contrast to allow for better visualisation of the distribution pattern at lower level of the protein. d. Representative live-cell images of Halo-SMCHD1 mESC line treated with 50nM Halo-PROTAC-E ligand prior to labelling with JF549 and differentiation for 7 days to identify Xi localised SMCHD1. All images are maximum-intensity projections. Scale bars correspond to 5μm.

Supplementary Figure 4

a

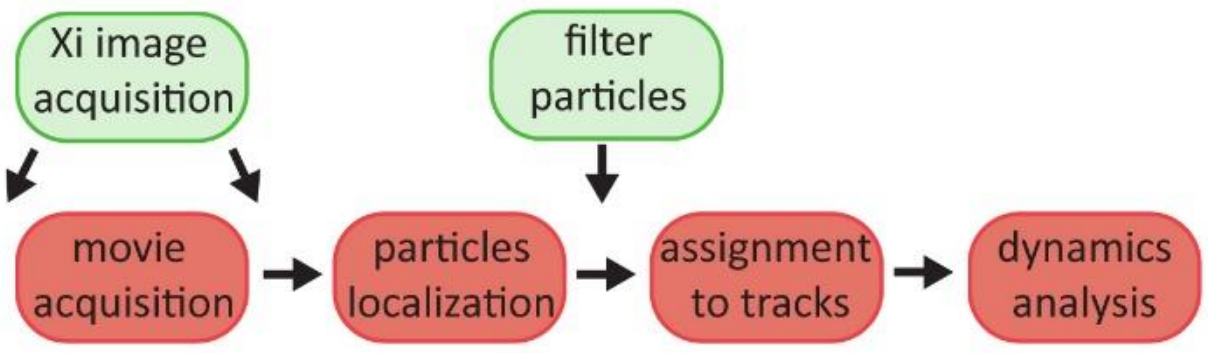

b

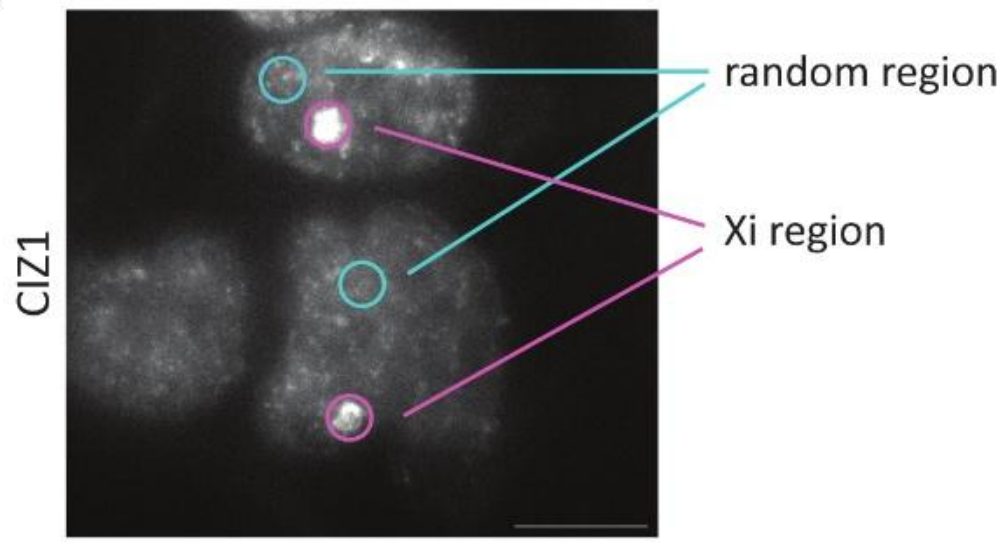

c

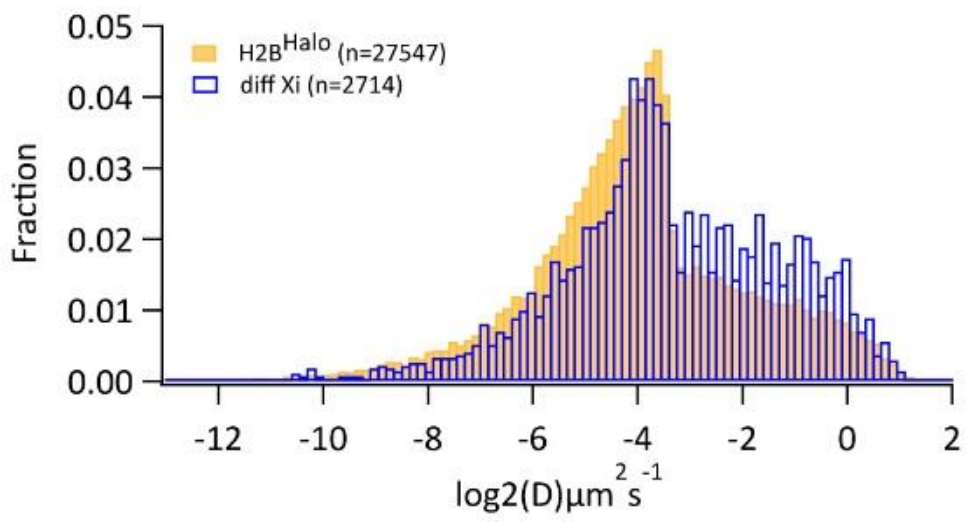

d

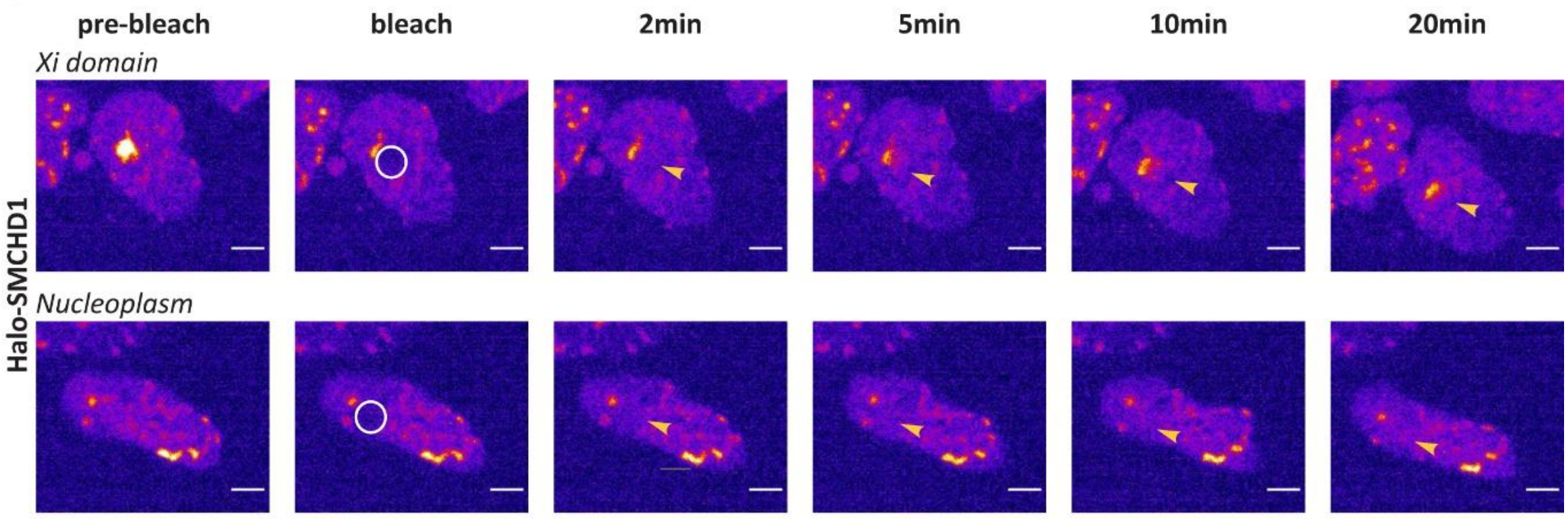

e

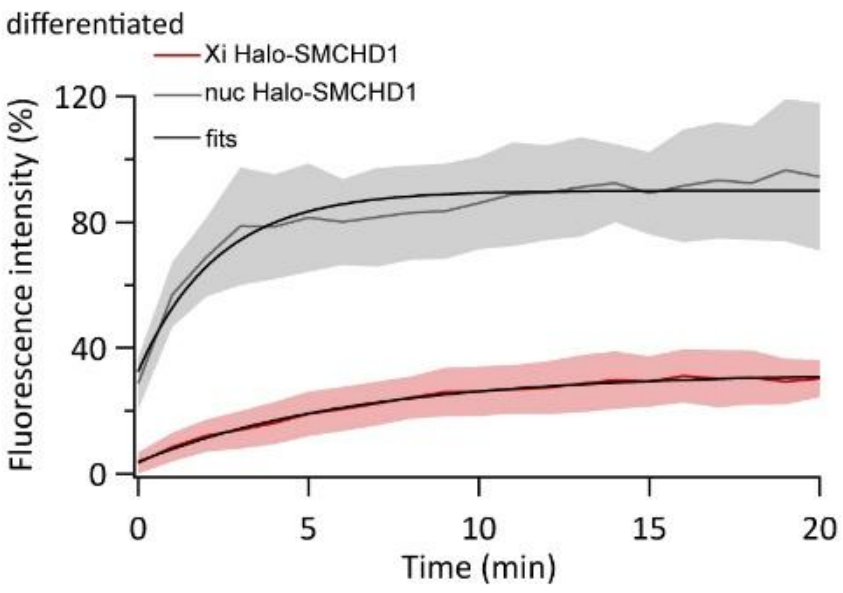

f

	Halo-SMCHD1 Xi domain	Halo-SMCHD1 nucleoplasm
Immobile population	68%	10%
Residency	6.4 min	2.3 min

Supplementary Fig. 4 – Characterisation of SMCHD1 Xi-specific dynamic behaviour

a. Schematic of Xi-focused SPT analysis. b. Representative image of Xi domains marked by CIZ1 accumulation, taken on the TIRF/HILO microscope used for SPT. Two examples of areas used for control random regions are also shown. Scale bar - 10μm. c. In blue, same as in Fig. 3b. In yellow, normalized histogram of diffusion coefficients of tracks from across the whole nucleus in histone H2B-Halo mESCs. d. Representative images from a FRAP experiment using Halo-SMCHD1 mESC differentiated for 7 days. The bleached region is marked by a white circle on the bleach snapshot and by a yellow arrow in the post-bleach snapshots. Scale bar - 5μm. e. Single exponential fits of the average fluorescence recovery curves over the Xi domain or a random nucleoplasmic region in Halo-SMCHD1 mESC. f. Summary of the immobile population and residency values extracted from the fits shown in e. Source data are provided as a Source Data file.

Supplementary Figure 5

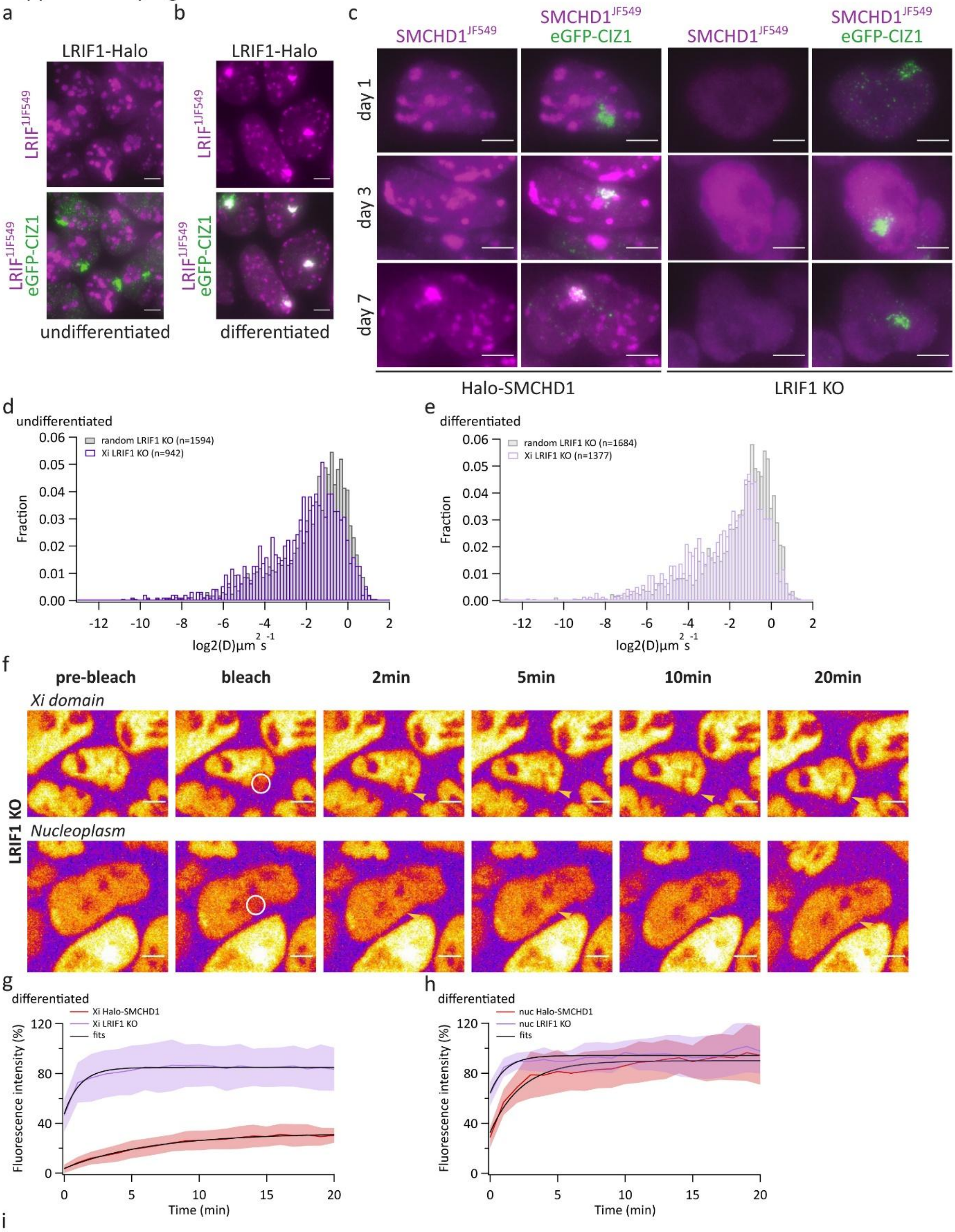

i

	Halo-SMCHD1 Xi domain	Halo-SMCHD1 nucleoplasm	LRIF1 KO Xi domain	LRIF1 KO nucleoplasm
Immobile population	68%	10%	15%	6%
Residency	6.4 min	2.3 min	1.2 min	1.1 min

Supplementary Fig. 5 – Absence of LRIF1 leads to loss of the SMCHD1 Xi-specific dynamic behaviour

a-b. Representative live-cell images of LRIF1-Halo mESC line, taken after 24h doxycycline addition (a) or 7 days of differentiation (b). c. Representative live images showing gradual enrichment or no enrichment of SMCHD1 over the Xi domain marked by eGFP-CIZ1 during differentiation in Halo-SMCHD1 and Lrif1$^{-/-}$ mESCs, respectively. All images are maximum-intensity projections, scale bar - 5μm. d. Normalized histogram of diffusion coefficients of tracks from the Xi (dark purple) or a random region (dark grey) in undifferentiated Lrif1$^{-/-}$ mESCs, kept for 24h with doxycycline e. Normalized histogram of diffusion coefficients of tracks from the Xi (light purple) or a random region (light grey) in Lrif1$^{-/-}$ mESCs, differentiated for 7 days.f. Representative images from a FRAP experiment using the Lrif1$^{-/-}$ mESCs differentiated for 7 days. The bleached region is marked by a white circle on the bleach snapshot and by a yellow arrow in the post-bleach snapshots. Scale bar - 5μm. g-h. Single exponential fits of the average fluorescence recovery curves over the Xi domain (g) or a random nucleoplasmic region (h). i. Summary of the immobile population and residency values extracted from the fits shown in g-h. Source data are provided as a Source Data file.

Supplementary Figure 6

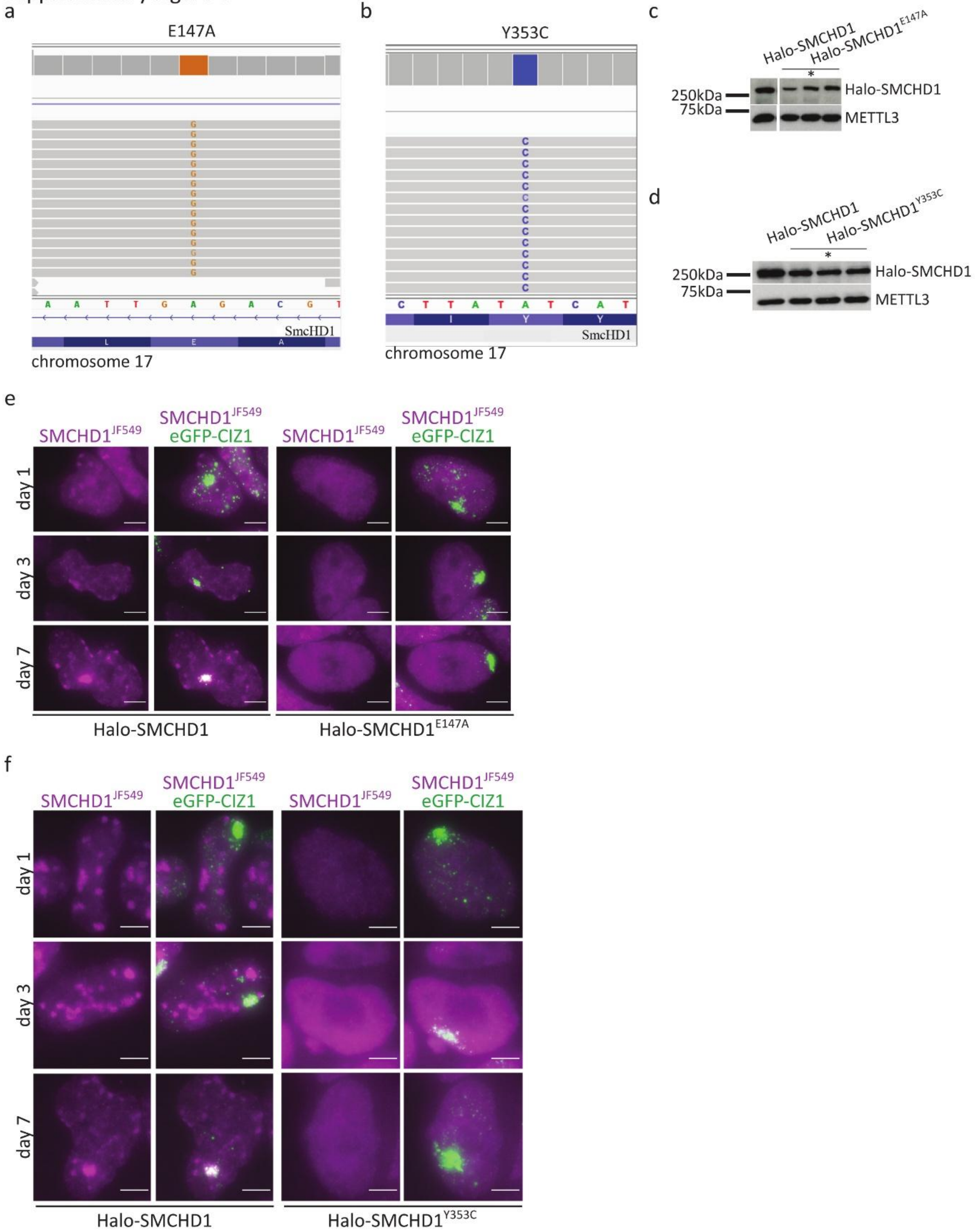

Supplementary Fig. 6 – SMCHD1 GHKL ATPase loss-of-function mutations alter the protein's stability and accumulation over the Xi

a-b. Genome browser screenshot of chrRNA-seq reads showing homozygosity for the selected Halo-SMCHD1^{E147A} (a) or Halo-SMCHD1^{Y353C} (b) clones. c-d. Western Blot showing the expression of SMCHD1 in independent Halo-SMCHD1^{E147A} (c) or Halo-SMCHD1^{Y353C} (d) clones. * marks the clones which were selected for further experiments for each cell line. In c, the extracts were run on the same gel but in non-adjacent lanes. e-f. Representative live images showing there is gradual enrichment or no enrichment of SMCHD1 over the Xi domain marked by eGFP-CIZ1 during differentiation in Halo-SMCHD1, Halo-SMCHD1^{E147A} or Halo-SMCHD1^{Y353C} mESCs. Images are maximum-intensity projections, scale bar - 5μm. Source data are provided as a Source Data file.

Supplementary Figure 7

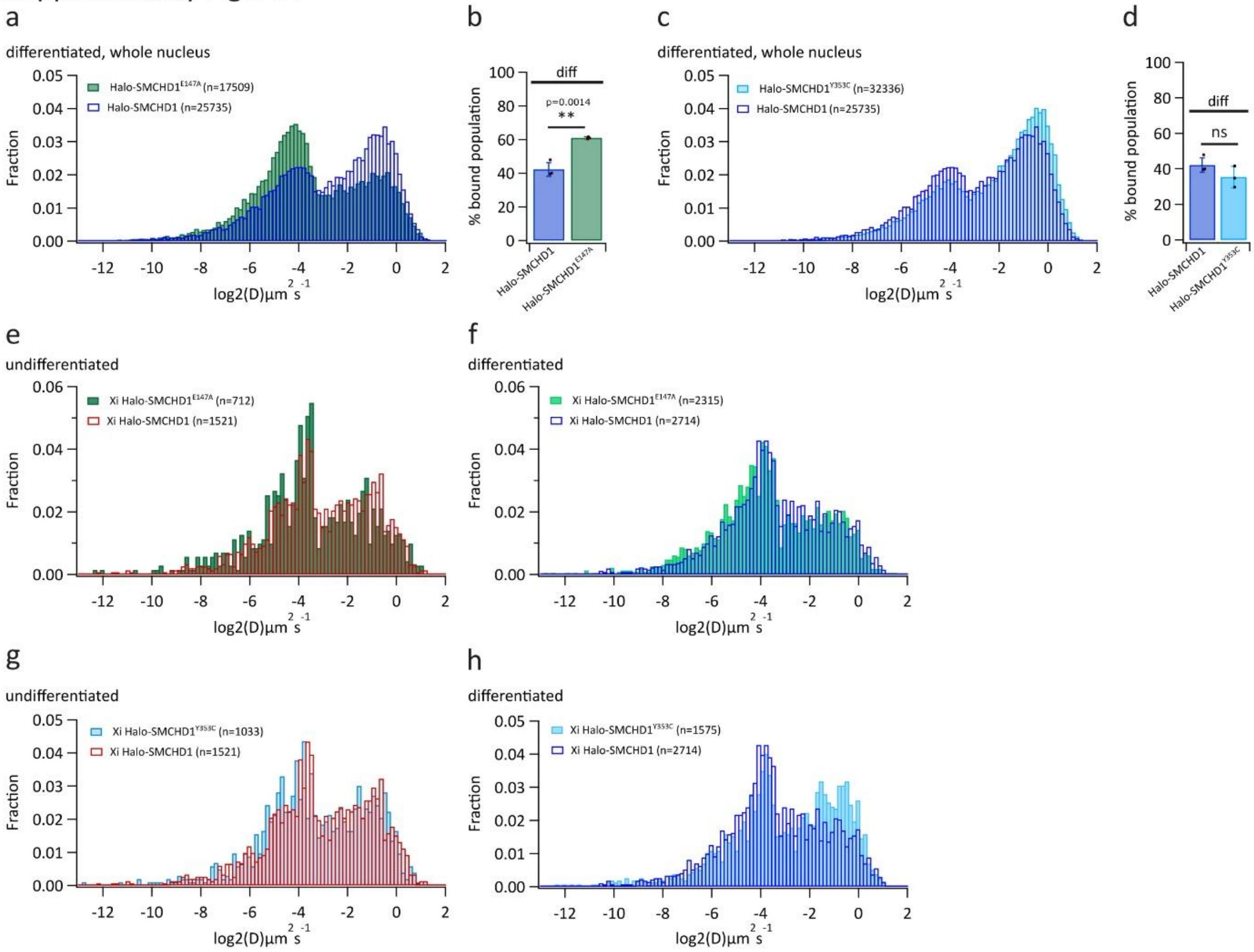

Supplementary Fig. 7 – Characterization of the dynamic behaviour of SMCHD1 GHKL ATPase loss-of-function mutants using SPT

a. Normalized histogram of diffusion coefficients of tracks from across the whole nucleus in indicated cell lines differentiated for 7 days. b. Bar plots showing the average percentage of bound molecules of SMCHD1 (3 independent replicates each). Error bars represent the STDEV (two-sided unpaired t-test). c. In blue, same as in a. In turquoise, normalized histogram of diffusion coefficients of tracks from across the whole nucleus in Halo-SMCHD1^{Y353C} mESCs differentiated for 7 days. d. Bar plots showing the average percentage of bound molecules of SMCHD1 (3 independent replicates each). Error bars represent the STDEV (two-sided unpaired t-test). e, g. Normalized histogram of diffusion coefficients of tracks from the Xi in indicated cell lines, undifferentiated, treated for 24h with doxycycline. f, h. Normalized histogram of diffusion coefficients of tracks from the Xi in indicated cell lines, differentiated for 7 days. diff = differentiated. Source data are provided as a Source Data file.

Supplementary Figure 8

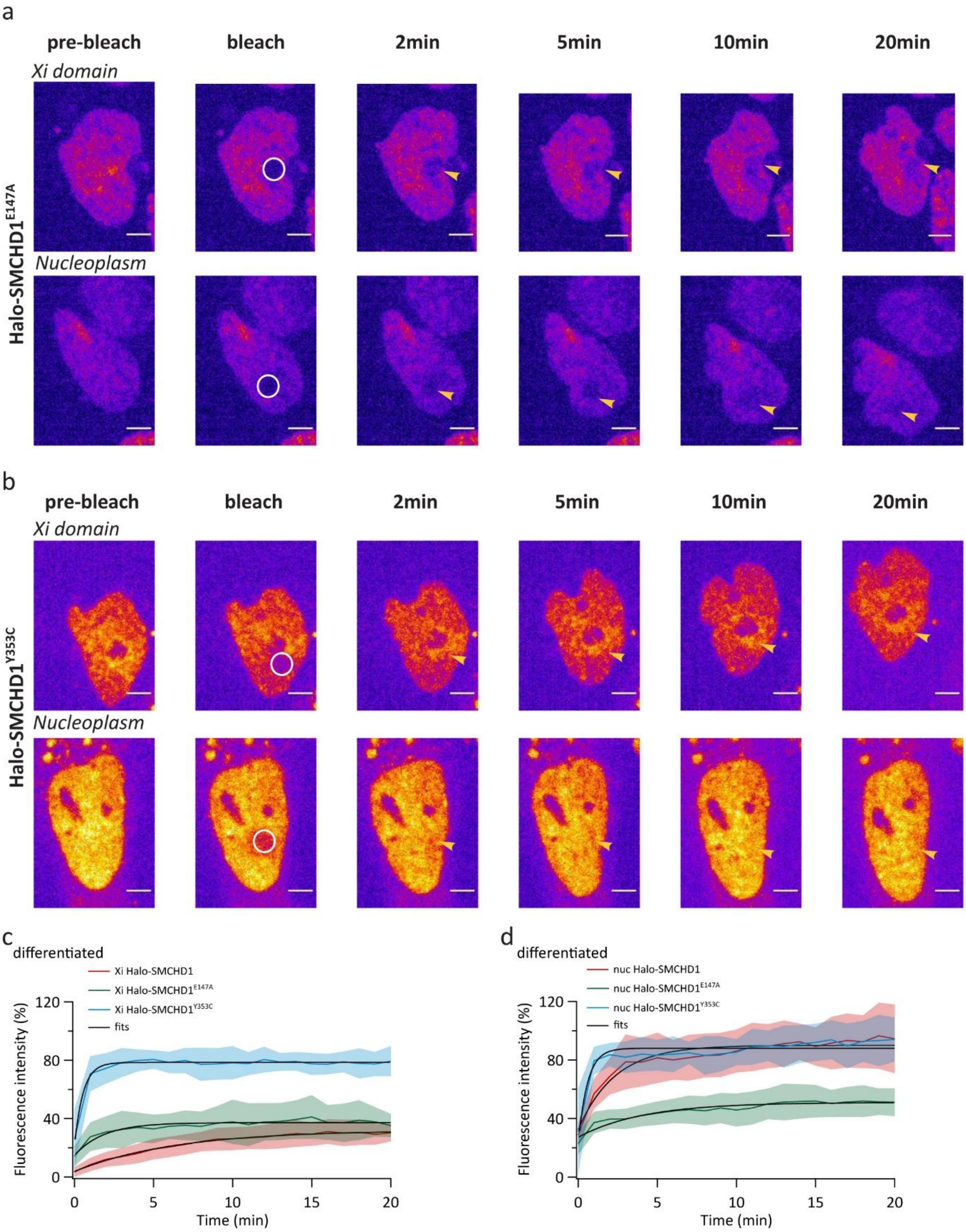

e

	Halo-SMCHD1 Xi domain	Halo-SMCHD1 nucleoplasm	E147A Xi domain	E147A nucleoplasm	Y353C Xi domain	Y353C nucleoplasm
Immobile population	68%	10%	63%	49%	22%	12%
Residency	6.4 min	2.3 min	1.9 min	4.1 min	0.6 min	0.6 min

Supplementary Fig. 8 - FRAP analysis of the dynamic behaviour of SMCHD1 GHKL ATPase loss-of-function mutants

a-b. Representative images from a FRAP experiment using Halo-SMCHD1^{E147A} (a) or Halo-SMCHD1^{Y353C} (b) mESCs differentiated for 7 days. The bleached region is marked by a white circle on the bleach snapshot and by a yellow arrow in the post-bleach snapshots. Scale bar - 5µm. c-d. Single exponential fits of the average fluorescence recovery curves over the Xi (c) or a random nucleoplasmic region (d). e. Summary of the immobile population and residency values extracted from the fits shown in c-d. Source data are provided as a Source Data file.

Supplementary Figure 9

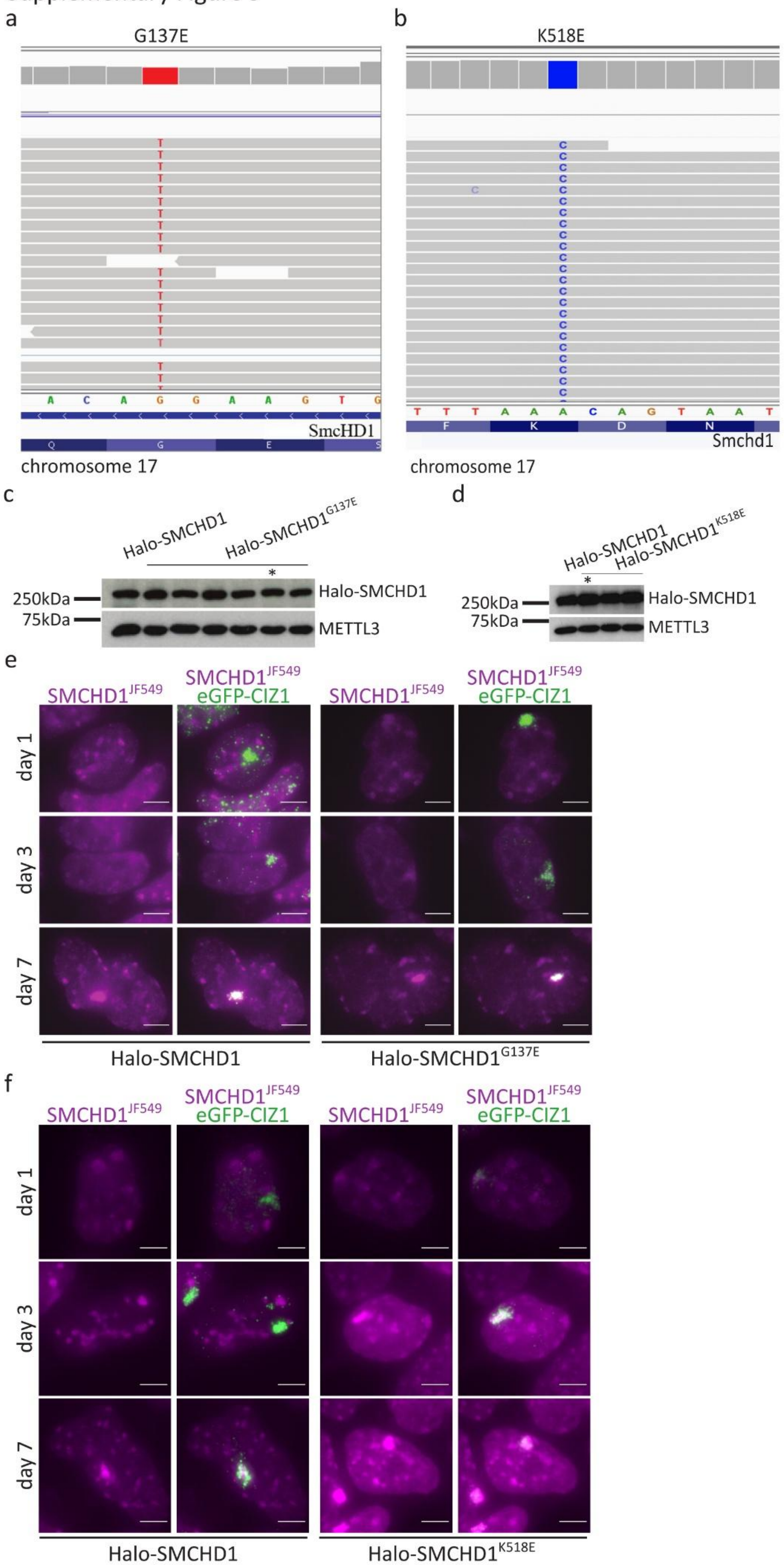

Supplementary Fig. 9 – SMCHD1 GHKL ATPase gain-of-function mutations don't impair the protein's stability and accumulation over the Xi

a-b. Genome browser screenshot of chrRNA-seq reads showing homozygosity for the selected Halo-SMCHD1^{G137E} (a) or Halo-SMCHD1^{K518E} (b) clone. c-d. Western Blot showing the expression of SMCHD1 in Halo-SMCHD1^{G137E} (c) or Halo-SMCHD1^{K518E} (d) clones. * marks the clone selected for further experiments. e-f. Representative live-cell images showing gradual enrichment of SMCHD1 over the Xi marked by eGFP-CIZ1 during differentiation in Halo-SMCHD1 and Halo-SMCHD1^{G137E} (e) or Halo-SMCHD1^{K518E} (f) mESCs. Images are maximum-intensity projections, scale bar - 5μm. Source data are provided as a Source Data file.

Supplementary Figure 10

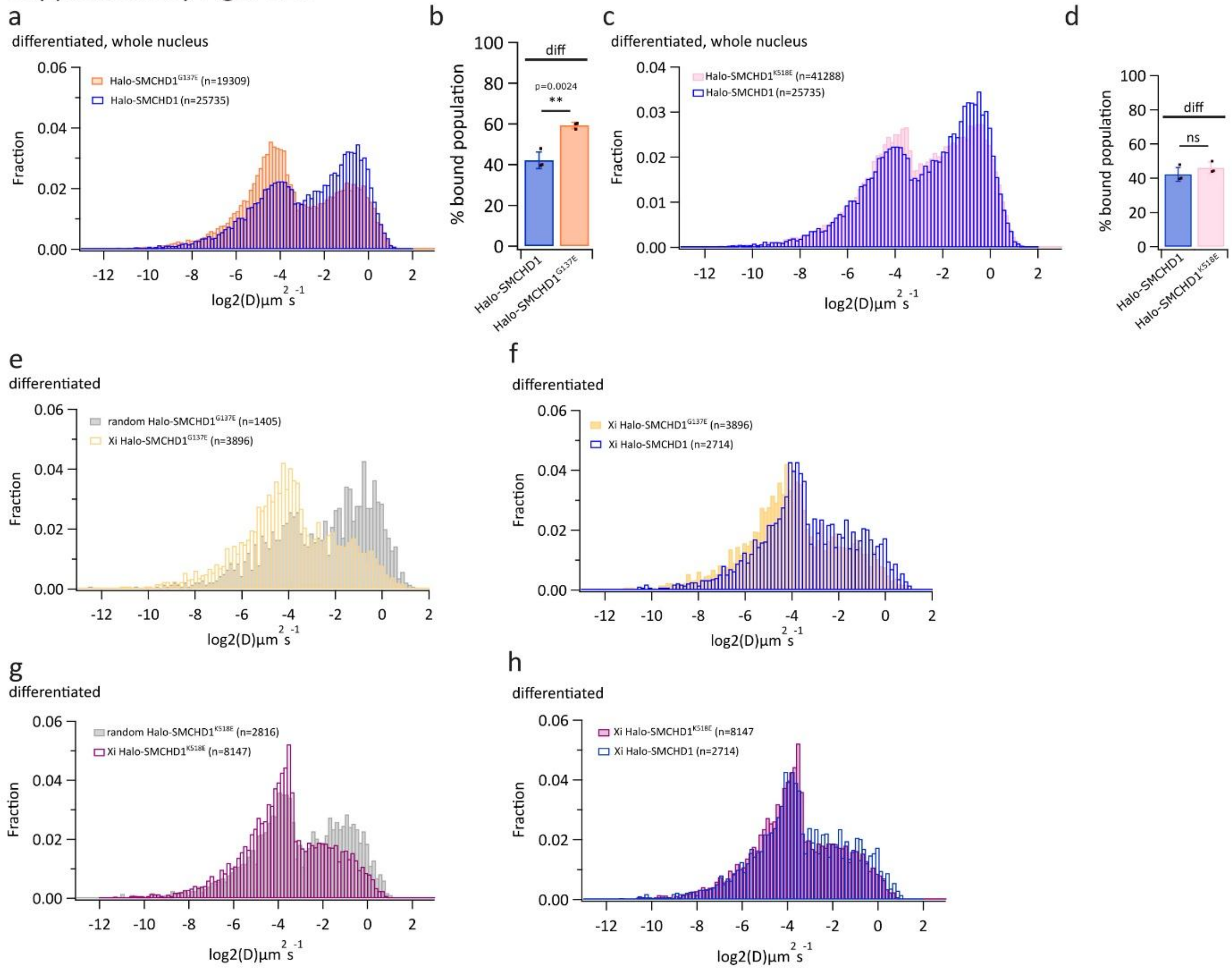

Supplementary Fig. 10 - Characterization of effects of GHKL ATPase gain-of-function mutations of SMCHD1 on its SPT-determined dynamic behaviour

a, c. Normalized histogram of diffusion coefficients of tracks from across the whole nucleus in indicated cell lines, differentiated for 7 days.e. b,d. Bar plots showing the average percentage of bound molecules of SMCHD1 (3 independent replicates each). Error bars represent the STDEV (two-sided unpaired t-test). e. Normalized histogram of diffusion coefficients of tracks from across the Xi (yellow) or a random region (light grey) in Halo-SMCHD1^{G137E} mESCs differentiated for 7 days. f. Normalized histogram of diffusion coefficients of tracks from the Xi in indicated cell lines, differentiated for 7 days. g. Normalized histogram of diffusion coefficients of tracks from across the Xi (purple) or a random region (light grey) in Halo-SMCHD1^{K518E} mESCs differentiated for 7 days. h. Normalized histogram of diffusion coefficients of tracks from the Xi in indicated cell lines, differentiated for 7 days. Source data are provided as a Source Data file.

Supplementary Figure 11

a

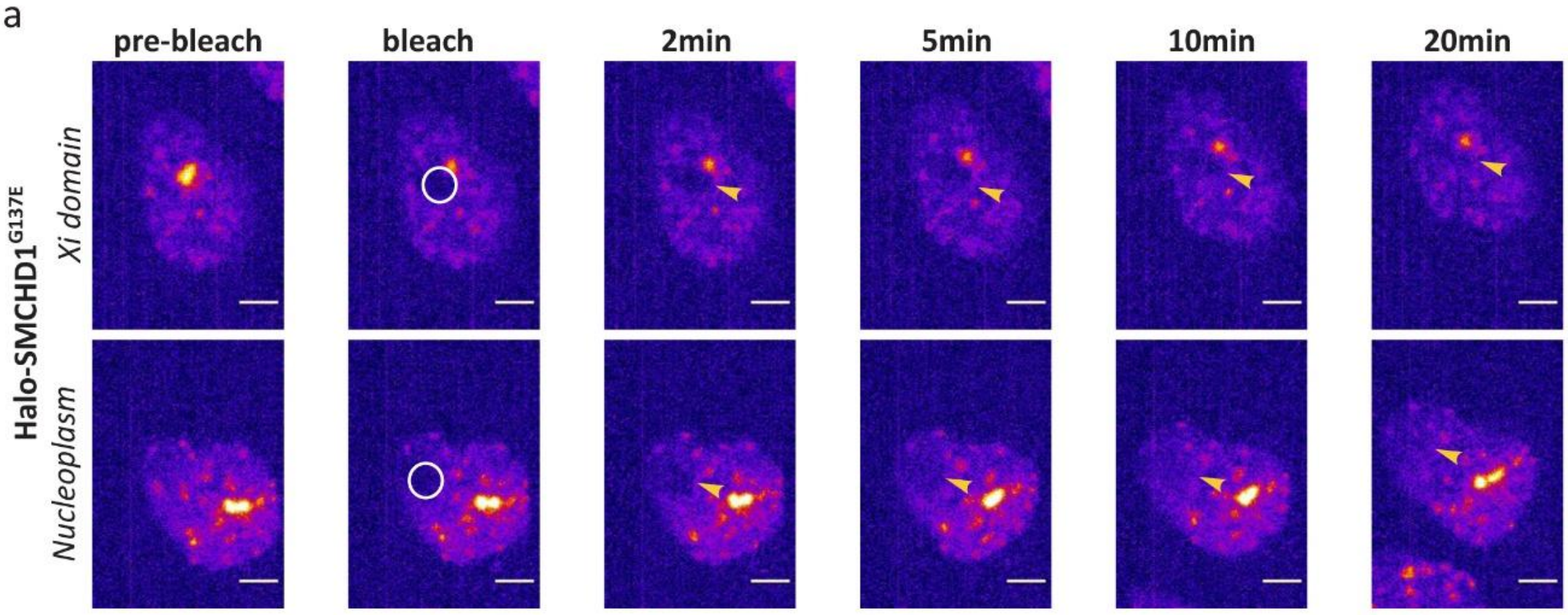

b

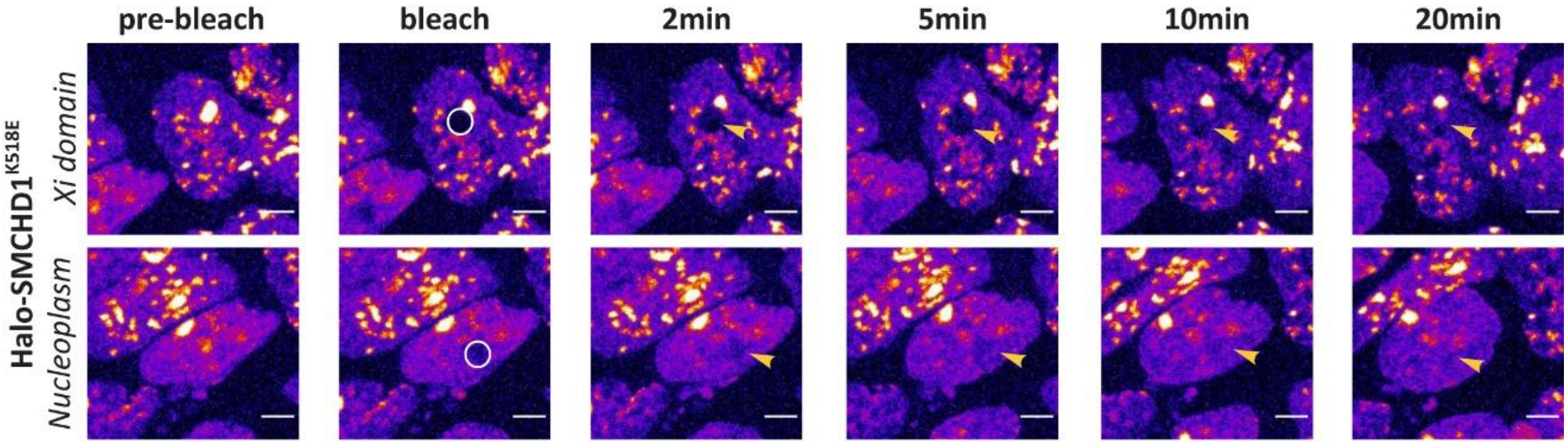

c

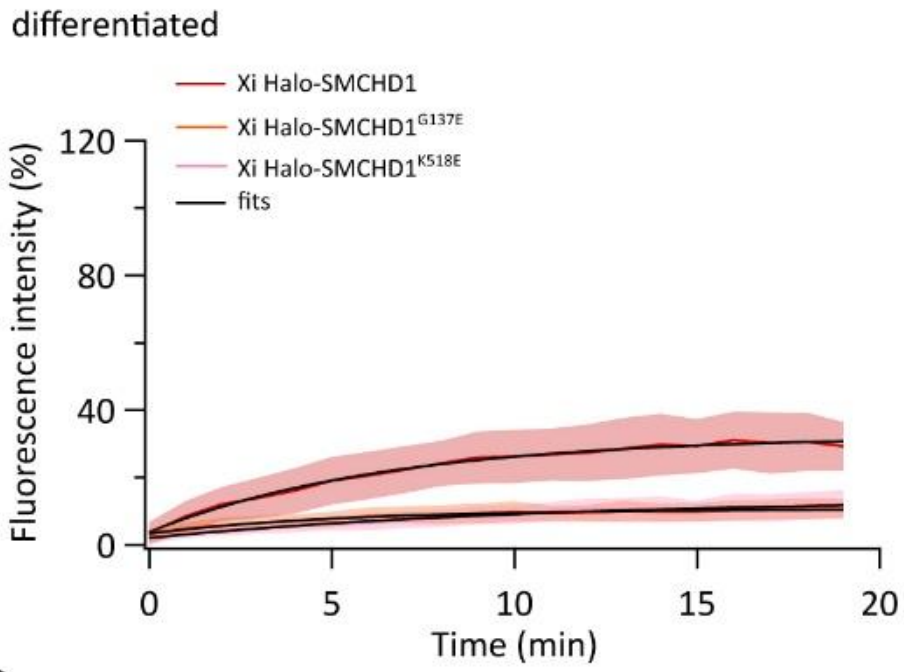

d

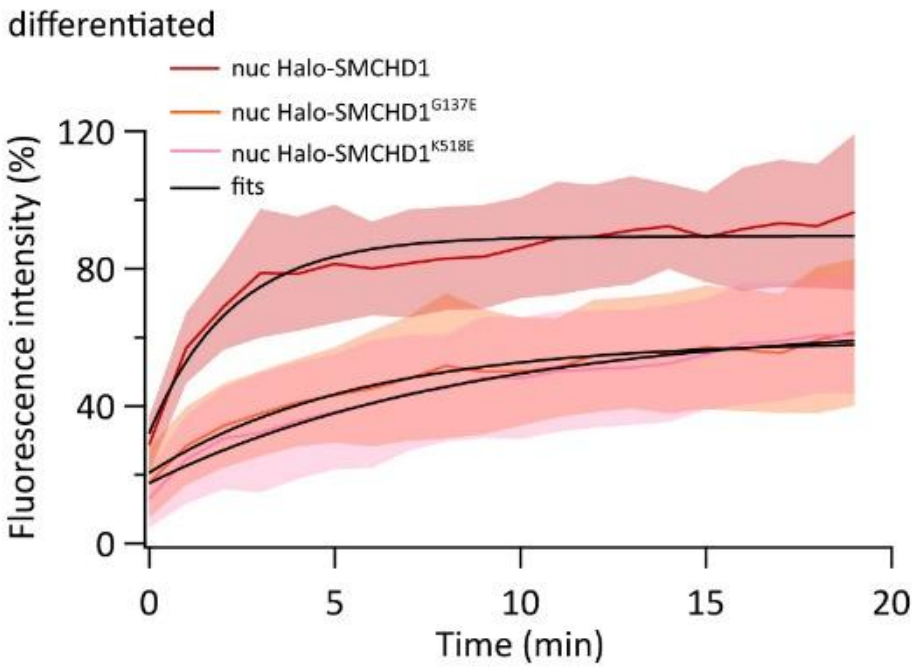

e

	Halo-SMCHD1 Xi domain	Halo-SMCHD1 nucleoplasm	G137E Xi domain	G137E nucleoplasm	K518E Xi domain	K518E nucleoplasm
Immobile population	68%	10%	89%	41%	86%	36%
Lifetime	6.4 min	2.3 min	5.4 min	5.6 min	5.7 min	3.3 min

f

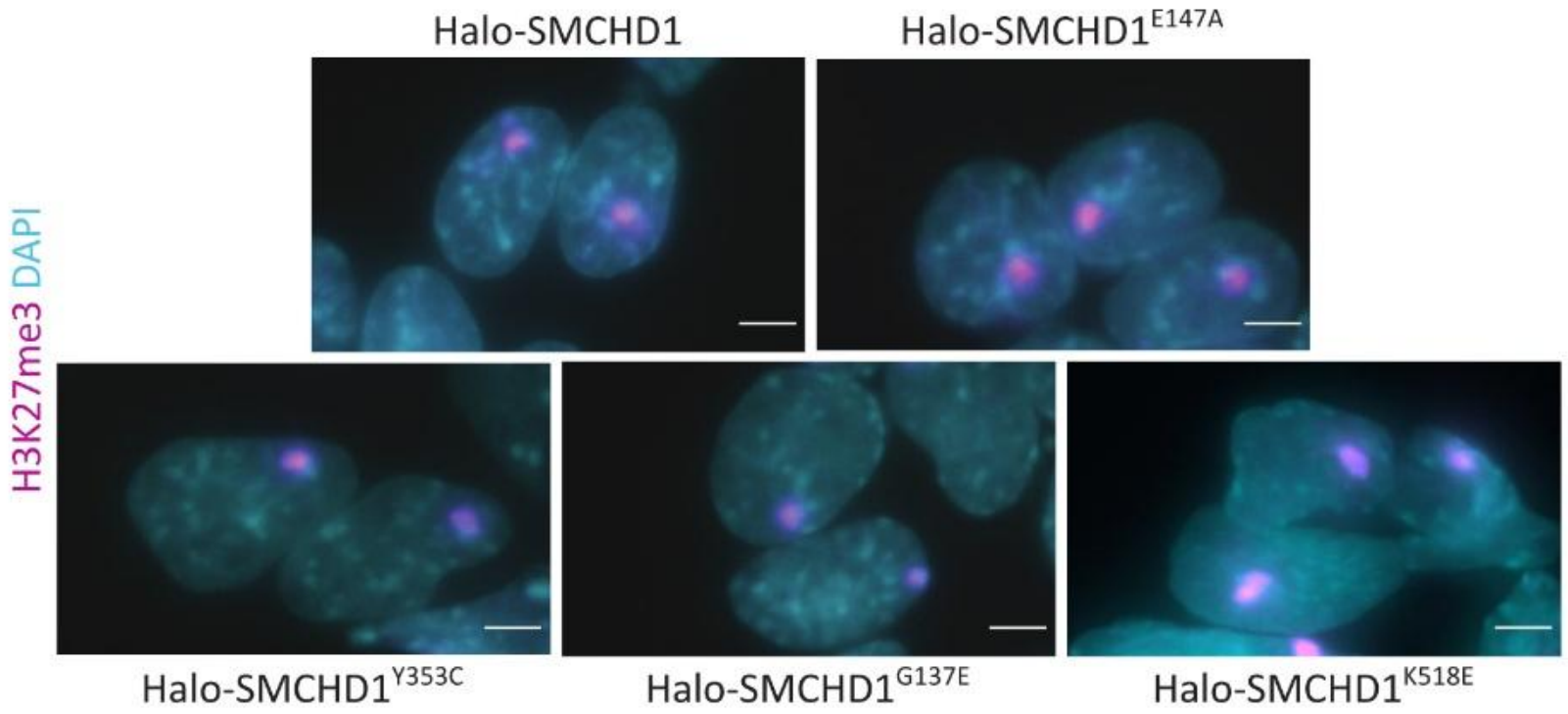

Supplementary Fig. 11 - FRAP analysis of the dynamic behaviour of an SMCHD1 GHKL ATPase gain-of-function mutants

a-b. Representative images from a FRAP experiment using Halo-SMCHD1^{G137E} (a) or Halo-SMCHD1^{K518E} (b) mESCs differentiated for 7 days. The bleached region is marked by a white circle on the bleach snapshot and by a yellow arrow in the post-bleach snapshots. Scale bar - 5μm. c-d. Single exponential fits of the average fluorescence recovery curves over the Xi domain (c) or a random nucleoplasmic region (d). e. Summary of the immobile population and residency values extracted from the fits shown in c-d. f. Immunostaining for H3K27me3 in Halo-SMCHD1, Halo-SMCHD1^{E147A}, Halo-SMCHD1^{Y353C}, Halo-SMCHD1^{G137E} and Hao-SMCHD1^{K518E} mESCs differentiated for 7 days. Images are maximum-intensity projections, scale bar - 5μm. Source data are provided as a Source Data file.

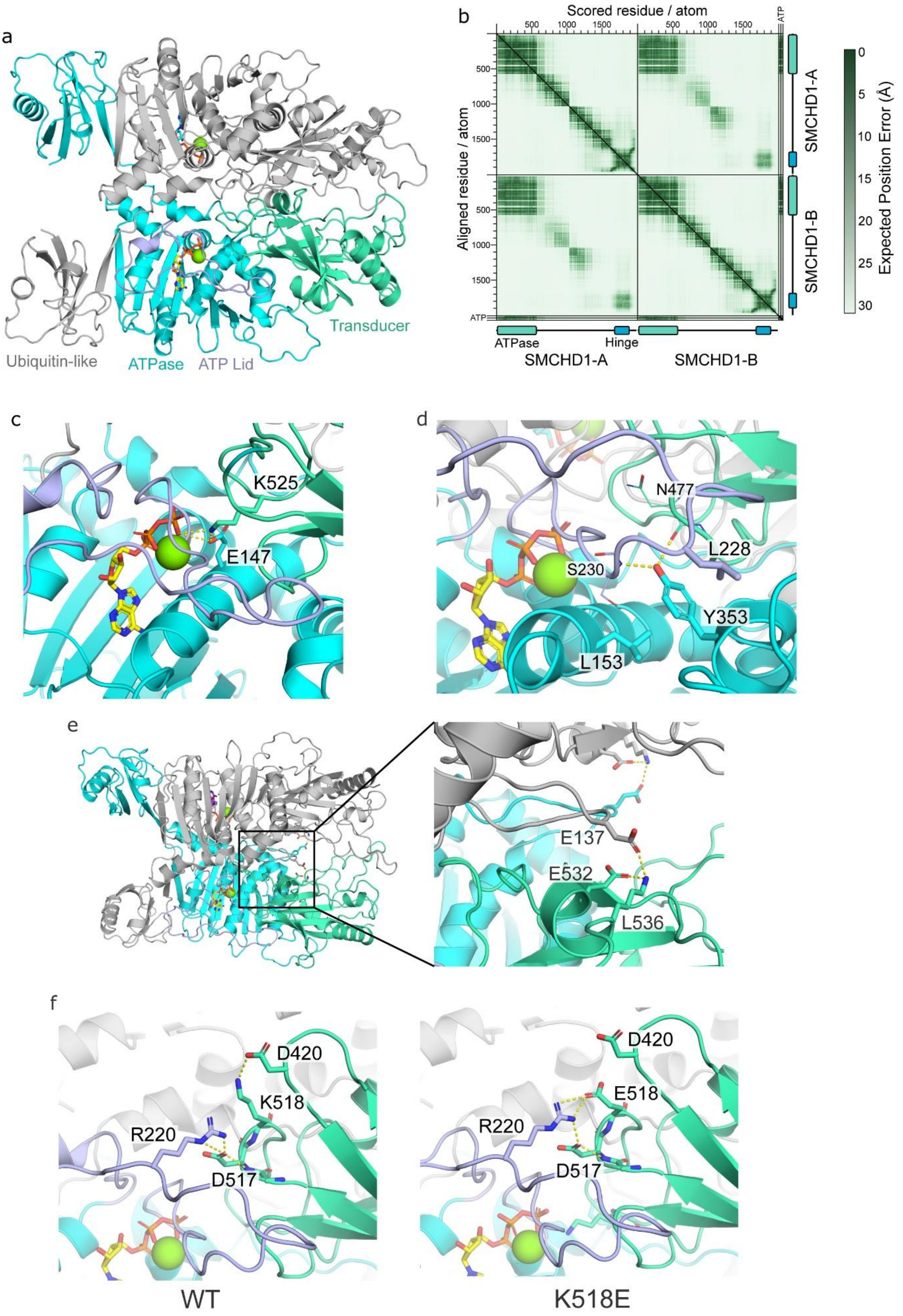

a
Ubiquitin-like
ATPase
ATP Lid
Transducer
b
Scored residue / atom
500
1000
1500
ATP
Aligned residue / atom
SMCHD1-A
SMCHD1-B
ATPase
Hinge
Expected Position Error (Å)
0
5
10
15
20
25
30
c
K525
E147
d
N477
L228
S230
Y353
L153
e
E137
E532
L536
f
D420
K518
R220
D517
E518
WT
K518E

Supplementary Fig. 12 - AlphaFold modelling of the N-terminal part of SMCHD1 with ATP

a. AlphaFold3-predicted structure of the SMCHD1 dimer with one ATP molecule and one Mg^{2+} ion bound per monomer, showing only the ATPase head regions (residues 25-571). One monomer is coloured grey, the other coloured by sub-domain to distinguish the core ATPase (Cyan), ATP lid (Light Blue), and Transducer (Green-Cyan) regions. b. Plot of predicted aligned error (PAE) for the predicted structure of full-length SMCHD1 dimer with ATP and Mg^{2+}. The domain structure of the protein is shown as a schematic to scale alongside. c. Close up of the active site of WT SMCHD1, coloured as in a, showing the position of catalytic residue E147. d. Close up of WT SMCHD1, coloured as in a, showing the local environment around Y353: Y353 forms hydrogen bonds with backbone groups of S230 (ATP lid) and N477 (transducer domain) and hydrophobic interactions with sidechains in the core ATPase domain (L153) and ATP lid (L228). e. Predicted structure of G137E mutant SMCHD1, showing only the head regions (residues 25-571) and coloured as in a. Close-up view shows dimer interface salt bridges formed between E137 and L536 on the opposing monomer. f. Predicted structures of WT (left) and K518E (right) SMCHD1, coloured as in a, showing different salt bridges formed between the ATP lid and transducer regions: K518 (transducer domain) forms a salt bridge with D420 (transducer domain), whereas E518 contacts R220 (ATP lid)

Table S1: Sequences of sgRNAs used for CRISPR-Cas9 targeting

Target sequence	Sequence (5'→3'; PAM sequence in bold)	Source
SMCHD1_N-terminus	GAAAAAAAGAGAAGGCCCTG**CGG**	This study
SMCHD1_C-terminus	GTCTCTTTCAGAAGGCCAAA**AGG**	This study
Gt(ROSA)26Sor	CGCCCATCTTCTAGAAAGAC**TGG**	This study
LRIF1_exon1	GAGGAAAAGTCAGGCTCGCGC**TGG**	This study
LRIF1_exon4	TGTCTTCCCTGGTTGTACTA**TGG**	This study
LRIF1_C-terminus	ATCCAGTGGGGATCCCTGAC**AGG**	This study
SMCHD1_G137	TATGAGTATTATGCGAGTGA**AGG**	This study
SMCHD1_E147	AACTCTGCAAGAGCAAATGC**TGG**	This study
SMCHD1_Y353	AATATTGTTGAAAGGTCCAA**TGG**	This study
SMCHD1_K518E	AGGAGCACACCAGTCAAAGC**TGG**	This study

Table S2: Summary of XX mESC lines derived in this study and associated screening strategies

Cell line	Screening strategy
Halo-SMCHD1	PCR, SS, WB and live imaging
SMCHD1-Snap	PCR, SS, WB and live imaging
LRIF1 KO	PCR, SS and WB
LRIF1-Halo	PCR, SS, WB and live imaging
Halo-SMCHD1^{G137E}	PCR followed by RE digestion, SS

Halo-SMCHD1^{E147A}	PCR followed by RE digestion, SS
Halo-SMCHD1^{Y353C}	PCR followed by RE digestion, SS
Halo-SMCHD1^{K518E}	PCR followed by RE digestion, SS
H2B-Halo	Live imaging
Halo-3xNLS	Live imaging

Table S3: List of primers used for screening of colonies from 96well plate

Primer	Sequence 5'→3'
SMCHD1_N-terminus_tag_FW	GGTCACGGAAGGAGGCTTGG
SMCHD1_N-terminus_tag_RV	CATCACCTCCCTGAACCTGAGGTAC
LRIF1_KO_FW	GCCGCCCTCTCAACTATCTC
LRIF1_KO_RV	GTTCTGGTGGTGTCACTGGA
LRIF1_Halo_FW1	TGTAAATAAACCAGCTTGTTCTCC
LRIF1_Halo_RV1	AAATTCTGGCCATTCGTCCCA
LRIF1_Halo_FW2	TGGGACGAATGGCCAGAATTT
LRIF1_Halo_RV2	CTTACAGCTTACTTCATAACTTGG
SMCHD1_G137E_FW	GAGGCTGAGGCAGGAAGGTAAGTTTG
SMCHD1_G137E_RV	CTCTGAAGTCCAGTGTGCAGGACTGC
SMCHD1_E147A_FW	GTCCAAGGTCTGCCTGGGTTACAAAG

SMCHD1_E147A_RV	CAATAGGCTCACTCATATCACTGGTG
SMCHD1_Y353C_FW	CAGTAAGCTACCAAAGATGATTCCTG
SMCHD1_Y353C_RV	GACATGACATTCCTCTGCGTGTTTGG
SMCHD1_K518E_FW	GATGTTAAGAATTGAGATGTCCCGTTGG
SMCHD1_K518E_RV	GGAGATGCTAGATGACACCGAAGTTCC

Table S4: Summary of no. of movies/regions analysed for each cell line using SPT

Cell line	Timepoint	No. of analysed movies/regions
Halo-SMCHD1	Undifferentiated (24h doxycycline)	60 movies, 343 Xi/random regions
	Differentiated (7 days)	64 movies, 307 Xi/random regions
LRIF1 KO	Undifferentiated (24h doxycycline)	64 movies, 272 Xi/random regions
	Differentiated (7 days)	66 movies, 235 Xi/random regions
Halo-SMCHD1^{E147A}	Undifferentiated (24h doxycycline)	64 movies, 262 Xi/random regions
	Differentiated (7 days)	66 movies, 242 Xi/random regions
Halo-SMCHD1^{Y353C}	Undifferentiated (24h doxycycline)	66 movies, 246 Xi/random regions
	Differentiated (7 days)	66 movies, 250 Xi/random regions
Halo-SMCHD1^{G137E}	Undifferentiated (24h doxycycline)	64 movies, 262 Xi/random regions
	Differentiated (7 days)	66 movies, 242 Xi/random regions
Halo-SMCHD1^{K518E}	Undifferentiated (24h doxycycline)	63 movies, 251 Xi/random regions
	Differentiated (7 days)	61 movies, 218 Xi/random regions
H2B-Halo	Undifferentiated (24h doxycycline)	22 movies
Halo-3xNLS	Undifferentiated (24h doxycycline)	20 movies

Table S5: Antibodies used for Western Blot

Antibody	Host	Source	Dilution
SMCHD1	rabbit	Made in house	1:1000
HDAC3	rabbit	Abcam, ab32369	1:1000
METTL3	rabbit	Abcam, ab195352	1:10000
CIZ1	rabbit	Gift from D. Coverley	1:1000
LRIF1	rabbit	Proteintech, Cat. 26115-1-AP	1:5000
