# Supplementary material for: Selective interaction of SMCHD1 with chromatin is governed by LRIF1 and SMCHD1 ATPase activity
Source: Nat Commun. 2026 Jun 16;17:7636. doi: 10.1038/s41467-026-74427-9 (PMC13434421; doi:10.1038/s41467-026-74427-9)
Supplement: Supplementary file 2 [file 41467_2026_74427_MOESM2_ESM.pdf]

nature portfolio

Corresponding author(s): Neil Brockdorff

Last updated by author(s): Mar 13, 2026

Reporting Summary

Nature Portfolio wishes to improve the reproducibility of the work that we publish. This form provides structure for consistency and transparency in reporting. For further information on Nature Portfolio policies, see our Editorial Policies and the Editorial Policy Checklist.

Statistics

For all statistical analyses, confirm that the following items are present in the figure legend, table legend, main text, or Methods section.

n/a	Confirmed	
☐	☒	The exact sample size (*n*) for each experimental group/condition, given as a discrete number and unit of measurement
☐	☒	A statement on whether measurements were taken from distinct samples or whether the same sample was measured repeatedly
☐	☒	The statistical test(s) used AND whether they are one- or two-sided<br>*Only common tests should be described solely by name; describe more complex techniques in the Methods section.*
☒	☐	A description of all covariates tested
☒	☐	A description of any assumptions or corrections, such as tests of normality and adjustment for multiple comparisons
☐	☒	A full description of the statistical parameters including central tendency (e.g. means) or other basic estimates (e.g. regression coefficient) AND variation (e.g. standard deviation) or associated estimates of uncertainty (e.g. confidence intervals)
☐	☒	For null hypothesis testing, the test statistic (e.g. *F*, *t*, *r*) with confidence intervals, effect sizes, degrees of freedom and *P* value noted<br>*Give P values as exact values whenever suitable.*
☒	☐	For Bayesian analysis, information on the choice of priors and Markov chain Monte Carlo settings
☒	☐	For hierarchical and complex designs, identification of the appropriate level for tests and full reporting of outcomes
☒	☐	Estimates of effect sizes (e.g. Cohen's *d*, Pearson's *r*), indicating how they were calculated

Our web collection on statistics for biologists contains articles on many of the points above.

Software and code

Policy information about availability of computer code

Data collection	Microscopy mages were acquired with an Olympus IX83 microscope, Olympus SoRa spinning disc confocal or a custom TIRF/HILO microscope. Sequencing data for ChrRNA-seq were generated using the Illumina NextSeq 500 platform.
Data analysis	Bowtie2 (2.3.2), SAMtools (1.16.1), STAR (v2.4.2b & 2.7.9a), SNPsplit (0.4.0dev), IGV (2.17.1), Subread (1.5.2), Picard tools (2.25.0 & 3.0.0), deeptools (3.5.5), bedtools (v2.27.1), Python (3.8.10), R (4.2.1), tidyverse (2.0.0), ImageJ through Fiji (Schindelin et al., 2012), Matlab 2013b (Mathworks), Spot-ON v.1 (Hansen et al., 2018); Codes used for the Xi-specific SPT analysis are available via GitHub at https://github.com/aleks-szczure/Constantinescu-et-al.-2026-NATCOM/

For manuscripts utilizing custom algorithms or software that are central to the research but not yet described in published literature, software must be made available to editors and reviewers. We strongly encourage code deposition in a community repository (e.g. GitHub). See the Nature Portfolio guidelines for submitting code & software for further information.

Data

Policy information about availability of data

All manuscripts must include a data availability statement. This statement should provide the following information, where applicable:

- Accession codes, unique identifiers, or web links for publicly available datasets
- A description of any restrictions on data availability
- For clinical datasets or third party data, please ensure that the statement adheres to our policy

All the raw sequencing and key processed data are available at the Gene Expression Omnibus (the accession number: GSE295772, token for reviewers:

krqpiameltudjoh and mpgdqacsznirhoh). The mouse genome (mm10) sequence and gene annotation were downloaded from UCSC genome browser (https://hgdownload.soe.ucsc.edu/downloads.html). The whole genome collections of SNP and short indel variants for mouse strains 129S1 and Cast/EiJ (mpg.v5) was downloaded from mouse genome project (https://www.sanger.ac.uk/data/mouse-genomes-project/). Localised single molecule positions from single particle tracking experiments can be provided upon request.

Research involving human participants, their data, or biological material

Policy information about studies with human participants or human data. See also policy information about sex, gender (identity/presentation), and sexual orientation and race, ethnicity and racism.

Reporting on sex and gender	N/A
Reporting on race, ethnicity, or other socially relevant groupings	N/A
Population characteristics	N/A
Recruitment	N/A
Ethics oversight	N/A

Note that full information on the approval of the study protocol must also be provided in the manuscript.

Field-specific reporting

Please select the one below that is the best fit for your research. If you are not sure, read the appropriate sections before making your selection.

[x] Life sciences [ ] Behavioural & social sciences [ ] Ecological, evolutionary & environmental sciences

For a reference copy of the document with all sections, see nature.com/documents/nr-reporting-summary-flat.pdf

Life sciences study design

All studies must disclose on these points even when the disclosure is negative.

Sample size	No statistical methods were used to predetermine sample size for microscopy and sequencing analysis.<br>For microscopy, sample sizes were determined based on previous studies using similar techniques to enable statistical analysis and reproducibility.<br>For chRNA-seq, 2 or 3 independent repeats for a single clone were carried out.
Data exclusions	No data were excluded for the analysis.
Replication	The number of biological replicates are indicated in the text, figure legend, or method section.
Randomization	For tissue culture based experiments, all wells in each biological replicate were split from the same batch of cells and randomly divided for each type of experiment.
Blinding	All the imaging experiments were performed and analysed by a single person and therefore were not blinded. For chRNA-seq, analysis was all performed by software programs/algorithms

Reporting for specific materials, systems and methods

We require information from authors about some types of materials, experimental systems and methods used in many studies. Here, indicate whether each material, system or method listed is relevant to your study. If you are not sure if a list item applies to your research, read the appropriate section before selecting a response.

Materials & experimental systems

n/a	Involved in the study	
[ ]	[x]	Antibodies
[ ]	[x]	Eukaryotic cell lines
[x]	[ ]	Palaeontology and archaeology
[x]	[ ]	Animals and other organisms
[x]	[ ]	Clinical data
[x]	[ ]	Dual use research of concern
[x]	[ ]	Plants

Methods

n/a	Involved in the study	
[x]	[ ]	ChIP-seq
[x]	[ ]	Flow cytometry
[x]	[ ]	MRI-based neuroimaging

Antibodies

Antibodies used	anti-SMCHD1 (made in-house), anti-HDAC3 (Abcam, ab32369), anti-METTL3 (Abcam, ab195352), anti-CIZ1 (gift from D. Coverley), anti-LRIF1 (Proteintech, 26115-1-AP)
Validation	Antibodies are verified by manufacturers or previous /current publications using knockouts.<br><br>anti-SMCHD1 1:1000 (https://doi.org/10.1038/s41467-018-07907-2)<br>anti-HDAC3 1:1000 (https://www.abcam.com/en-us/products/primary-antibodies/hdac3-antibody-y415-ab32369)<br>anti-METTL3 1:10000 (https://www.abcam.com/en-us/products/primary-antibodies/mettl3-antibody-epr18810-ab195352)<br>anti-LRIF1 1:5000 (https://www.ptglab.com/products/C1orf103-Antibody-26115-1-AP.htm?srsltid=AfmBOorumktwFGlT0ZhF8ERiPd5yFy2_i8w7f7OeRCjj-JgbmGL-znpa)<br>anti-CIZ1 1:1000 (https://doi.org/10.1126/science.abe7500)

Eukaryotic cell lines

Policy information about cell lines and Sex and Gender in Research

Cell line source(s)	All mouse embryonic stem cells (mESCs) used in this study are female, derived from the F1 2–1 XX mESC line (129/Sv-Cast/Ei), a gift from by J. Gribnau. Using this background, we developed dox-inducible endogenous Xist cell line targeted to the 129S allele (iXist-ChrX).<br>We created N-terminal Halo-tagged SMCHD1 and introduced a eGFP-CIZ1 transgene in the Rosa26 locus in the iXist-ChrX background. We then further modified this line to knock-out LRIF1 or introduce point mutations in SMCHD1. We also generated C-terminal Snap-tagged SMCHD1 in the iXist-ChrX background; C-terminal Halo-tagged LRIF1 and introduced the eGFP-CIZ1 transgene in the Rosa26 locus in the iXist-ChrX background; randomly introduced a transgene encoding for Halo-tagged H2B or Halo-NLS in the iXist-ChrX background.
Authentication	All engineered cell lines were validated at the genomic level, using PCR and Sanger sequencing, and at the protein level, using Western blot analysis and imaging.
Mycoplasma contamination	All the cell lines are regularly tested for mycoplasma contamination, and confirmed to be negative.
Commonly misidentified lines (See ICLAC register)	No commonly misidentified lines were used in this work.

Plants

Seed stocks	Plants are not used in this study.
Novel plant genotypes	Plants are not used in this study.
Authentication	Plants are not used in this study.
