# Supplementary material for: Selective interaction of SMCHD1 with chromatin is governed by LRIF1 and SMCHD1 ATPase activity
Source: Nat Commun. 2026 Jun 16;17:7636. doi: 10.1038/s41467-026-74427-9 (PMC13434421; doi:10.1038/s41467-026-74427-9)
Supplement: Supplementary file 3 [file 41467_2026_74427_MOESM3_ESM.pdf]

nature portfolio

Peer Review File

Selective interaction of SMCHD1 with chromatin is governed by LRIF1 and SMCHD1 ATPase activity

Corresponding Author: Professor Neil Brockdorff

This file contains all reviewer reports in order by version, followed by all author rebuttals in order by version.

Version 0:

Reviewer comments:

Reviewer #1

(Remarks to the Author)
This manuscript delivers a thorough and methodologically robust analysis of how SMCHD1 is recruited to chromatin during X-chromosome inactivation (XCI). Through the integration of live-cell single-particle tracking (SPT), FRAP, and CRISPR-engineered ATPase mutants, the authors convincingly show that LRIF1 serves as a critical loading factor for SMCHD1 and that ATP hydrolysis fine-tunes its chromatin-binding specificity. These results offer valuable mechanistic insights into a long-standing question in epigenetic gene silencing and chromosome organization. The study represents a notable advance in our understanding of chromatin regulation, with broad implications for the fields of XCI, 3D genome architecture, and the biology of SMC-family proteins. The work is technically exceptional, conceptually solid, and highly relevant. I enthusiastically recommend publication in Nature Communications, provided the authors address the minor to moderate revisions detailed below.

Major Comments
1. Although this study makes a significant contribution toward understanding the recruitment mechanism of SMCHD1 to the X chromosome, the downstream molecular mechanism by which SMCHD1 induces gene silencing during X-chromosome inactivation (XCI) remains incompletely understood. In particular, the interplay between SMCHD1 and Xist RNA should be more extensively discussed in the context of XCI initiation and maintenance.
2. The mechanism by which LRIF1 contributes to the stepwise recruitment of SMCHD1 to the Xi—beyond simply recognizing H3K9me3—should be clarified. XCI progresses through defined stages of differentiation, and it would be informative to address whether LRIF1 function is dynamically regulated across these stages.
3. The mechanistic interpretation of the phenotypic differences observed in the ATPase-deficient mutants (E147A and Y353C) requires deeper analysis. It is currently unclear why mutations that both abrogate ATP hydrolysis exhibit divergent chromatin-binding behaviors. A structural or biochemical rationale would strengthen this point.
4. The use of eGFP-CIZ1 for Xi identification should be critically evaluated in terms of potential overexpression artifacts. It would be helpful if the authors could clarify whether eGFP-CIZ1 is expressed at near-endogenous levels and whether it affects Xi chromatin structure or SMCHD1 dynamics.
5. The mechanism by which LRIF1 and ATP hydrolysis jointly contribute to gene silencing—particularly at specific loci—needs further elaboration. For example, are certain X-linked genes silenced via higher-order chromatin structure changes that are dependent on SMCHD1 loading? Allele-specific chromatin topology or long-range interaction data could strengthen this argument.
6. The authors should discuss how SMCHD1-mediated gene silencing differs from canonical SMC proteins such as cohesin or condensin. Specifically, how does SMCHD1's chromatin residency and ATPase-dependent specificity contribute uniquely to silencing, as opposed to loop extrusion or chromatin compaction?

Minor Comments
1. Extended Data Fig. 1 caption should be revised. Panel B is not a representative image but rather a comparison between Halo-SMCHD1 and the parental iXist-ChrX line. Similarly, the box plot in panel D is not an image but a quantification. The current figure legend does not accurately describe the panels and should be corrected.
2. The histone H3 band in Extended Data Fig. 2e appears distorted or overexposed. The figure should be replaced or repeated for clarity.
3. The background contrast in Extended Data Figures 3d, 4f, 7a–b, and 9a is inconsistent with other figures. These images are overly bright and should be adjusted to match the visual tone of the rest of the dataset.

4. The differential regulation of SMCHD1 recruitment to the Xi versus autosomal loci should be discussed. While LRIF1 appears to play a central role in both contexts, it remains unclear whether the recruitment mechanism is identical or context-dependent.
5. The ATPase activities of the SMCHD1 mutants should be directly quantified in this study, rather than inferred solely from previous publications. Furthermore, the authors should consider whether LRIF1 may influence SMCHD1's ATPase activity, and if so, propose a model for how LRIF1 coupling may regulate chromatin loading through the ATPase domain.

Reviewer #2

(Remarks to the Author)
The study uses live imaging to study the function of SMCHD1 during X inactivation. SMCHD1 is an important protein for X inactivation and it is mutated in two human diseases with no current cures. While SMCHD1 has a GHKL ATPase domain, the function of this weak ATPase is unknown. This study uses ATPase mutants to explore the functional significance of ATP hydrolysis. As many mutations in the ATPase have been found in the ATPase domain in the human diseases, this study is timely and important.
Through live imaging, this study provides new insights into the diffusion rates and binding dynamics of SMCHD1 in vivo, which could not be gained previously from experiments with fixed cells. The authors found that SMCHD1's binding to heterochromatin and Xi is dependent on LRIF1. The authors then went on to investigate the function of ATPase activity on SMCHD1 function and performed experiments on 3 ATPase mutants. Interestingly, while both ATPase-deficient Y353C and E147A reduced SMCHD1 localization on Xi, they do so through different mechanisms. The hyperactive G137E mutant showed increased binding to Xi and greater Xi compaction. The authors concluded that ATP hydrolysis is required for selective enrichment of SMCHD1 at its target chromatin regions, which impacts its ability to silence Xi.
The study is well done with extensive quantitative analysis. My main concerns are with the changes in stability of SMCHD1 and with the interpretation of the ATPase mutant data.

Major concerns
In LRIF1 KO, SMCHD1 protein levels are reduced (Fig 2c). Why is that? Is SMCHD1 less stable when it is not bound to chromatin? The ATPase mutants also show decreases in protein stability. Can the stability be (anti-)correlated to the level of chromatin binding?
While the SMCHD1 localization changes are very clear for LRIF1 KO, there is also a reduction in total SMCHD1 levels. How do you know whether the difference is due to lower levels of SMCHD1 or due to recruitment? I have similar concerns with the E147A and Y353C mutants. Related to that, if WT SMCHD1 levels decrease in the presence of LRIF1, will that also change its chromatin localization?
Figure E4c. Why does SMCHD1 JF549 get brighter on day 3?
Figure 4d. Why do independent genes also show less repression in E147A?
The distribution of Halo SMCHD1 and Y353C in Figure 5c looks different (less bound for Y353C), but the quantification in d shows that they are the same. Please check that the quantification is right.
Does the selection of “random” nucleoplasm take into consideration the locations of PCH, for example through Hoescht staining? I would imagine SMCHD1's dynamics on PCH to be different from nucleoplasm. Authors should be clear in their description.
The G137E mutant is more bound to the Xi and the Xi is more compacted. Why is there no change in Xi expression? Can the authors speculate on the impact of the G137E, particularly what it may mean for understanding BAMS?
Previous assessments of ATPase activity were performed with just the ATPase domain alone. An assumption has been that the ATPase activity of the whole protein is similar to that of the domain alone. While that is probably true for the loss of function mutants E147A and perhaps Y353C, it is not so clear whether G137E's ATPase activity will be increased to the same extent in the full length protein, as interactions with other parts of the protein may influence ATPase activity. This assumption should be included in the manuscript.
The authors used 3 ATPase mutants to derive a relationship between ATP hydrolysis and SMCHD1 Xi/ chromatin localization. However it is odd that the 2 LOF mutants show different binding dynamics. E147A is less mobile in nucleoplasm while Y353C is more mobile on Xi. As such, it's not a simple relationship of less ATP hydrolysis = less binding. While the data of the mutants is interesting, the link between ATPase activity and binding dynamics is not very strong. The authors suggested that Y353C may result in the protein disengaging from chromatin rapidly following initial binding. Is there any structural evidence based on the locations of the mutations that this could happen? In my opinion, more mutants are needed to clarify the relationship between ATP hydrolysis, protein unloading and Xi binding. Alternatively, tone down on the relationship between ATP hydrolysis and chromatin binding.
The authors suggest that changes in ATPase activity directly influence the dynamics of binding to target sites on chromatin. However the data can also be interpreted that the ATPase activity affects SMCHD1's interactions with other adapter proteins (LRIF1 or other unknown proteins) that bring SMCHD1 to chromatin. Please address this possibility.
In light of the last 2 points, I suggest that the authors tone down the relationship between ATP hydrolysis and direct SMCHD1 recruitment to chromatin in the model, perhaps adding question marks to parts which are not so certain.

Minor concerns
Abstract: microphthalmia is spelled incorrectly.
Line 73. It is debatable whether DUX4 expression is relevant for BAMS. And it is not clear how gain-of-function mutations in GHKL ATPase domain can lead to less repression of DUX4. This reviewer suggests to not draw this link between BAMS mutations and DUX4.
Figure E1. The descriptions of b and d are reversed.

Random Halo-SMCHD1 should be added to the quantification graphs of % bound population from single particle tracking experiments (eg. 3f,5g,j,6i).
“This may seem counterintuitive given the absence of enrichment of the mutant protein over the Xi (Figure 4b, Extended Data Figure 5e), but it is key to note that the bound population of SMCHD1E147A in random regions is very similar to that seen at the Xi, both in undifferentiated and differentiated cells (Figure 5e-g).” I understand what is counterintuitive but struggle to understand the explanation. Please rephrase.
Line 387. Is there any evidence that G137E disfavours ADP release? Previous in vitro results show more ADP production with G137E (PMID: 29748383), which should correspond to increased, not reduced, ADP release.

Reviewer #3

(Remarks to the Author)
Constantinescu et al. used live-cell and single-molecule imaging to investigate SMCHD1 interactions with chromatin and its function in epigenetic silencing. They also engineered mutations in the GHKL ATPase domain of SMCHD1 and investigated their effects on SMCHD1 chromatin enrichment Xi gene repression and chromatin compaction.
The results provide interesting data addressing the selective enrichment of SMCHD1 at specific chromatin regions and gene silencing on the Xi. Nevertheless, there are a number of aspects that need to be addressed the clarify things and better support the authors claims, as listed below.

1. It is not correct that only loss-of-function mutations in SMCHD1 are present in FSHD. Indeed, the G137E gain-of-function mutation is associated to both FSHD and BAMS (PMID: 28067909).

2. Lines 207-208: Contrary to the authors claims, reference 18 clearly show that LRIF1 depletion in differentiated cells affects SMCHD1 association to some Xi regions.

3. In Figure 3g and 4b, the mutants behave similar to the WT (showing similar increase in enrichment over Xi) for the first 5 days of differentiation and then they plateau, while the WT keep increasing at day 6 and 7. Hence, SMCHD1 enrichment over Xi during differentiation appears to have a bimodal mechanism: an initial one independent by LRIF1 and unaffected by the E147A or Y353C mutation, followed by a later burst in Xi enrichment which instead is affected by the above conditions. Moreover, SMCHD1 dynamic behavior across the Xi domain is unaffected by the E147A or Y353C mutation (Figure 5e-j). It is difficult to reconcile all of this just with altered GHKL ATPase domain activity.

4. The authors speculate that an interaction between prebound and newly recruited SMCHD1 could be involved in the protein enrichment over Xi. Is any of the SMCHD1 mutants used in the study affecting Xi recruitment of the WT SMCHD1? What is the mechanism responsible for the burst in SMCHD1 enrichment over Xi after day 5?

5. The immunoblot of Extended Data Figure 5c,d show that the E147A and Y353C mutants significantly (it looks like 50%) reduce the SMCHD1 levels, which could affect the results of Figure 4. This caveat should be minimally discussed by the authors, better if experimentally addressed.

6. To better support their claims about the G137E mutant, the authors should test at least another mutation increasing SMCHD1 catalytic activity such as A134S, S135C, E136G or (even better because located in a different part of the protein) K518E.

7. There is clearly a different behavior of SMCHD1/LRIF1 in human and mouse cells and in ES compared to somatic cells. For example, LRIF1 does not affect SMCHD1 levels nor its recruitment to the D4Z4 locus when knock-out/knock-down in human somatic cells (PMID: 37380887), which should be discussed.

8. For a number of graphs/quantifications (for example all Mean Xi intensity) no statistics is provided. This will be useful and in particular required whenever the authors claim there is a difference.

9. The study is mostly descriptive as the claimed advance in mechanistic understanding of SMCHD1 function comes from correlative data.

MINOR

A. SMCHD1 has a general relevance in FSHD, given that it is also a gene modifier in FSHD1. Hence, in the manuscript, the authors could change “FSHD2” in FSHD”. Nevertheless, the molecular pathogenesis of BAMS is unclear and there is no clear indication that aberrant epigenetic regulation of D4Z4 or altered DUX4 contribute to BAMS. Hence, the authors should modify the abstract accordingly.

B. I’m not sure that the iXist-ChrX can be defined an interspecific line since M.m. domesticus and M.m. castaneus are not species. Indeed, they can interbreed and produce viable offspring.

C. The legend to Extended Data Figure 6 and 7 is very confusing since they are referring to panels of other Figures, i.e. e. In red, same as in Figure 3a. In green, same as in Figure 5e. f. In blue, same as in Figure 3b, etc.

Reviewer #4

(Remarks to the Author)
A potentially interesting study for understanding the detailed mechanism of SMCHD1 recruitment to chromatin (pericentromeric heterochromatin and the inactive X) in early development.
The authors created and validated a model system in which they monitored SMCHD1 subnuclear localisation by imaging in live cells. Their findings are the following:
-SMCHD1 localisation to pericentromeric heterochromatin and Xi is visualised in live cells
-LRIF1 localisation to PHC and Xi is visualised in live cells
-SMCHD1 localisation to PHC and Xi depends on LRIF1
-SMCHD1 ATP ase activity is essential for chromatin recruitment of SMCHD1

The study is well-designed, and the experimental data are well-analysed, with comparisons of different analysis methods sometimes included. The experimental data support the conclusions.
However, the study does not provide substantial new biological insights into SMCHD1 chromatin recruitment, which would qualify it for publication in Nature Communications.
My suggestion is to reject the manuscript and resubmit it to a lower impact journal e.g Elife.

Remarks:

1. SMCHD1 G137E mutation was identified not only in BAMS but also in FSHD 2 (Lemmers et al JMG 2019)

2. Extended Figure 1: correct labelling of the panels (panel b is panel d)

3. LRIF1 KO clones: somatic KO of LRIF1 did not show reduced SMCHD1 (Sikrova et al 2023). Authors show only one clone for SMCHD1 blot. Show SMCHD1 levels in all 6 LRIF1 KO clones included in Extended figure 2 panel d.

4. The authors claim that LRIF1 recruits SMCHD1 to PCH, supporting their claim by showing the missing SMCHD1 signal at PCH in Figure 2, panel B. However, this sample has a very low level of SMCHD1, so it is possible that at this low level, SMCHD1 is not detectable.

5. Extended figure 2 panel E: it seems that in the LRIF1 KO sample the 2 short isoforms of LRIF1 are present, while on panel D these bands are not present. Does panel E show an LRIF1 KO sample?

6. Earlier studies claim that dimerisation of SMCHD1 is essential for chromatin binding. Can G137E, E147A or Y353C mutants form dimers in the described model system?

Version 1:

Reviewer comments:

Reviewer #1

(Remarks to the Author)
The current manuscript is publishable

Reviewer #2

(Remarks to the Author)
The authors have done a tremendous job in the revision. I am very happy that they have included an additional mutant, which strengthened the link between ATPase activity and recruitment to target sites. I recommend accepting the manuscript. Some of the reviewer figures are particularly informative. Reviewer Figure 5 is a very good control and I recommend including it in the manuscript as a supplemental figure. I also suggest to summarize the Alphafold analysis and include it in the manuscript to explain the difference between the 2 LOF mutations.

Reviewer #3

(Remarks to the Author)
The authors satisfactorily addressed my concerns. Congratulation on a nice pice of work!

Reviewer #4

(Remarks to the Author)
The authors of the manuscript “Selective interaction of the protein SMCHD1 with specific chromatin regions is governed by the loading factor LRIF1 and SMCHD1 ATPase activity” provided a thorough and well-constructed rebuttal. Their responses to the reviewers’ comments, along with the inclusion of additional data—particularly the chromatin binding analysis of the K518E gain-of-function SMCHD1 mutant—and substantial contextual revisions, have markedly strengthened the manuscript. I support publishing it in Nature Communications after minor corrections.

1. Raw 70:" A notable case is the gene encoding DUX4 in human, abnormal expression of which results in FSHD2."
DUX4 is expressed not only in FSHD2 but also in FSHD1. I suggest correcting FSHD2 to FSHD.

2. Raw 72: "FSHD2 mutations have also been found in a second factor, LRIF1,"
There is only one LRIF1 mutation published; the mutation should be corrected to mutation.

Open Access This Peer Review File is licensed under a Creative Commons Attribution 4.0 International License, which permits use, sharing, adaptation, distribution and reproduction in any medium or format, as long as you give appropriate credit to the original author(s) and the source, provide a link to the Creative Commons license, and indicate if changes were made.
In cases where reviewers are anonymous, credit should be given to 'Anonymous Referee' and the source.
The images or other third party material in this Peer Review File are included in the article's Creative Commons license, unless indicated otherwise in a credit line to the material. If material is not included in the article's Creative Commons license and your intended use is not permitted by statutory regulation or exceeds the permitted use, you will need to obtain permission directly from the copyright holder.
To view a copy of this license, visit https://creativecommons.org/licenses/by/4.0/

We thank the reviewers for their constructive comments and suggestions. Please find a detailed response below.

REVIEWER COMMENTS

Reviewer #1 (Remarks to the Author):

This manuscript delivers a thorough and methodologically robust analysis of how SMCHD1 is recruited to chromatin during X-chromosome inactivation (XCI). Through the integration of live-cell single-particle tracking (SPT), FRAP, and CRISPR-engineered ATPase mutants, the authors convincingly show that LRIF1 serves as a critical loading factor for SMCHD1 and that ATP hydrolysis fine-tunes its chromatin-binding specificity. These results offer valuable mechanistic insights into a long-standing question in epigenetic gene silencing and chromosome organization. The study represents a notable advance in our understanding of chromatin regulation, with broad implications for the fields of XCI, 3D genome architecture, and the biology of SMC-family proteins. The work is technically exceptional, conceptually solid, and highly relevant. I enthusiastically recommend publication in Nature Communications, provided the authors address the minor to moderate revisions detailed below.

Major Comments

1. Although this study makes a significant contribution toward understanding the recruitment mechanism of SMCHD1 to the X chromosome, the downstream molecular mechanism by which SMCHD1 induces gene silencing during X-chromosome inactivation (XCI) remains incompletely understood. In particular, the interplay between SMCHD1 and Xist RNA should be more extensively discussed in the context of XCI initiation and maintenance.

Indeed, this study is principally concerned with understanding SMCHD1 recruitment mechanisms rather than its downstream function. To address the reviewers point we have expanded our discussion of SMCHD1 in gene silencing and its interplay with Xist RNA in the introduction (lines 58-67). We have also added to the discussion section concerning implications of our analysis of gain of function GHKL ATPase mutants with respect to X-linked gene silencing.

2. The mechanism by which LRIF1 contributes to the stepwise recruitment of SMCHD1 to the Xi—beyond simply recognizing H3K9me3—should be clarified. XCI progresses through defined stages of differentiation, and it would be informative to address whether LRIF1 function is dynamically regulated across these stages.

It has previously been shown that LRIF1 interacts directly with SMCHD1, at its hinge domain, and mediates its association with H3K9me3-marked chromatin (Brideau et al., *Mol Cell Biol.* 2015 PMID: 26391951; Nozawa et al., *Nat Struct Mol Biol* PMID: 23542155). To gain more insight into the relation between LRIF1 and the Xi, we have now carried out a recruitment timecourse using our LRIF1-Halo mESC line and this revealed that LRIF1 undergoes gradual recruitment to the Xi, in a similar manner to that observed for SMCHD1 (Reviewer Figure 1, Figure 1f). This new data highlights a close temporal relationship between these two factors. While in this study, we show that LRIF1 plays a key role in the recruitment of SMCHD1 to the Xi during the establishment of XCI, previous reports have indicated that localization of SMCHD1 over the Xi territory is not affected by removal of LRIF1 in somatic cells (Brideau et al., *Mol Cell Biol.* 2015 PMID: 26391951; Nozawa et al., *Nat Struct Mol Biol* PMID: 23542155). It will be important to better understand the interplay of H3K9me3-

LRIF1-SMCHD1 during establishment of X inactivation, but it is important to note that these questions are hindered by a lack of clear understanding of the exact role of H3K9me3 in XCI in the mouse XCI model. Studies have reported the presence of this mark as blocks over the Xi chromosome (Ichihara et al., *Development* 2022. PMID: 35831949; Keniry et al., *Epigenetics Chromatin.* PMID: 27195021), but its functionality in XCI is still not clear.

Reviewer Figure 1

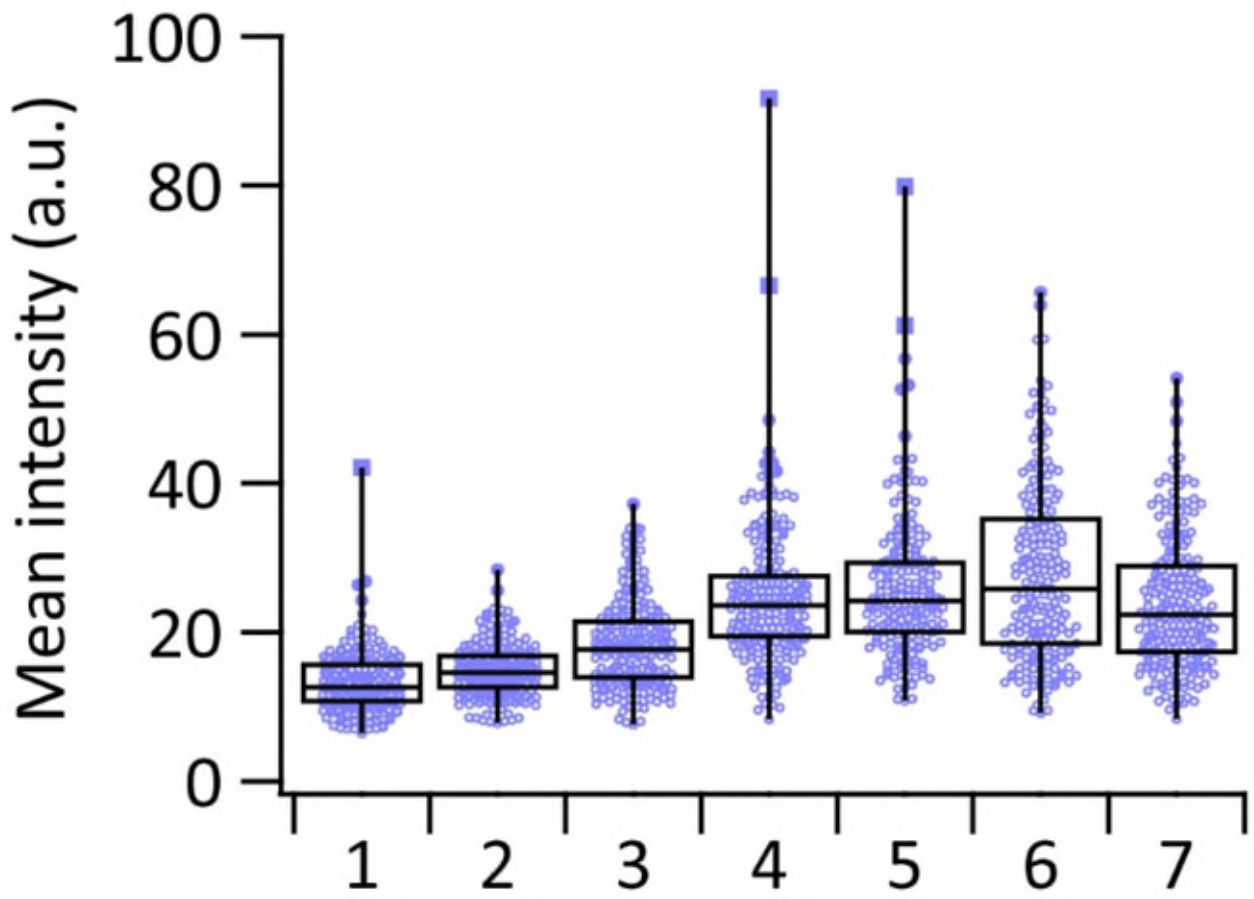

Reviewer Figure 1: Timecourse of LRIF1 recruitment to the Xi chromosome

Quantification of LRIF1 signal underlying CIZ1 domains in the LRIF1-Halo mESC line during 7-day differentiation timecourse. Each dot represents a cell. n = 190-214.

3. The mechanistic interpretation of the phenotypic differences observed in the ATPase-deficient mutants (E147A and Y353C) requires deeper analysis. It is currently unclear why mutations that both abrogate ATP hydrolysis exhibit divergent chromatin-binding behaviors. A structural or biochemical rationale would strengthen this point.

In support of our mechanistic interpretation of the different phenotypes displayed by the ATPase mutants analysed in this study, we have now determined AlphaFold models showcasing how each mutation may affect the interaction of SMCHD1 with ATP and/or ATP hydrolysis itself (Reviewer Figure 2). SMCHD1 interacts with chromatin as a homodimer (Brideau et al., *Mol Cell Biol.* 2015 PMID: 26391951), but AlphaFold does not allow modelling of the entire protein as a dimer due to its large size. We have therefore modelled the N-terminal part of SMCHD1 including the ubiquitin-like fold (UBL) domain, the GHKL ATPase domain and the transducer domain, showing its interaction as a dimer with ATP and Mg^{2+} (Reviewer Figure 2a). Previous studies have shown that the UBL and transducer domain are required for dimerization of the N-terminal part of SMCHD1 in the presence of ATP binding, and the latter is also needed for ATP hydrolysis (Pedersen et al., *Commun Biol.* 2019 PMID: 31312724; Gurzau et al., *Biochem J.* 2021 PMID: 34109974). The E147A mutant retains ability to stably bind ATP as previously shown (Pedersen et al., *Commun Biol.* 2019 PMID: 31312724), but the mutation affects the core catalytic residue which is thought to activate the nucleolytic water molecule, hence disabling the hydrolysis reaction (Reviewer Figure 2b). The mutant protein would, thus be able, to proceed to the initial loosely-engaged chromatin state, but not further to the stably-engaged one as outlined in the model (Figure 8). On the other hand, Y353 is a large residue which displays hydrophobic packing interactions with the core ATPase domain and the ATP lid, which folds

over the catalytic pocket upon ATP binding, and hydrogen bonds with backbone groups in the ATP lid and the transducer domain (Reviewer Figure 2c). The Y353C substitution disrupts these interactions, leading to reduced anchoring of the ATP lid. In turn, this will affect the binding kinetics of ATP and/or the wider conformational changes associated with ATP binding, precluding ATP hydrolysis and, ultimately, causing the observed loss-of-function phenotype. The mutant protein can bind ATP and loosely-engage with chromatin, but the ATP-bound state is not as stable as in the WT or the E147A mutant so $SMCHD1^{Y353C}$ quickly dissociates from chromatin (Figure 8).

Regarding the G137 gain-of-function mutant, G137 sits at the end of a loop in the cavity between the monomers (Reviewer Figure 2d). The substitution to glutamic acid introduces a new salt bridge interaction with K536 of the other monomer, which can lead to stabilisation of the dimer, leading to the observed increase in the bound population of the mutant protein, and it can also increase the energy required to unfold the dimer, release ADP and, thus, release SMCHD1 from chromatin, causing the observed increase in residence time of $SMCHD1^{G137E}$ (Reviewer Figure 2e; Figure 8). We have now included analysis of an additional gain of function mutation, K518 (see reviewer 2 comments). K518 is located in the transducer domain and forms a salt bridge with D420, part of the same domain, while its substitution to glutamic acid leads to formation of a salt bridge with R220, placed in the ATP lid. This could change the conformation and/or dynamics of the ATP lid, stabilising the active site and consequently altering the kinetics of ATP binding and hydrolysis (Reviewer Figure 2f). Thus, this translates then into the observed enhanced chromatin association of $SMCHD1^{K518E}$ (Figure 7,8).

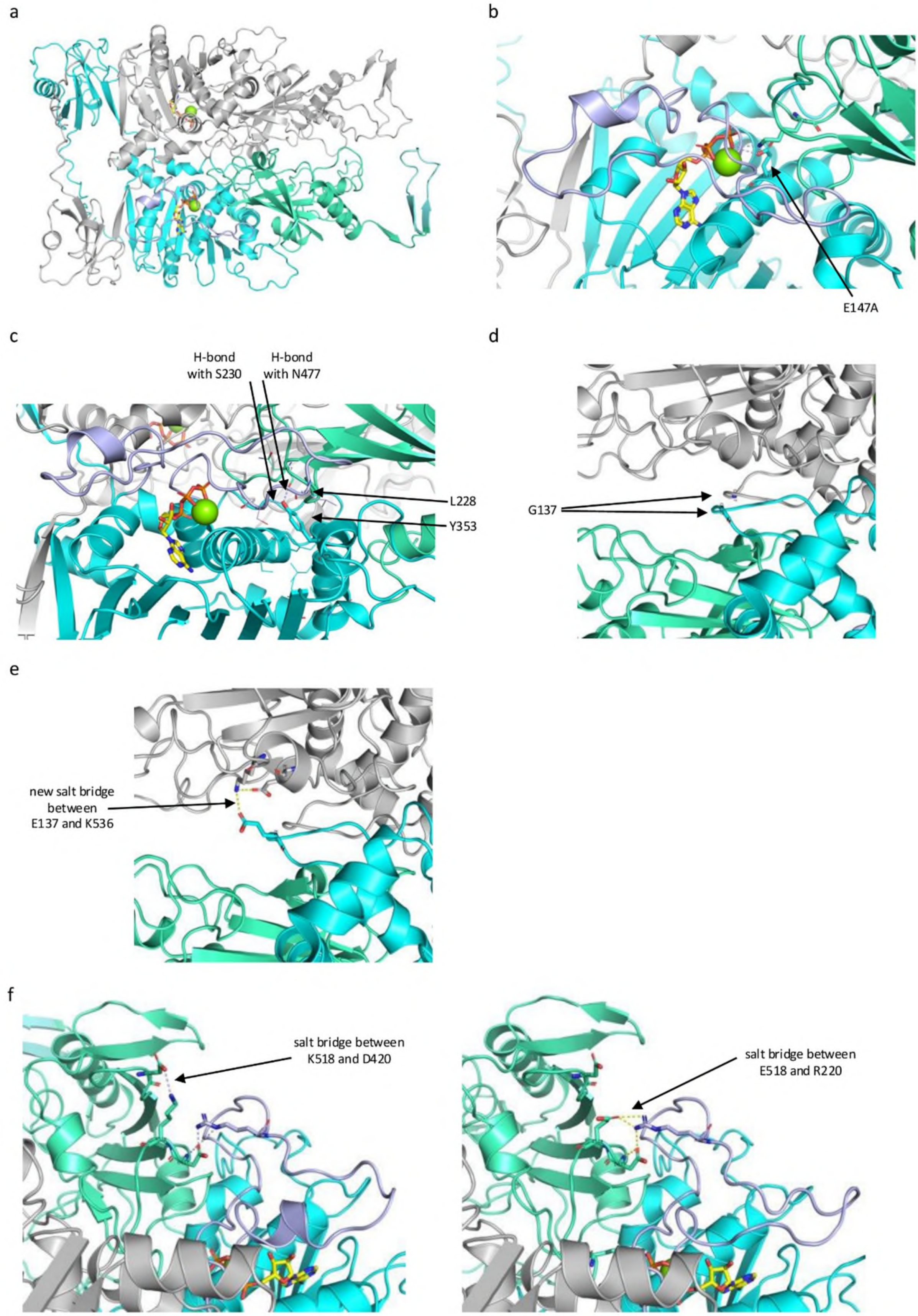

Reviewer Figure 2: AlphaFold modelling of the N-terminal part of SMCHD1 with ATP

a. AlphaFold modelling of the N-terminal part of SMCHD1 including the UBL, GHKL ATPase and transducer domains, shown as a dimer with ATP and Mg^{2+} bound. One dimer is shown in colour (purple – ATP lid, cyan – UBL and GHKL ATPase domains, green – transducer domain) and the other in grey.
b. AlphaFold modelling of the catalytic pockets of SMCHD1 with ATP and Mg^{2+} bound highlighting the key position of residue 147.
c. AlphaFold modelling showing the interactions between Y353 and the core of the ATPase domain, the ATP lid and the transducer domain: hydrogen bonds with S230 (ATP lid) and N477 (transducer domain) and hydrophobic interactions with the core ATPase domain and L228 (ATP lid).
d. AlphaFold modelling showing location of the G137 residue of each monomer
e. AlphaFold modelling showing new salt bridge formed between E137 from one monomer and K536 from the other monomer
f. AlphaFold modelling showing switch from salt bridge between K518 (transducer domain) and D420 (transducer domain) to salt bridge between E518 and R220 (ATP lid) upon K518E substitution

4. The use of eGFP-CIZ1 for Xi identification should be critically evaluated in terms of potential overexpression artifacts. It would be helpful if the authors could clarify whether eGFP-CIZ1 is expressed at near-endogenous levels and whether it affects Xi chromatin structure or SMCHD1 dynamics.

eGFP-CIZ1 is more highly expressed compared to the endogenous level of CIZ1 (Figure 1d). It has been reported that Xist localises to about 50 sites across the Xi domain (Markaki et al., *Cell* 2021 PMID: 34813726; Rodermund et al., *Science* 2021 PMID: 34112668). Previous studies have shown that CIZ1 binds to the E-repeat of Xist (Ridings-Figueroa et al., *Genes Dev.* 2017 PMID: 28546514; Sunwoo et al., *Proc Natl Acad Sci USA* 2017 PMID: 2892396; Markaki et al., *Cell* 2021 PMID: 34813726), functioning to ensure the Xi-limited localization of Xist in differentiated cells (Rodermund et al., *Science* 2021 PMID: 34112668). Given their tight association, the number of foci of CIZ1 over the Xi is predicted to match individual Xist RNA foci observed using super-resolution microscopy, and this is indeed the case in the iXist-ChrX mESC line (the Halo-SMCHD1 parental mESC line) where CIZ1 is expressed at the endogenous level (Reviewer Figure 3a). The overexpression of CIZ1 in the Halo-SMCHD1 mESC line does not affect the number of CIZ1 foci observed (Reviewer Figure 3b), although the intensity of the foci is brighter suggesting more CIZ1 molecules are bound per Xist molecule (Reviewer Figure 3c). Since the distribution pattern of Xist across the Xi and, importantly, X-linked gene silencing are not affected (Extended Data Fig 1b), we are confident that overexpression of CIZ1 does not significantly impact XCI and downstream pathways, including SMCHD1 recruitment and functionality.

Reviewer Figure 3

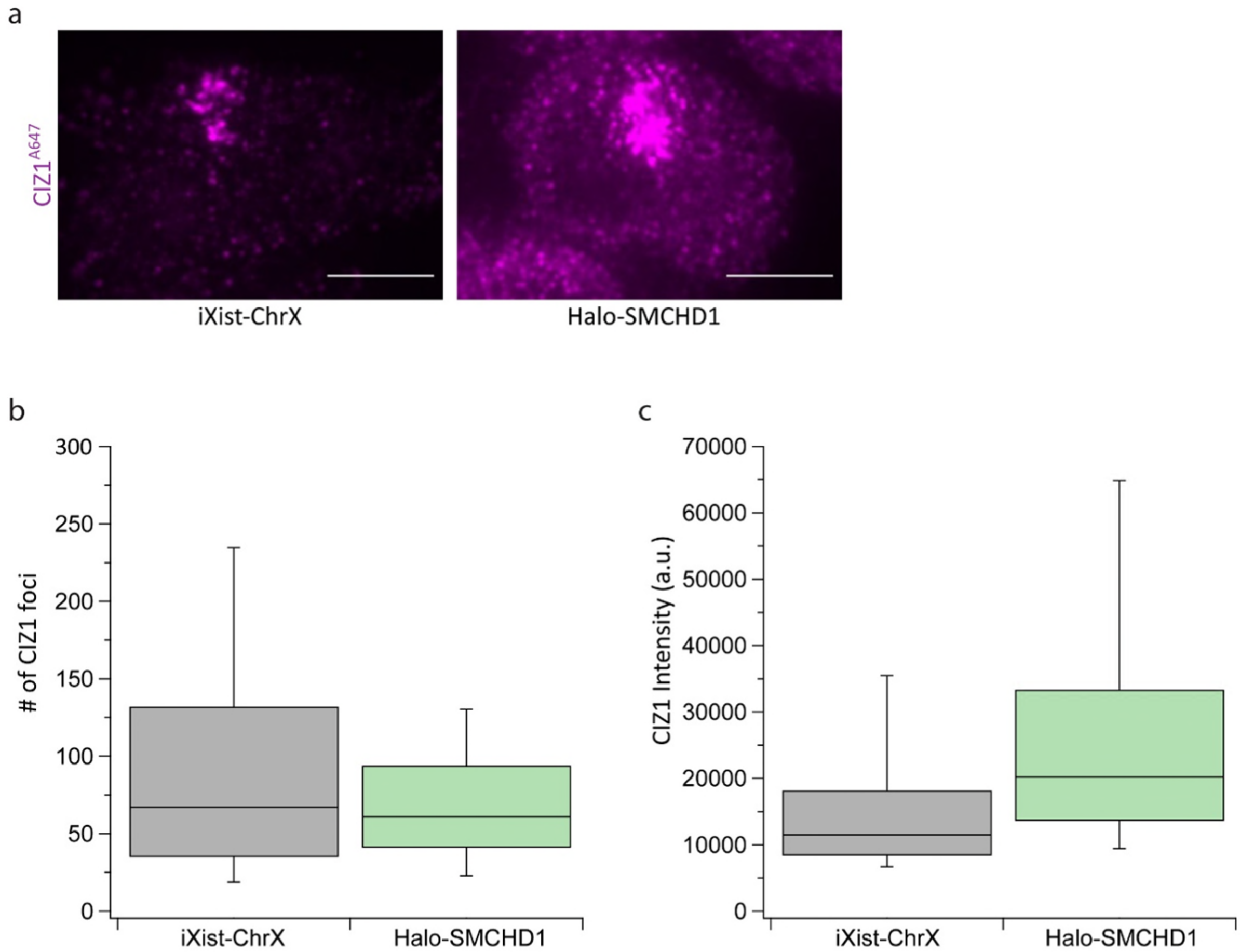

Reviewer Figure 3: Comparison of Xi-associated CIZ1 domain in the iXist-ChrX and Halo-SMCHD1 mESC lines

a. Immunostaining for CIZ1 in iXist-ChrX and Halo-SMCHD1 mESCs undifferentiated, kept for 24h with doxycycline. Images are maximum-intensity projections, scale bar - 5µm.
b. Quantification of the number of CIZ1 foci per Xi domain. n=80 cells for iXist-ChrX, n=98 cells for Halo-SMCHD1.
c. Quantification of the intensity of a Xi-associated CIZ1 foci. n=7832 foci for iXist-ChrX, n=7355 foci for Halo-SMCHD1.

5. The mechanism by which LRIF1 and ATP hydrolysis jointly contribute to gene silencing—particularly at specific loci—needs further elaboration. For example, are certain X-linked genes silenced via higher-order chromatin structure changes that are dependent on SMCHD1 loading? Allele-specific chromatin topology or long-range interaction data could strengthen this argument.

As noted above this study is not focused on analysing SMCHD1 function in gene silencing or its role in long-range Xi topology, although these questions are the subject of previous work from ourselves and others. More specifically, our prior work on the role of SMCHD1 in silencing of individual X-linked genes revealed a contribution to establishment of silencing of all X-linked genes, with a more prominent role in ensuring complete silencing of a subset (approx 25%) of these genes. This gene set

was previously characterised as requiring SMCHD1 for maintaining Xi silencing in somatic cell lines (Gdula et al., *Nat. Communications* 2019, PMID: 30604745; Sakakibara et al., *Development.* 2018, PMID: 30126901; Wang et al., *Cell* 2018, PMID: 29887375; Bowness et al., *Cell Reports* 2022, PMID: 35584662. For comments in relation to the role of chromatin topology, please see point 6 below.

6. The authors should discuss how SMCHD1-mediated gene silencing differs from canonical SMC proteins such as cohesin or condensin. Specifically, how does SMCHD1's chromatin residency and ATPase-dependent specificity contribute uniquely to silencing, as opposed to loop extrusion or chromatin compaction?

We have added to the discussion (lines 354-371) to clarify our proposed model for SMCHD1 function in long-range chromatin interactions that promote Xi gene silencing. In this context we also discuss that ATPase activity of SMCHD1 is insufficient to drive a motor function such as cohesion/condensin mediated loop extrusion.

Minor Comments

1. Extended Data Fig. 1 caption should be revised. Panel B is not a representative image but rather a comparison between Halo-SMCHD1 and the parental iXist-ChrX line. Similarly, the box plot in panel D is not an image but a quantification. The current figure legend does not accurately describe the panels and should be corrected.

Amended as suggested.

2. The histone H3 band in Extended Data Fig. 2e appears distorted or overexposed. The figure should be replaced or repeated for clarity.

We have replaced panels d and e in Supplementary Fig. 2 with one panel which shows absence of all three isoforms of LRIF1 in the *Lrif1*$^{-/-}$ mESC line. The sample preparation was optimised and whole cell lysates were used instead of nuclear fractionation so that the three isoforms can be seen more clearly. Accordingly, the histone H3 control is no longer needed.

3. The background contrast in Extended Data Figures 3d, 4f, 7a–b, and 9a is inconsistent with other figures. These images are overly bright and should be adjusted to match the visual tone of the rest of the dataset.

We have adjusted the background contrast in these figures. We would also like to point out that we are using a different LUT in these images, compared to the rest of the images, to highlight better the bleaching and recovery of the signal in the FRAP studies. Moreover, the nuclear signal in these images is indeed closer to the background level as lower exposure time had to the used in order to prevent potential phototoxicity due to the repeated imaging of cells as part of a long FRAP analysis timecourse.

4. The differential regulation of SMCHD1 recruitment to the Xi versus autosomal loci should be discussed. While LRIF1 appears to play a central role in both contexts, it remains unclear whether the recruitment mechanism is identical or context-dependent.

Protocadherin genes, which are expressed in neuronal lineages, are known autosomal targets of SMCHD1. We have performed analysis of *Pcdha1*, identified as an informative example in our

differentiation model. As shown in Reviewer Figure 4, *Pcdha1* is upregulated in SMCHD1 KO mESCs differentiated for 7 days, a pattern that is consistently observed across two biological replicates despite some replicate-to-replicate variability. Notably, a similar upregulation of *Pcdha1* is also observed in *Lrif1*$^{-/-}$ mESCs at day 7. These data support a role for LRIF1 in facilitating SMCHD1 recruitment at both Xi and autosomal loci. However, given the distinct chromatin environments of the Xi and autosomal protocadherin loci, the exact underlying mechanism remains to be investigated.

Reviewer Figure 4

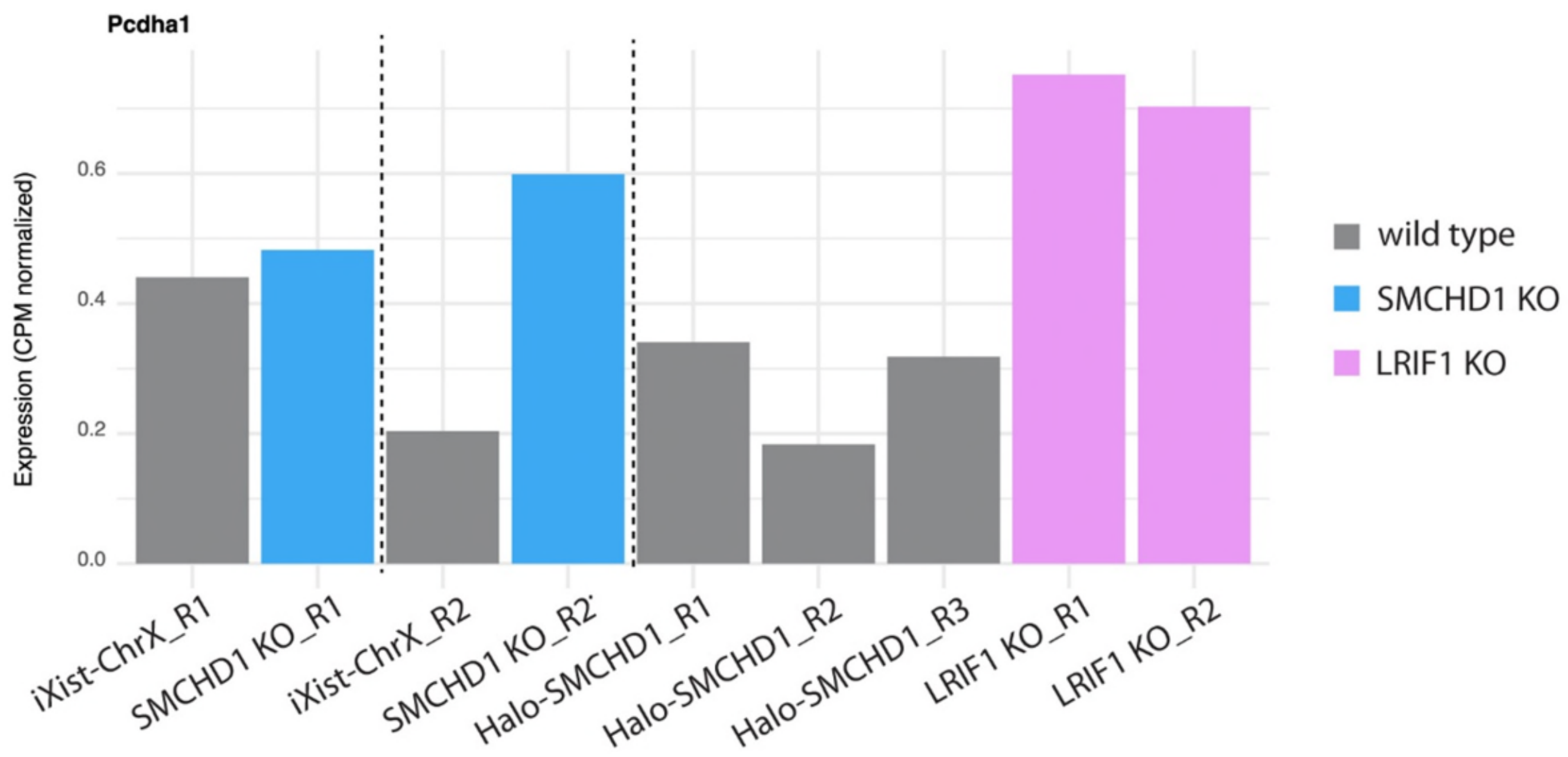

Reviewer Figure 4: Expression of *Pcdha1*

Expression of *Pchdha1* in various mESC lines differentiated for 7 days, determined using chrRNA-seq data.

5. The ATPase activities of the SMCHD1 mutants should be directly quantified in this study, rather than inferred solely from previous publications. Furthermore, the authors should consider whether LRIF1 may influence SMCHD1's ATPase activity, and if so, propose a model for how LRIF1 coupling may regulate chromatin loading through the ATPase domain.

We are not convinced that it is necessary to reproduce analysis of ATPase activity using recombinant ATPase domains. Work with recombinant proteins is not a part of the current study and the requested experiments would represent a major undertaking for our lab and also considerable time and resource. To add significant insight and novelty beyond published peer reviewed work, such studies should really be performed using full length recombinant SMCHD1 and cognate mutations, and this would be a separate study in its own right (see also comments from reviewer 2 on this point and revised text, lines 282-286). Accordingly, we consider this request to be beyond the scope of the current study. We hope that the additional mechanistic insight provided by alphafold modelling of the different mutations (reviewer 1, major point 3, reviewer figure 2) goes some way to satisfying the reviewer on this point.

Reviewer #2 (Remarks to the Author):

The study uses live imaging to study the function of SMCHD1 during X inactivation. SMCHD1 is an important protein for X inactivation and it is mutated in two human diseases with no current cures. While SMCHD1 has a GHKL ATPase domain, the function of this weak ATPase is unknown. This study uses ATPase mutants to explore the functional significance of ATP hydrolysis. As many mutations in the ATPase have been found in the ATPase domain in the human diseases, this study is timely and important.
Through live imaging, this study provides new insights into the diffusion rates and binding dynamics of SMCHD1 in vivo, which could not be gained previously from experiments with fixed cells. The authors found that SMCHD1's binding to heterochromatin and Xi is dependent on LRIF1. The authors then went on to investigate the function of ATPase activity on SMCHD1 function and performed experiments on 3 ATPase mutants. Interestingly, while both ATPase-deficient Y353C and E147A reduced SMCHD1 localization on Xi, they do so through different mechanisms. The hyperactive G137E mutant showed increased binding to Xi and greater Xi compaction. The authors concluded that ATP hydrolysis is required for selective enrichment of SMCHD1 at its target chromatin regions, which impacts its ability to silence Xi.
The study is well done with extensive quantitative analysis. My main concerns are with the changes in stability of SMCHD1 and with the interpretation of the ATPase mutant data.

Major concerns

In LRIF1 KO, SMCHD1 protein levels are reduced (Fig 2c). Why is that? Is SMCHD1 less stable when it is not bound to chromatin? The ATPase mutants also show decreases in protein stability. Can the stability be (anti-)correlated to the level of chromatin binding?

While the SMCHD1 localization changes are very clear for LRIF1 KO, there is also a reduction in total SMCHD1 levels. How do you know whether the difference is due to lower levels of SMCHD1 or due to recruitment? I have similar concerns with the E147A and Y353C mutants. Related to that, if WT SMCHD1 levels decrease in the presence of LRIF1, will that also change its chromatin localization?

We thank the reviewer for their comment. Indeed, there seems to be some level of anti-correlation between the stability of SMCHD1 and its ability to bind to chromatin. Both loss-of-function mutants and the *Lrif1*$^{-/-}$ cell line show a decrease in the bound population and/or residency time of SMCHD1 together with reduced levels of the protein, while the two gain-of-function mutants, which do not preclude SMCHD1's binding to chromatin, display normal or slightly elevated levels of the protein. However, in the loss-of-function mutants and in the cells lacking LRIF1, the changes in the dynamic behaviour of SMCHD1 are different, suggesting they are a direct consequence of the particular perturbation introduced rather than just a consequence of the reduced protein levels. To confirm this conclusion, we have now assessed the localization pattern of SMCHD1 after reducing levels using the Halo-PROTAC-E ligand in our Halo-SMCHD1 mESC line where LRIF1 is present (Reviewer Figure 5a). Indeed, as it can be appreciated from the images shown here, localization and enrichment of SMCHD1 at PCH regions and over the Xi chromosome is readily observable even with the lower levels of the protein (Reviewer Figure 5b-d).

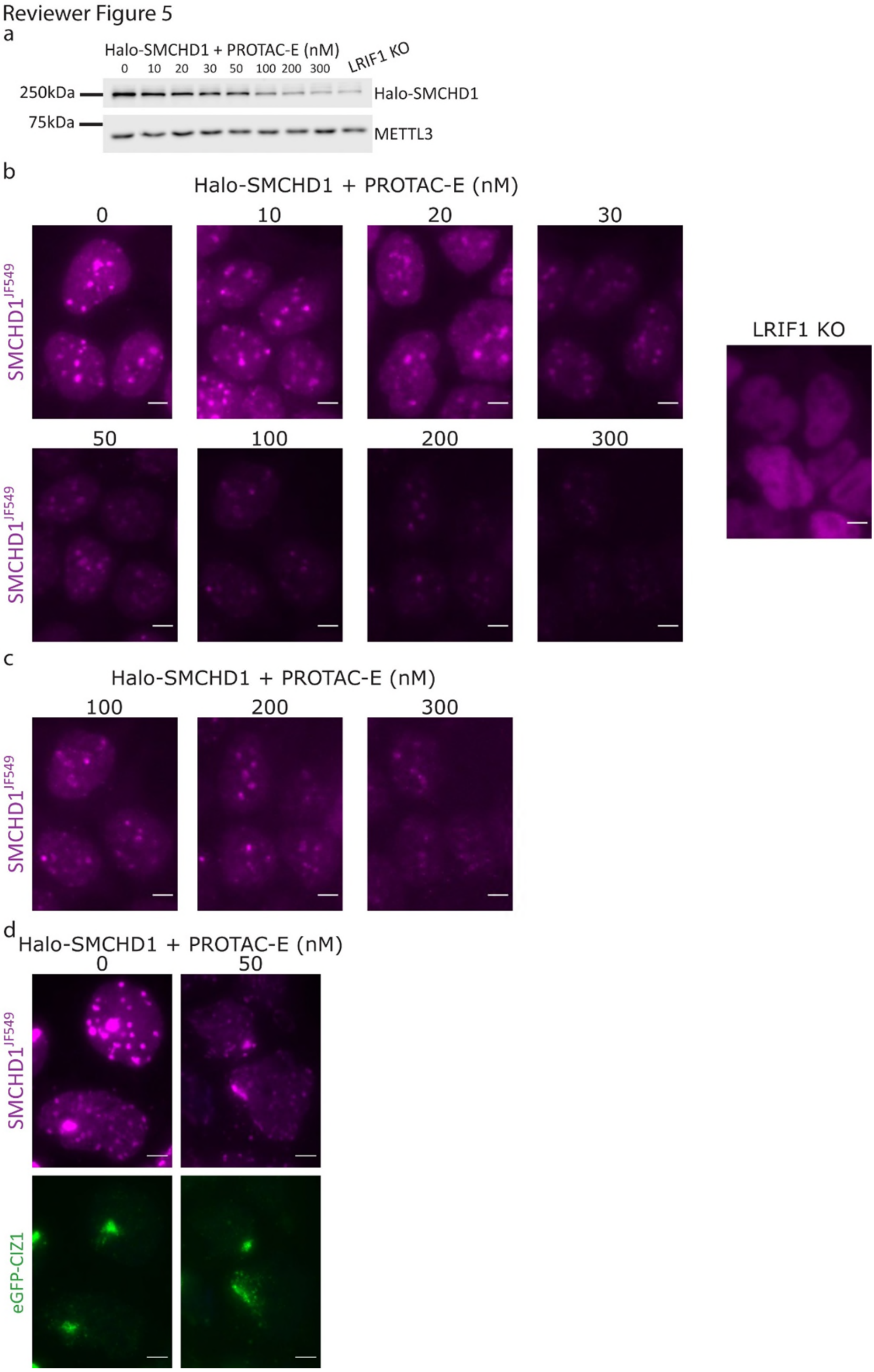

Reviewer Figure 5: Localization pattern of SMCHD1 upon reduction of its level using Halo-PROTAC-E

a. Western Blot showing reduction in SMCHD1 protein levels upon treatment with increasing concentrations of Halo-PROTAC-E.

b. Representative live-cell images of Halo-SMCHD1 mESC line treated with increasing concentrations of Halo-PROTAC-E ligand prior to labelling with JF549 and of *Lrif*$^{-/-}$ mESC line.
c. Images from b. shown with enhanced contrast to allow for better visualisation of the distribution pattern at lower level of the protein.
d. Representative live-cell images of Halo-SMCHD1 mESC line treated with 50nM Halo-PROTAC-E ligand prior to labelling with JF549 and differentiated for 7 days. All images are maximum-intensity projections. Scale bars correspond to 5µm.

Figure E4c. Why does SMCHD1 JF549 get brighter on day 3?

The images from all three days (1,3 and 7) are shown using the same contrast settings. Indeed, in the *Lrif1*$^{-/-}$ mESCs, the signal from SMCHD1 JF549 is brighter at day 3 compared to days 1 and 7, suggesting the nuclear levels of SMCHD1 increase and then decrease back to the starting level across the timecourse. The reason behind this apparent increase is not known, although it is probably linked to fluctuation of SMCHD1 levels through differentiation.

Figure 4d. Why do independent genes also show less repression in E147A?

Efficient Xist-mediated silencing is differentiation dependent. As the data referred to is from 7 day differentiated samples this minor anomaly likely reflects relatively inefficient differentiation of one of the E147A replicates. Importantly, the characteristic gradient of silencing—from SMCHD1-independent genes to partially dependent genes and then to fully SMCHD1-dependent genes—is retained in the E147A mutant. This gradient of silencing is an established hallmark of SMCHD1 deficiency in X-linked gene silencing (Gdula et al., *Nat. Communications* 2019, PMID: 30604745; Bowness et al., *Cell Reports* 2022, PMID: 35584662). Similar patterns are observed across other SMCHD1 loss-of-function alleles or full knockouts, whereas wild-type or gain-of-function contexts show little or no such gradient.

The distribution of Halo SMCHD1 and Y353C in Figure 5c looks different (less bound for Y353C), but the quantification in d shows that they are the same. Please check that the quantification is right.

We have checked and the quantification is correct. While the peak around –4 is indeed lost, it is redistributed between both the bound (see -5 to -8) and fast moving (see -1 to -3) populations and overall there is no significant change.

Does the selection of “random” nucleoplasm take into consideration the locations of PCH, for example through Hoescht staining? I would imagine SMCHD1’s dynamics on PCH to be different from nucleoplasm. Authors should be clear in their description.

ROI selection of non-Xi regions is indeed random. We have clarified this point by updating the description of our analysis pipeline in the materials and methods section (lines 571-572).

The G137E mutant is more bound to the Xi and the Xi is more compacted. Why is there no change in Xi expression? Can the authors speculate on the impact of the G137E, particularly what it may mean for understanding BAMS?

Neither the G137E nor the newly included K518E gain-of-function mutations affect gene silencing over the Xi chromosome at day 7, likely because silencing has achieved near maximal levels at this timepoint. However, SMCHD1^{K518E} does display accelerated silencing, with a higher level of X-linked gene repression being achieved at day 5 compared to the wild-type case (Figure 6c-d). In relation to this observation, one of the two replicates looking at the G137E mutant, also showed accelerated silencing at day 5 (albeit to a lower level than for K518E mutant). It may be that in the inducible Xist expression system used here, where levels are somewhat higher than in the native Xist promoter context, such differences in gene silencing are more difficult to capture. The relevance of these findings in relation to BAMS is not clear to us and investigating possible links, for example to hypomethylation of the D4Z4 locus (Shaw et al., *Nat Genet.* 2017, PMID: 28067909), will require further studies.

Previous assessments of ATPase activity were performed with just the ATPase domain alone. An assumption has been that the ATPase activity of the whole protein is similar to that of the domain alone. While that is probably true for the loss of function mutants E147A and perhaps Y353C, it is not so clear whether G137E's ATPase activity will be increased to the same extent in the full length protein, as interactions with other parts of the protein may influence ATPase activity. This assumption should be included in the manuscript.

We have added discussion of this point (lines 282-286).

The authors used 3 ATPase mutants to derive a relationship between ATP hydrolysis and SMCHD1 Xi/ chromatin localization. However it is odd that the 2 LOF mutants show different binding dynamics. E147A is less mobile in nucleoplasm while Y353C is more mobile on Xi. As such, it's not a simple relationship of less ATP hydrolysis = less binding. While the data of the mutants is interesting, the link between ATPase activity and binding dynamics is not very strong. The authors suggested that Y353C may result in the protein disengaging from chromatin rapidly following initial binding. Is there any structural evidence based on the locations of the mutations that this could happen? In my opinion, more mutants are needed to clarify the relationship between ATP hydrolysis, protein unloading and Xi binding. Alternatively, tone down on the relationship between ATP hydrolysis and chromatin binding.

We have now included the analysis of a second gain-of-function mutant, K518E. Similar to the previously analysed gain-of-function mutation G137E, it seems that increased ATP hydrolysis is associated with increased binding and retention of specific localization at SMCHD1 target loci, such as the Xi. Regarding the loss-of-function mutants, which display impaired ATP hydrolysis, both show impaired binding, but in different ways: SMCHD1^{E147A} has reduced residence time over the Xi, showing it cannot stably engage with the underlying chromatin, has lost its ability to enrich specifically at target locations and seems to be engaging non-specifically with chromatin across the whole nucleus; SMCHD1^{Y353C} is indeed not able to reach this partially stable engaged state and rapidly falls off the chromatin. To understand better the reason behind these differences we have also analysed the impact of these mutations on the interaction of the N-terminal part of SMCHD1 with ATP using AlphaFold modelling and report these findings in our response to Reviewer 1, major point 3.

The authors suggest that changes in ATPase activity directly influence the dynamics of binding to target sites on chromatin. However, the data can also be interpreted that the ATPase activity affects

SMCHD1's interactions with other adapter proteins (LRIF1 or other unknown proteins) that bring SMCHD1 to chromatin. Please address this possibility.

We agree that we cannot rule out that the SMCHD1 mutations affect interaction with LRIF1 or other unknown proteins and have added a caveat to this effect in the Discussion (lines 396-398). Please note that in relation to interactions with LRIF1 our prior work showed that the E147A mutation does not measurably change interaction with LRIF1. We infer similar for the G137E or K518E mutants in which localisation of SMCHD1 to PCH regions/Xi is not affected. For the Y353C mutation the overall nuclear bound population (which is mostly SMCHD1 loading onto PCH regions through LRIF1) is not affected, arguing that interaction with LRIF1 is not affected here either (note that in the LRIF1 KO, the bound population is dramatically decreased).

In light of the last 2 points, I suggest that the authors tone down the relationship between ATP hydrolysis and direct SMCHD1 recruitment to chromatin in the model, perhaps adding question marks to parts which are not so certain.

See response to previous two comments.

Minor concerns

Abstract: microphthalmia is spelled incorrectly.

Amended.

Line 73. It is debatable whether DUX4 expression is relevant for BAMS. And it is not clear how gain-of-function mutations in GHKL ATPase domain can lead to less repression of DUX4. This reviewer suggests to not draw this link between BAMS mutations and DUX4.

Amended.

Figure E1. The descriptions of b and d are reversed.

Amended.

Random Halo-SMCHD1 should be added to the quantification graphs of % bound population from single particle tracking experiments (eg. 3f,5g,j,6i).

Amended.

"This may seem counterintuitive given the absence of enrichment of the mutant protein over the Xi (Figure 4b, Extended Data Figure 5e), but it is key to note that the bound population of SMCHD1E147A in random regions is very similar to that seen at the Xi, both in undifferentiated and differentiated cells (Figure 5e-g)." I understand what is counterintuitive but struggle to understand the explanation. Please rephrase.

We have rephrased (lines 261-263).

Line 387. Is there any evidence that G137E disfavours ADP release? Previous in vitro results show more ADP production with G137E (PMID: 29748383), which should correspond to increased, not reduced, ADP release.

In vitro analysis of the ATPase activity of a recombinant SMCHD1 N-terminal domain harbouring the G137E indeed shows more ADP production. This can be attributed to a higher rate of ATP hydrolysis or higher tendency to bind ATP. We are in favour of the latter option due to the observed increase in the bound population of this mutant (Figure 7a-b, e-g, k-l) and the higher dimerization of recombinant N-terminal protein having this mutation (Pedersen et al., *Commun Biol.* 2019 PMID: 31312724). Since the residency time on chromatin of this mutant is also increased and AlphaFold

modelling suggests more energy may be required to unfold the mutant homodimer (see also answer to Reviewer 1, major point 3), we speculate the mutation may also disfavour ADP release.

Reviewer #3 (Remarks to the Author):

Constantinescu et al. used live-cell and single-molecule imaging to investigate SMCHD1 interactions with chromatin and its function in epigenetic silencing. They also engineered mutations in the GHKL ATPase domain of SMCHD1 and investigated their effects on SMCHD1 chromatin enrichment Xi gene repression and chromatin compaction.
The results provide interesting data addressing the selective enrichment of SMCHD1 at specific chromatin regions and gene silencing on the Xi. Nevertheless, there are a number of aspects that need to be addressed the clarify things and better support the authors claims, as listed below.

1. It is not correct that only loss-of-function mutations in SMCHD1 are present in FSHD. Indeed, the G137E gain-of-function mutation is associated to both FSHD and BAMS (PMID: 28067909).
We have amended relevant text (lines 76-77 and 286-287)

2. Lines 207-208: Contrary to the authors claims, reference 18 clearly show that LRIF1 depletion in differentiated cells affects SMCHD1 association to some Xi regions.
Text changed (now line 201-202)

3. In Figure 3g and 4b, the mutants behave similar to the WT (showing similar increase in enrichment over Xi) for the first 5 days of differentiation and then they plateau, while the WT keep increasing at day 6 and 7. Hence, SMCHD1 enrichment over Xi during differentiation appears to have a bimodal mechanism: an initial one independent by LRIF1 and unaffected by the E147A or Y353C mutation, followed by a later burst in Xi enrichment which instead is affected by the above conditions. Moreover, SMCHD1 dynamic behavior across the Xi domain is unaffected by the E147A or Y353C mutation (Figure 5e-j). It is difficult to reconcile all of this just with altered GHKL ATPase domain activity.

SMCHD1 does not accumulate over the Xi domain in LRIF1 KO and with E147A or Y353C mutations. We acknowledge that the mean intensity analysis reported in the manuscript gives the impression that SMCHD1 does accumulate during the first days of the differentiation in the LRIF1 KO and Y353C mutation context (the E147A mutant does not show accumulation with this analysis). However, the apparent increase observed in these two contexts is due to a general increase in the level of SMCHD1 over the whole nucleus as can be observed in the images in Supplementary Figure 4c and 5f. We have now quantified the level of enrichment of SMCHD1 over the Xi domain as a ratio in relation to the nucleoplasm which highlights that indeed the level of the Xi-associated SMCHD1 signal does not increase compared to the rest nucleus during the timecourse, in the LRIF1 KO and Halo-SMCHD1^{Y353C} cell lines. We want to point out though that such an analysis relies on manual selection of a nucleoplasmic region (of the same size as the corresponding Xi in that cell) and this cannot be done automatically due to the accumulation of SMCHD1 over PCH regions in the wild-type and gain-of-function mutants contexts. Therefore, we favour the analysis currently included in the manuscript as it quantifies the SMCHD1 signal over the Xi domain in an unbiased way and it does reflect whether the mutants impair or facilitate accumulation of the protein, with the associated images providing the further required information.

Reviewer Figure 6

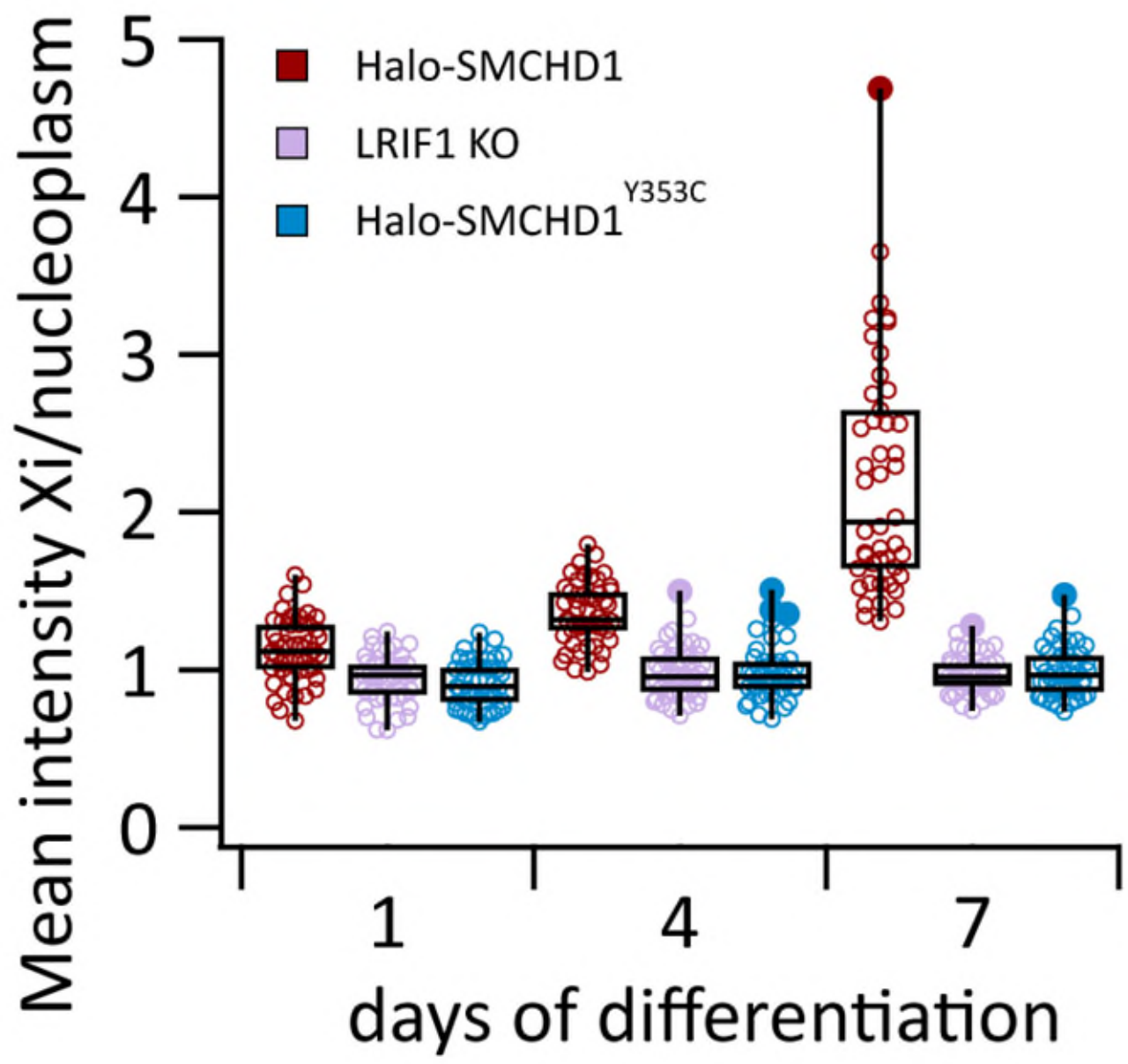

Reviewer Figure 6: Recruitment timecourse analysis using Xi/nucleoplasm ratio

Quantification of ratio between SMCHD1 signal underlying CIZ1 domains and SMCHD1 signal in a nucleoplasmic region of the same size as the CIZ1 domain, at selected timepoints during a 7-day differentiation timecourse, comparing Halo-SMCHD1 (red), Lrif1$^{-/-}$ (purple) and Halo-SMCHD1^{Y353C} (blue) mESCs. Each dot represents a cell. n = 44-50

4. The authors speculate that an interaction between prebound and newly recruited SMCHD1 could be involved in the protein enrichment over Xi. Is any of the SMCHD1 mutants used in the study affecting Xi recruitment of the WT SMCHD1? What is the mechanism responsible for the burst in SMCHD1 enrichment over Xi after day 5?

All of the SMCHD1 mutations in this study are introduced homozygously in the native locus and there is accordingly no WT protein present. Regarding the burst in enrichment after day 5, please see the response to the previous point.

5. The immunoblot of Extended Data Figure 5c,d show that the E147A and Y353C mutants significantly (it looks like 50%) reduce the SMCHD1 levels, which could affect the results of Figure 4. This caveat should be minimally discussed by the authors, better if experimentally addressed.

We have addressed this using the Halo-PROTAC-E system. Please see the reply to Reviewer 2, reviewer figure 5.

6. To better support their claims about the G137E mutant, the authors should test at least another

mutation increasing SMCHD1 catalytic activity such as A134S, S135C, E136G or (even better because located in a different part of the protein) K518E.

We have analysed another gain-of-function mutant, K518E, and the data is in accordance with the observations made for the G137E mutant. This data is now included in Figure 6, Figure 7, and Supplementary data Figures 8-10.

7. There is clearly a different behaviour of SMCHD1/LRIF1 in human and mouse cells and in ES compared to somatic cells. For example, LRIF1 does not affect SMCHD1 levels nor its recruitment to the D4Z4 locus when knock-out/knock-down in human somatic cells (PMID: 37380887), which should be discussed.

We do mention in the discussion that in somatic cells LRIF1 is not required for SMCHD1 enrichment over the Xi. The referenced work on D4Z4 is therefore in accordance with this. The reason for absence of an effect on SMCHD1 levels in the human cell models is not clear but we have validated that there is a reduction in our hands by analysing a second LRIF1 KO (see reviewer 4, comment 3).

8. For a number of graphs/quantifications (for example all Mean Xi intensity) no statistics is provided. This will be useful and in particular required whenever the authors claim there is a difference.

Amended.

9. The study is mostly descriptive as the claimed advance in mechanistic understanding of SMCHD1 function comes from correlative data.

We have removed the term ‘mechanistic’ from the abstract (line 34) to address this point.

MINOR

A. SMCHD1 has a general relevance in FSHD, given that it is also a gene modifier in FSHD1. Hence, in the manuscript, the authors could change “FSHD2” in FSHD”. Nevertheless, the molecular pathogenesis of BAMS is unclear and there is no clear indication that aberrant epigenetic regulation of D4Z4 or altered DUX4 contribute to BAMS. Hence, the authors should modify the abstract accordingly.
Amended (lines 22-24 and 75-76)

B. I’m not sure that the iXist-ChrX can be defined an interspecific line since M.m. domesticus and M.m. castaneus are not species. Indeed, they can interbreed and produce viable offspring.

Interspecific is used widely in this context, referring to F1 crosses between Mus sub-species, notably Mus Spretus, Mus Castaneus and Mus Molossinus.

C. The legend to Extended Data Figure 6 and 7 is very confusing since they are referring to panels of other Figures, i.e. e. In red, same as in Figure 3a. In green, same as in Figure 5e. f. In blue, same as in Figure 3b, etc.

Amended

Reviewer #4 (Remarks to the Author):

A potentially interesting study for understanding the detailed mechanism of SMCHD1 recruitment to chromatin (pericentromeric heterochromatin and the inactive X) in early development.
The authors created and validated a model system in which they monitored SMCHD1 subnuclear localisation by imaging in live cells. Their findings are the following:
-SMCHD1 localisation to pericentromeric heterochromatin and Xi is visualised in live cells
-LRIF1 localisation to PHC and Xi is visualised in live cells
-SMCHD1 localisation to PHC and Xi depends on LRIF1
-SMCHD1 ATP ase activity is essential for chromatin recruitment of SMCHD1

The study is well-designed, and the experimental data are well-analysed, with comparisons of different analysis methods sometimes included. The experimental data support the conclusions. However, the study does not provide substantial new biological insights into SMCHD1 chromatin recruitment, which would qualify it for publication in Nature Communications. My suggestion is to reject the manuscript and resubmit it to a lower impact journal e.g Elife.

We respectfully disagree regarding the substantial biological insights that have arisen from this work. To summarise;

1. Prior studies indicated that LRIF1 and its role in bridging SMCHD1 to H3K9me3 modified chromatin are not relevant for Xi targeting and indeed it was not clear how important this pathway is for conventional H3K9me3 sites like PCH given that SMCHD1 localisation was observed in only a small proportion of cells. Through the use of live-cell imaging approaches we have demonstrated key roles for the LRIF1 pathways in targeting SMCHD1 both to Xi and PCH.
2. By analysing the effect of different mutations in the SMCHD1 ATPase domain we provide evidence for a two-step mechanism for loading SMCHD1 at target sites in chromatin, specifically chromatin engagement facilitated by ATP binding, and chromatin consolidation, facilitated by ATP hydrolysis and ADP release. This insight is significant in terms of understanding SMCHD1 targeting to Xi and other sites, including D4Z4 repeats in the context of FSHD2.
3. Our analysis of the gain of function mutation G137E, and in the revised version K518E, provide insights into SMCHD1 functionality in promoting X-linked gene silencing and Xi chromatin compaction, again significant also in relation to autosomal SMCHD1 targets like D4Z4.

All of these advances arise from the development and application of a sophisticated live-cell imaging model to measure the dynamic behaviour of SMCHD1 and LRIF1 in the cell nucleus and specifically on the Xi chromosome. This novel platform provides a powerful tool for further mechanistic studies on these proteins and more widely in analysing other factors and pathways that contribute to XCI.

Remarks:

1. SMCHD1 G137E mutation was identified not only in BAMS but also in FSHD 2 (Lemmers et al JMG 2019)

Amended.

2. Extended Figure 1: correct labelling of the panels (panel b is panel d)

Amended.

3. LRIF1 KO clones: somatic KO of LRIF1 did not show reduced SMCHD1 (Sikrova et al 2023). Authors show only one clone for SMCHD1 blot. Show SMCHD1 levels in all 6 LRIF1 KO clones included in Extended figure 2 panel d.

From the 6 LRIF1 KO clones shown in Extended figure 2 panel d, only two were confirmed as usable for downstream analysis of LRIF1 knockout. We show in Reviewer Figure 7 that SMCHD1 levels are also reduced in the second independent clone.

Reviewer Figure 7

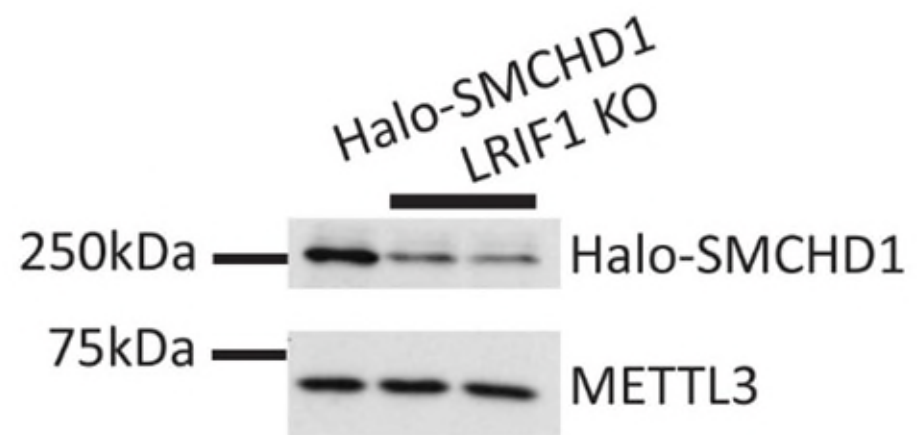

Reviewer Figure 7:

Western blot showing SMCHD1 protein levels in the absence of LRIF1 in two independent clones.

4. The authors claim that LRIF1 recruits SMCHD1 to PCH, supporting their claim by showing the missing SMCHD1 signal at PCH in Figure 2, panel B. However, this sample has a very low level of SMCHD1, so it is possible that at this low level, SMCHD1 is not detectable.

The WT and LRIF1 KO images are set to the same contrast and SMCHD1 is clearly detectable via Halo labelling with the JF549 ligand, but it does display a different localization pattern in the absence of LRIF1, when it is no longer able to enrich at PCH regions. Importantly, this change is not due to the decreased levels of SMCHD1: upon decreasing the levels of SMCHD1 using the Halo-PROTAC-E ligand while LRIF1 is present, SMCHD1 is still able to visibly enrich at PCH regions (please see data included for Reviewer 2, reviewer Figure 5).

5. Extended figure 2 panel E: it seems that in the LRIF1 KO sample the 2 short isoforms of LRIF1 are present, while on panel D these bands are not present. Does panel E show an LRIF1 KO sample?

We have included an improved Figure panel obtained using an optimised protein extraction protocol that enables visualisation of all three isoforms being removed in LRIF1 KO.

6. Earlier studies claim that dimerisation of SMCHD1 is essential for chromatin binding. Can G137E, E147A or Y353C mutants form dimers in the described model system?

Dimerisation of SMCHD1 is mediated by both N-terminal GHKL ATPase domains and C-terminal SMC

hinge domains (Brideau et al., *Mol Cell Biol.* 2015 PMID: 26391951; Chen et al., *Biochem J.* 2016 PMID: 26733688; Pedersen et al., *Commun Biol.* 2019 PMID: 31312724). The latter should not be affected by GHKL ATPase mutations. SMCHD1^{G137E} is able to bind to chromatin as shown both by SPT and FRAP studies and it is able to facilitate X-linked gene silencing which suggest that the mutation does not impair dimerization of the protein. We have previously shown that the E147A mutation does not impair dimerization of SMCHD1 (Brideau et al., *Mol Cell Biol.* 2015 PMID: 26391951) and, in this study, SPT and FRAP data show the mutant is able to bind to chromatin, but with an altered residence time and reduced specificity for its target locations, leading to its inability to ensure gene silencing over the Xi chromosome. Y353C does lead to a dramatic decrease in the residence time of SMCHD1 on chromatin, but its bound population is not affected which suggests the mutant protein is able to undergo homodimerization.

REVIEWERS' COMMENTS

Reviewer #1 (Remarks to the Author):

The current manuscript is publishable
Nothing to address.

Reviewer #2 (Remarks to the Author):

The authors have done a tremendous job in the revision. I am very happy that they have included an additional mutant, which strengthened the link between ATPase activity and recruitment to target sites. I recommend accepting the manuscript.
Some of the reviewer figures are particularly informative. Reviewer Figure 5 is a very good control and I recommend including it in the manuscript as a supplemental figure. I also suggest to summarize the Alphafold analysis and include it in the manuscript to explain the difference between the 2 LOF mutations.

The AlphaFold analysis and Reviewer Figure 5 are now included in the manuscript.

Reviewer #3 (Remarks to the Author):

The authors satisfactorily addressed my concerns. Congratulation on a nice pice of work!
Nothing to address.

Reviewer #4 (Remarks to the Author):

The authors of the manuscript "Selective interaction of the protein SMCHD1 with specific chromatin regions is governed by the loading factor LRIF1 and SMCHD1 ATPase activity" provided a thorough and well-constructed rebuttal. Their responses to the reviewers' comments, along with the inclusion of additional data—particularly the chromatin binding analysis of the K518E gain-of-function SMCHD1 mutant—and substantial contextual revisions, have markedly strengthened the manuscript. I support publishing it in Nature Communications after minor corrections.
1. Raw 70:" A notable case is the gene encoding DUX4 in human, abnormal expression of which results in FSHD2."
DUX4 is expressed not only in FSHD2 but also in FSHD1. I suggest correcting FSHD2 to FSHD.

Amended.

2. Raw 72: “FSHD2 mutations have also been found in a second factor, LRIF1,” There is only one LRIF1 mutation published; the mutation should be corrected to mutation.

Amended.
